# ZEB1 regulates glioma stemness through LIF repression

**DOI:** 10.1038/s41598-017-00106-x

**Published:** 2017-02-28

**Authors:** Lincoln A. Edwards, Aiguo Li, Dror Berel, Mecca Madany, Nam-Ho Kim, Minzhi Liu, Mitch Hymowitz, Benjamin Uy, Rachel Jung, Minlin Xu, Keith L. Black, Altan Rentsendorj, Xuemo Fan, Wei Zhang, John S. Yu

**Affiliations:** 1 Department of Neurosurgery, Cedars-Sinai Medical Center, Los Angeles, California USA; 2 Neuro-Oncology Branch, National Cancer Institute, Bethesda, MD USA; 3 Biostatistics Core, Cedars-Sinai Medical Center, Los Angeles, California USA; 4 Pathology and Laboratory Medicine, Cedars-Sinai Medical Center, Los Angeles, California USA

## Abstract

The identification of a stem cell regulatory gene which is aberrantly expressed in glioma and associated with patient survival would increase the understanding of the role of glioma cancer stem cells (GCSCs) in the virulence of gliomas. Interrogating the genomes of over 4000 brain cancers we identified *ZEB1* deletion in ~15% (grade II and III) and 50% of glioblastomas. Meta-analysis of *ZEB1* copy number status in 2,988 cases of glioma revealed disruptive *ZEB1* deletions associated with decreased survival. We identified *ZEB1* binding sites within the LIF (stemness factor) promoter region, and demonstrate LIF repression by ZEB1. ZEB1 knockdown in GCSCs caused *LIF* induction commensurate with GCSC self-renewal and inhibition of differentiation. IFN-γ treatment to GCSCs induced ZEB1 expression, attenuating LIF activities. These findings implicate *ZEB1* as a stem cell regulator in glioma which when deleted leads to increased stemness, tumorigenicity and shortened patient survival.

## Introduction

The genetic underpinnings of how glioma cancer stem cells (GCSCs) propagate tumors and how this affects patient survival is not well understood. Identifying genes that control stem cell regulation, especially those in which mutations or a loss in copy number of these stem cell regulatory genes can support the propagation of the cancer, is fundamental to the basic understanding of brain cancer lethality. To address this question, we utilized 2,988 brain cancer genomes for copy number analysis, 339 glioma genomes for mutations indicative of loss of function and 1,007 gliomas for mRNA expression analysis. Our primary focus involved searching for genes showing enrichment for copy number loss, loss of heterozygosity (LOH) and mutations. We identified several genes which have previously been described in glioblastoma (GBM) and lower grade (WHO grade II and grade III) gliomas such as *PTEN*, *NF1* and *IDH1* and concentrated our efforts on a gene not previously implicated to have copy number loss, LOH or mutations in GBMs or low grade gliomas namely, Zinc Finger E-Box Binding Homeobox 1 gene (*ZEB1*). *ZEB1* is an inducer of the epithelial-mesenchymal transition (EMT) in cancers^1, 2^ and has been shown to promote cancer invasion in glioblastomas among other cancers^3^. Most insights into its action would suggest that ZEB1 expression would be associated with a negative outcome in cancer patients based on its role in increasing tumorigenicity and stemness^4–7^. We have identified *ZEB1* as a stem cell regulator in brain cancer which when deleted leads to increased stemness, tumorigenicity and shortened patient survival. Although evidence of decreased ZEB1 expression and deletion does exist^8–10^, studies using The Cancer Genome Atlas (TCGA) datasets have not revealed decreased expression or loss of the *ZEB1* gene either by copy number or mutation, particularly not in brain cancer^11, 12^. In contrast, we have observed *ZEB1* deletions in more than 50% of GBMs and 15% in low grade gliomas (grade II and grade III) with frequent LOH. The explanation for this discordance is that both GBMs and low grade gliomas do not demonstrate the more frequently observed and investigated homozygous or deep deletions, rather GBM patients and low grade glioma patients have heterozygous or shallow deletions of *ZEB1*. In addition, our analysis of glioma patients from our institution through exome sequencing revealed previously unidentified mutations. These mutations along with other recently observed mutations of *ZEB1* in gliomas could account for the decreased ZEB1 expression^13, 14^. These findings uncover important information about stem cell regulation by ZEB1 expression, copy number level in both GBMs and low grade gliomas with implications for prognostication and treatment of gliomas.

## Results

### *ZEB1* Copy number loss and Loss of Heterozygosity

**Figure 1:**
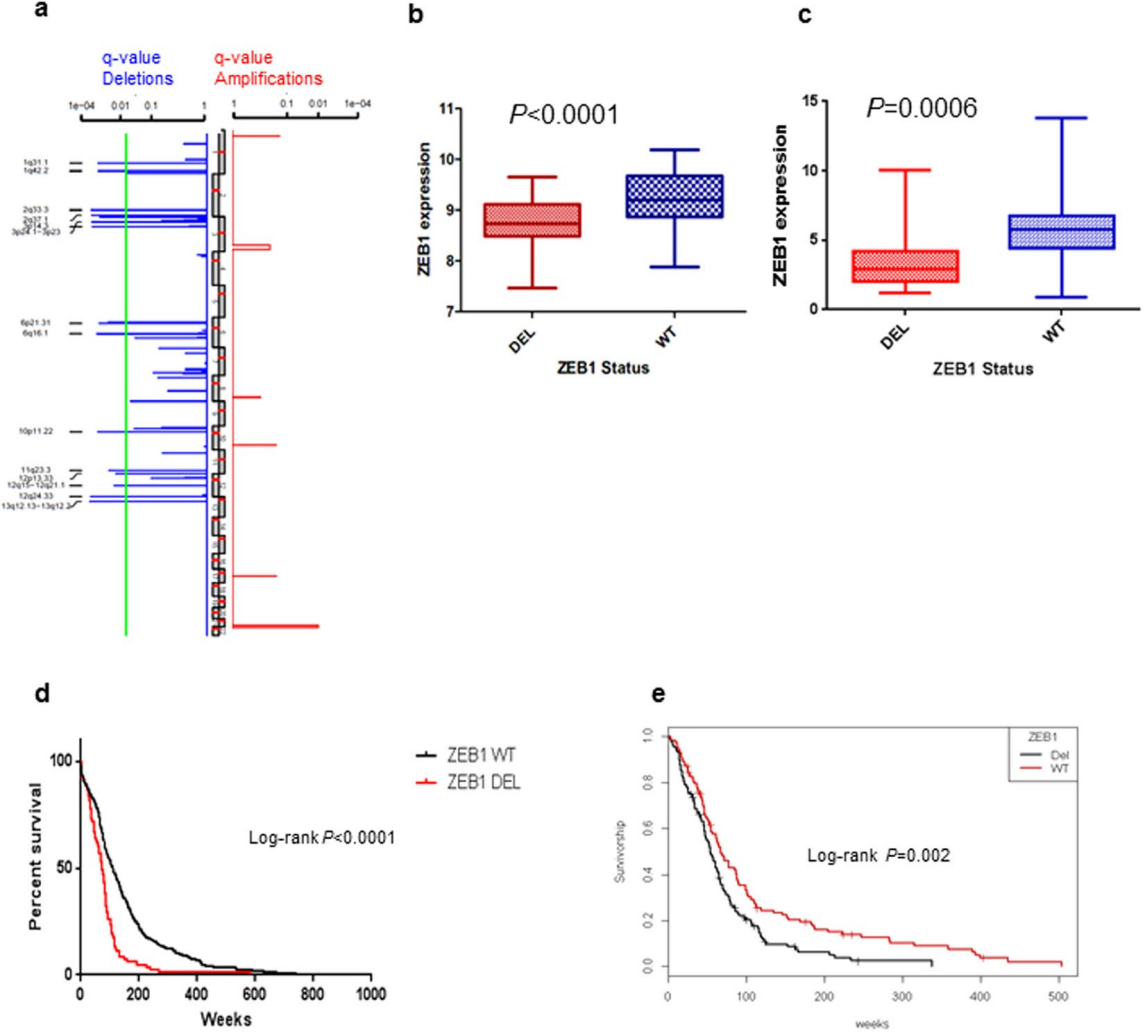
Somatic Copy Number Alterations. (**a**) Copy number alterations determined for 70 low grade gliomas (grade II and III) by single nucleotide polymorphism (SNP) arrays. Significant amplifications (red) and deletions (blue) were determined for the chromosomal regions and are plotted as q-values (significance <0.05). (**b**) *ZEB1* deletion (DEL) for glioblastomas, defined as copy number less than or equal to −0.5 (*n* = 188); wildtype (WT) defined as copy number greater than or equal to zero (*n* = 62). Two-tailed student’s t-test identified a significant difference between these two groups ***P < 0.0001. (**c**) *ZEB1* deletion (DEL) for low grade gliomas (*n* = 79); wildtype (WT) (*n* = 372), copy number was previously called for DEL or WT in cBioportal. Two-tailed student’s t-test identified a significant difference between these two groups **P = 0.0006. (**d**) Estimated Kaplan-Meier survival curves for 451 low grade glioma patients (left) for deleted (DEL) and wildtype (WT) copy number. Patients with low grade gliomas having DEL vs. WT ZEB1 had estimated median survival times of 16.82 vs. 25.46 months. *P*-value was determined by log rank test ***P < 0.0001, hazard ratio 1.96. (**e**) Kaplan-Meier estimates of overall survival of *ZEB1* WT GBM patients compared to *ZEB1* DEL patients (*n* = 238). *P*-value was determined by log rank test **P = 0.002, hazard ratio 1.54.

In order to investigate possible heterozygous deletions, we used the CGARS^15^ algorithm which transforms raw copy number into ranks, thereby avoiding copy number base line levels. Using the CGARS^15^ algorithm, we identified significant focal copy number alterations and observed deletions affecting 10p11.22, the *ZEB1* locus in lower grade gliomas (grade II and III, *q*-values [False discovery rate] <0.001, Fig. 1a). Similarly, cBioportal^16, 17^ revealed in both lower grade gliomas consisting of WHO grade II and grade III gliomas (n = 527) and GBMs (n = 595) significant heterozygous deletions indicated as shallow deletions (data not shown). In over 50% of glioma cases, we observed a deletion that included *ZEB1* on chromosome 10 (Fig. S1a and Table S1). Copy number alterations in *ZEB1* could be identified in both primary and recurrent (*n* = 87) GBM patients in relation to well characterized genes determined by the TCGA GBM Analysis working group (Fig. S1b,c). Analysis of TCGA data for copy number along with expression data where we correlated expression and copy number data, revealed a significant decrease in ZEB1 expression in both GBM and low grade glioma patients (Fig. 1b,c, *P* < 0.0001 and *P* = 0.0006 respectively). Comparing the whole of chromosome 10 and the specific *ZEB1* locus in glioblastoma patients indicated significant copy number loss (Fig. S1d,e, respectively) relative to patient blood with normal copy number (Fig. S1f). Expression levels varied across the major glioma histologic subtypes (Fig. S1g). Importantly, copy number loss of ZEB1 correlated with shortened patient survival in both lower grade gliomas (***P < 0.0001) and GBMs (**P = 0.002, Fig. 1d,e respectively). In addition, data from the COSMIC^18^ database (data freeze Jan 2014) also revealed significant copy number loss where 110 of 138 GBMs (79.7%) had copy number loss at the *ZEB1* locus (Table S2). Taken together these data suggest that *ZEB1* loss is an important prognostic indicator and is associated with unfavorable outcome for both lower grade gliomas and GBM patients.

**Figure 2:**
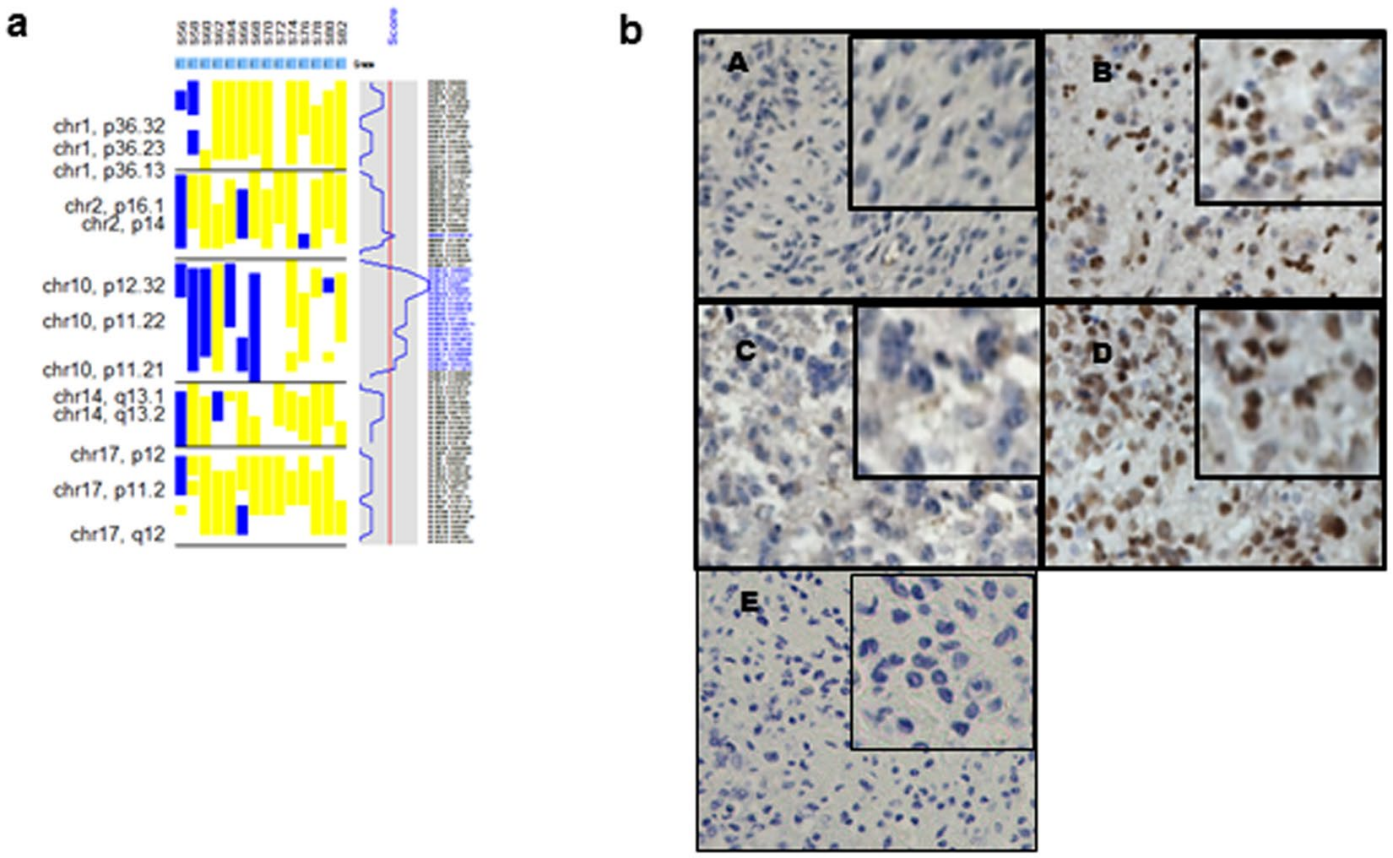
Decrease or loss of ZEB1 expression defines LOH. (**a**) Identification of LOH (*n* = 14) with glioblastoma and matched controls within the *ZEB1* locus (10p.11.2) LOH could be inferred in a retrospective SNP analysis of GBM patients indicated by colors yellow-no LOH, blue-LOH in 29% of patients. (**b**) Representative immunohistochemistry from tissue microarrays. Scale bar represents 100 μm.

Given the heterozygous nature of the observed copy number loss in both lower grade and GBM patients for ZEB1, we set out to determine if loss of heterozygosity (LOH) was present at the *ZEB1* locus. Analysis of an initial 14 GBM patients with matched normal controls at our institution identified LOH in approximately 29% of patients (Fig. 2a). We further expanded this analysis to 178 glioblastoma patients (both datasets taken from the Gene Omnibus Expression database)^19, 20^ and found LOH in 22% of patients (Fig. S2a). We sequenced all exons of −14 GBM patients matched for tumor and blood plasma from samples at Cedars-Sinai Medical Center. Sanger sequencing also revealed LOH at the *ZEB1* locus in 21% (3/14) of samples (Table S3 and Fig. S2b). Lastly, GBM patient samples from Cedars-Sinai Medical Center were analyzed for whole genome copy number where we validated LOH at the *ZEB1* locus (Table S4). In addition, glioma patient derived GCSCs (0827) also revealed LOH (Fig. S2c). Collectively, we examined two independent datasets, as well as in house GBM patient samples with matching blood plasma from Cedars-Sinai Medical Center and patient derived GCSCs to validate LOH by Sanger sequencing.

Having examined four independent datasets for genome wide copy number and two datasets for LOH, and confirmed LOH in our own Cedars-Sinai Medical Center patients through Sanger sequencing, we wanted to determine if *ZEB1* gene loss would lead to ZEB1 protein loss. To confirm ZEB1 loss at the protein level, we performed immunohistochemistry using tissue microarrays (Fig. 2b), which revealed the presence (Fig. 2b_b_ and b_d_) and absence (Fig. 2b_a_ and b_c_) of ZEB1 when compared to control (Fig. 2b_e_) in grade 4 GBMs consistent with the loss of ZEB1 in certain patients and the preservation of ZEB1 in other GBM patients. Only 11% of GBMs analyzed had strong nuclear staining, with 58% of GBM patients having weak to no staining, 28% having moderate staining and 3% of GBM patients were unscored due to poor quality tissue. Analysis of our patient derived GCSCs also confirmed that the majority of the lines have low expression of the ZEB1 protein (Fig. S3a).

### ZEB1 Mutations

**Figure 3:**
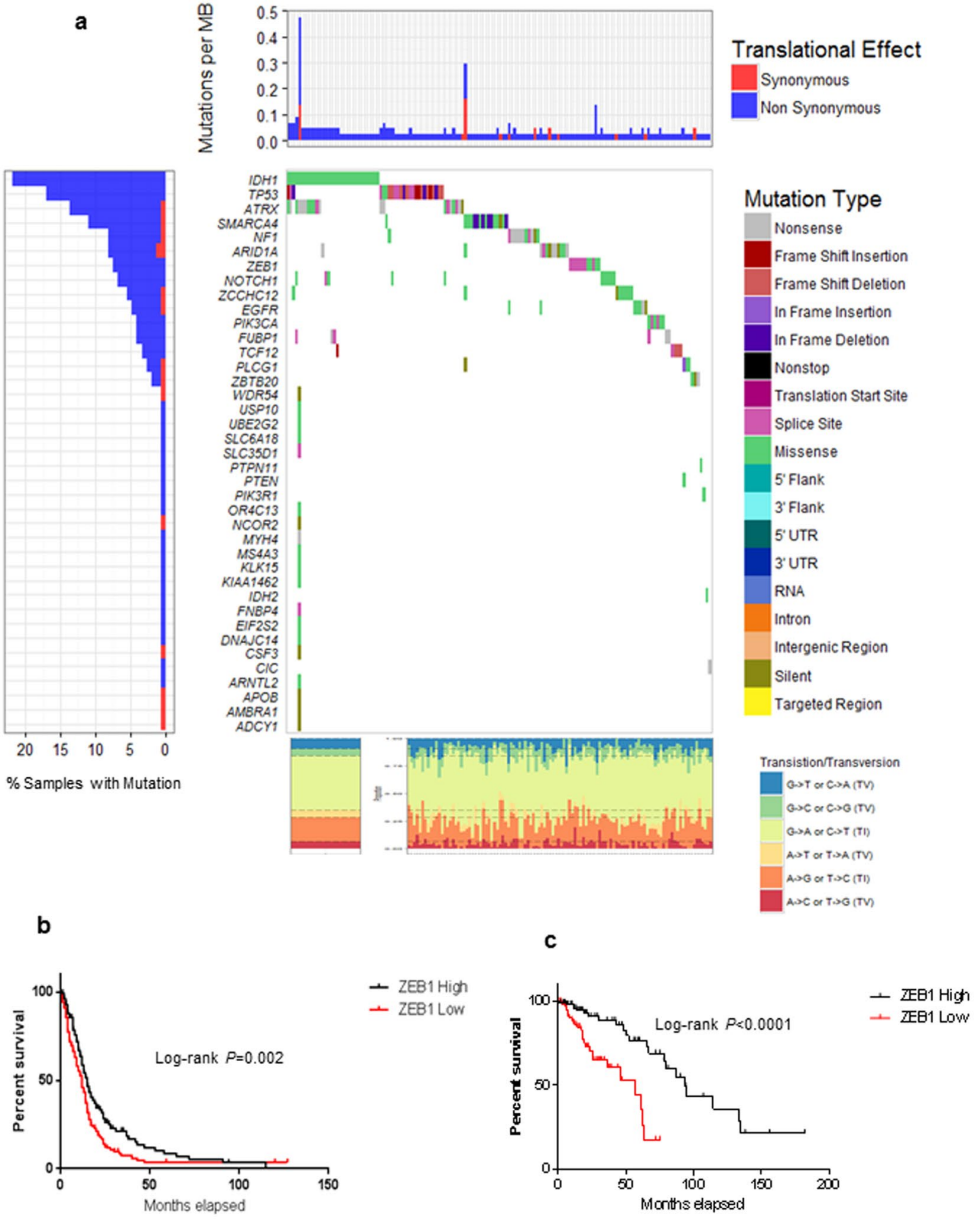
Genomic alterations in gliomas. (**a**) Glioma samples are arranged from left to right. Alterations of low grade gliomas and GBM candidate genes are annotated for each sample according to the color panel (right). The somatic mutation frequencies for each candidate gene are plotted on the left panel. Mutation rates and type of base-pair substitution are displayed in the top and bottom panel, respectively. (**b**) Estimated Kaplan-Meier survival curves for 507 glioblastoma patients (left) for high and low ZEB1 expression. Patients with GBMs having high vs. low ZEB1 expression had estimated median survival times of 580 vs. 310 weeks. *P* -value was determined by log rank test *P = 0.002, hazard ratio 1.25. (**c**) Estimated Kaplan-Meier survival curves for 249 low grade gliomas patients (right) for high and low ZEB1 expression. *P*-value was determined by log rank test ***P < 0.0001, hazard ratio 3.341.

Having identified copy number loss as a means of ZEB1 loss we turned our attention to mutations that may be affecting ZEB1 expression. To comprehensively characterize mutations that affect gliomas we enriched for ZEB1 by combining low grade glioma data, GBM patient data including previously reported *ZEB1* mutations^13, 14^, and exome sequencing data from Cedars-Sinai Medical Center. The data analyzed consisted of an initial 203 samples representing already reported mutations (*n* = 7), GBMs (*n* = 108) and lower grade gliomas (grade II and grade III) (*n* = 88). Of the initial 203 samples 41 were excluded from the analysis because of insufficient quality or amount of DNA or insufficient information for analysis, these were all GBMs. Somatic single-nucleotide variants (SSNVs) were called by comparison to the NCI build 37, with a median of 19 SSNVs identified per sample (range of 3 to 877). G > A and C > T transitions made up the bulk of the mutations accounting for 61% collectively with 3% or more mutations occurring in 38% of the genes listed. The bulk of the genes were characterized by missense mutations (Fig. 3a). Although it is unclear the impact of the identified *ZEB1* mutations, the degree to which ZEB1 mutations occur suggests that these mutations contribute to *ZEB1* loss in both lower grade gliomas and GBMs. In support of ZEB1 mutations carrying out a pro-tumorigenic function, the top 10 genes which included well known contributors to both GBMs and low grade gliomas such as *IDH1*, *TP53*, *NF1* and *ZEB1* were for the most part mutually exclusive and strongly associated with missense or splice site mutations (Fig. 3a).

Given the copy number loss and increased number of mutations now identified for ZEB1, we sought to determine if there was a relationship between ZEB1 expression and survival in either lower grade glioma or GBM. Consistent with our observation of poor patient survival due to *ZEB1* deletion (Fig. 1b,c); patients with low ZEB1 expression resulted in shorter patient survival in both lower grade gliomas (***P < 0.0001, Fig. 3c) and GBMs (*P = 0.002, Fig. 3b).

### ZEB1 loss increases GCSC stemness

**Figure 4:**
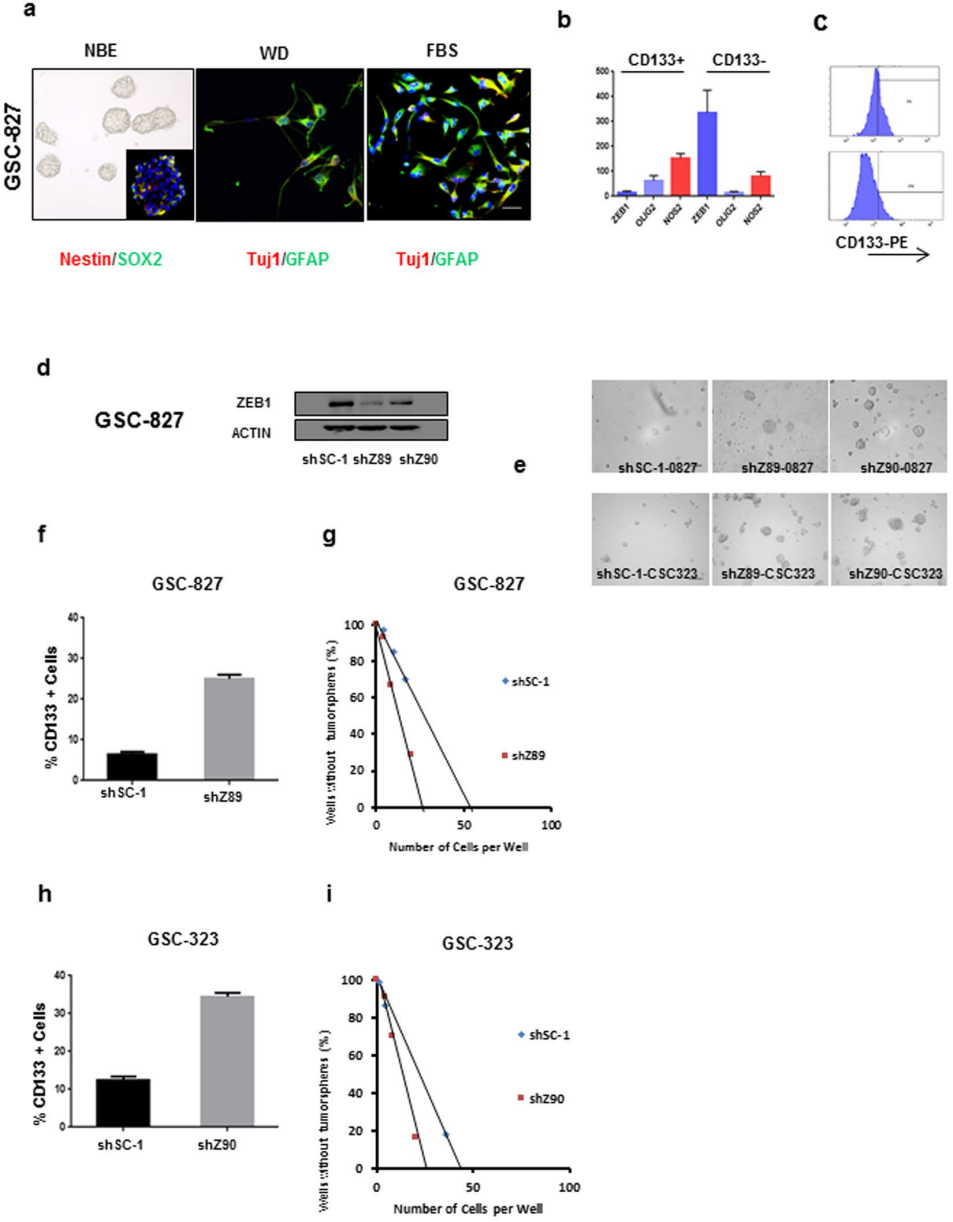
ZEB1 loss enhances stemness in GCSCs. (**a**) Left, GCSCs forming neurospheres and expressing Nestin and Sox2. Middle, Right, GCSCs induced to differentiate expressing TUJ1 and GFAP. NBE = Neural Basal A media, WD = growth factor withdrawal, FBS = fetal bovine serum. Scale bar represents 100 μm (**b**) real time qRT-PCR expression of ZEB1, OLIG2 and NOS2 in enriched CD133 GCSCs (wildtype ZEB1) and matched CD133 depleted GCSCs. (**c**) Top panel, Flow cytometry of GCSCs indicates CD133 positivity in contrast to GCSCs that were cultured under differentiation conditions that do not. (**d**) Western blot indicating GCSC 827 ZEB1 knockdown with shRNA targeting ZEB1 (shZ89, shZ90) or a non-targeting scrambled control (shSC-1, top western right). (**e**) Neurosphere morphology of GCSCs targeted with either ZEB1 shRNAs (shZ89, shZ90) or non-targeting shRNAs (shSC-1). **(f)** The percentage of CD133+ 827 GCSCs targeted with either ZEB1 targeted shRNAs (shZ89 or shZ90) or non-targeting shRNAs (shSC-1) were determined by flow cytometry. **(g)** Limiting dilution sphere-forming assay indicated that cells transduced with shRNAs targeting ZEB1 (shZ89 or shZ90) increased self-renewal *in vitro*. **(h,i)** Same as **(f,g)** only 0323 GCSCs were used.

Given the deleterious effects of ZEB1 loss on patient survival, we wanted to determine if loss of ZEB1 was associated with increased stemness as the basis and link to both tumor virulence and poor patient survival. We utilized CD133, a cell surface marker used to prospectively identify and isolate glioma cancer stem cells^21–24^. Examining GBM patient tumors (*n* = 269) for copy number and gene expression data revealed that *ZEB1* deleted tumors demonstrated increased CD133 expression compared to *ZEB1* wildtype tumors (Fig. S3b, *P* = 0.023). We next turned to glioma patient derived cancer stem cells (GCSCs), and undertook a series of studies to characterize their stem cell properties. GCSCs grown under stem cell conditions, select against survival of terminally differentiated cells, maintaining neurosphere fidelity (Figs 4a, S3c left) and GCSC marker expression (inset). Consistent with previous studies^21, 24^ we further demonstrated that GCSCs have the potential to differentiate along neuronal and/or glial lineages (Figs 4a, S3c middle and right respectively). To validate our GCSCs we used magnetically activated cell sorting (MACs) to acutely isolate GCSCs into CD133^+^ and CD133^−^ populations and observed high expression of the reported stem cell markers OLIG2 and NOS2^25, 26^ in CD133^+^ GCSC populations along with low ZEB1 expression. In contrast, CD133^−^ GCSC populations had low levels of NOS2 and OLIG2 with high levels of ZEB1 expression (Fig. 4b). GCSC expression of CD133 (Fig. 4c, top) was eliminated when GCSCs were cultured under differentiation conditions (Fig. 4c, bottom) consistent with what has been reported^27^. A hallmark feature of GCSCs is their tumorigenic potential. Implantation of our GCSCs in an orthotopic xenograft mouse model resulted in brain tumor formation (Fig. S3d). Primary GBMs and patient derived GCSCs revealed, that the majority expressed low levels of ZEB1 inversely correlating with CD133 expression as determined by RT-PCR (Fig. S3e,f). This led us to investigate whether knockdown of ZEB1 (Figs 4d, S3g) would maintain or enhance stem cell properties. Suppression of ZEB1 expression using shRNAs revealed a significant increase in neurosphere size, the CD133^+^ subpopulation (6.4% vs 25% ± 1.8%), and self-renewal compared to non-targeting shRNAs in GCSCs (Fig. 4e–i).

**Figure 5:**
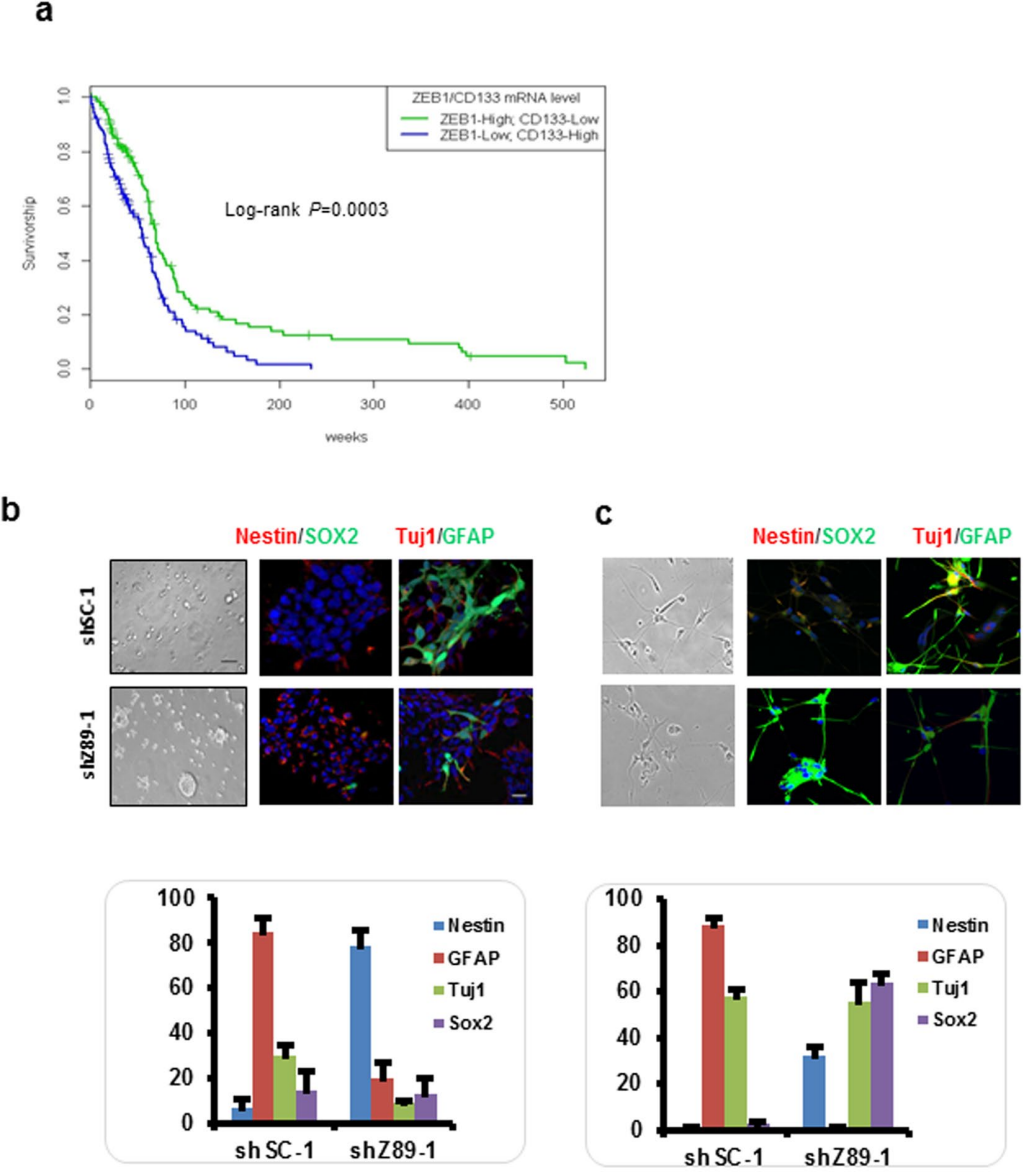
ZEB1 loss enhances resistance to differentiation. (**a**) Determining ZEB1 expression stratified for CD133 expression (*n* = 251). Median survivals were 540 weeks for the high CD133, low ZEB1 group vs. 220 weeks for the low CD133, high ZEB1 group. *P*-value was determined by log rank test ***P = 0.0003 hazard, ratio 1.73. (**b**,**c**) Immunofluorescent micrographs of 827 GCSCs (**b**) and 55 GCSCs (**c**) transduced with either shSC-1 (top) or shZ89 (bottom) under differentiation conditions. Differentiation was inhibited in ZEB1 targeted shZ89 GCSCs. Scale bars represent 60 μm. Bottom, histograms indicating the percentage of Nestin, Tuj1, GFAP and Sox2 positive 827 and 55 GCSCs transduced with either shSC-1 or shZ89. Error bars represent the mean of ± SEM of at least 3 experiments.

The loss of ZEB1 expression was associated with an increase in CD133 expression in GBM patient tumors. In addition, the loss of ZEB1 led to an increase in CD133 expression in our GCSCs. This encouraged us to determine whether ZEB1 loss was associated with high CD133 expression and would result in a worsened patient outcome. Indeed, when loss of ZEB1 expression was stratified with CD133 expression (Fig. 5a, hazard ratio 1.73, 0.95% CI, 1.28–2.34; **P = 0.0003) the result was shortened patient survival, suggesting that the effect of ZEB1 loss on survival was consistent with an increase in the proportion of the glioma stem cell population in the tumor.

To examine ZEB1 loss and resistance to differentiation, we compared targeting ZEB1 using shRNAs in GCSCs to non-targeting shRNAs in GCSCs. Non-targeting shRNAs in GCSCs placed in culture conditions conducive to differentiation resulted in cell morphology changes (Fig. 5b,c top left) starting with decreased expression in Nestin in both lines and a decrease in Sox2 in one of the lines (Fig. 5b,c, top-middle and lower panel quantification). Reciprocally, there was a significant increase in end terminal differentiation markers for astrocytes (GFAP) and neurons (Tuj1) (Fig. 5b,c, top-right and lower panel quantification). Knockdown of ZEB1 in GCSC 827s exposed to the same differentiation conditions led to little change in morphology (Fig. 5b, bottom-left) with over 78% of infected 827 GCSCs maintaining their Nestin expression (Fig. 5b, bottom-middle and lower panel quantification) while there was little increase in GFAP or Tuj1 (Fig. 5b, bottom-right and lower panel quantification). Infected GCSC 55  resulted in over 30% and 65% retaining their Nestin and Sox2 expression respectively (Fig. 5c, bottom-middle and lower panel quantification). Similar to the 827, there was little increase in GFAP (Fig. 5c, bottom-right and lower panel quantification) however there was an increase in Tuj1. These findings indicate that loss of ZEB1 expression led to the maintenance of the GCSC-like state and resistance to differentiation.

It has been reported that certain stem cell factors can block differentiation, essentially conferring resistance to differentiation, allowing cancer stem cells to proliferate and continue tumor propagation even under differentiation conditions^28^. To investigate if loss of ZEB1 would confer GCSC resistance to differentiation, we cultured GCSCs under conditions of maintaining the stem cell-like state and alternatively, under differentiation conditions. We saw a significant decrease in cell proliferation of our GCSC targeted with non-targeting shRNAs under differentiation conditions, however, GCSCs infected with ZEB1 targeting shRNAs maintained a similarly high proliferative rate in differentiation conditions (Fig. S3h). Under normal stem cell media conditions both our GCSCs targeted with either non-targeting or ZEB1 targeting shRNAs were similar. These data support our conclusion that decreased expression of ZEB1 enhances or at least maintains the cancer stem cell-like state even under differentiation conditions.

### IFN-γ induces ZEB1 activation

**Figure 6:**
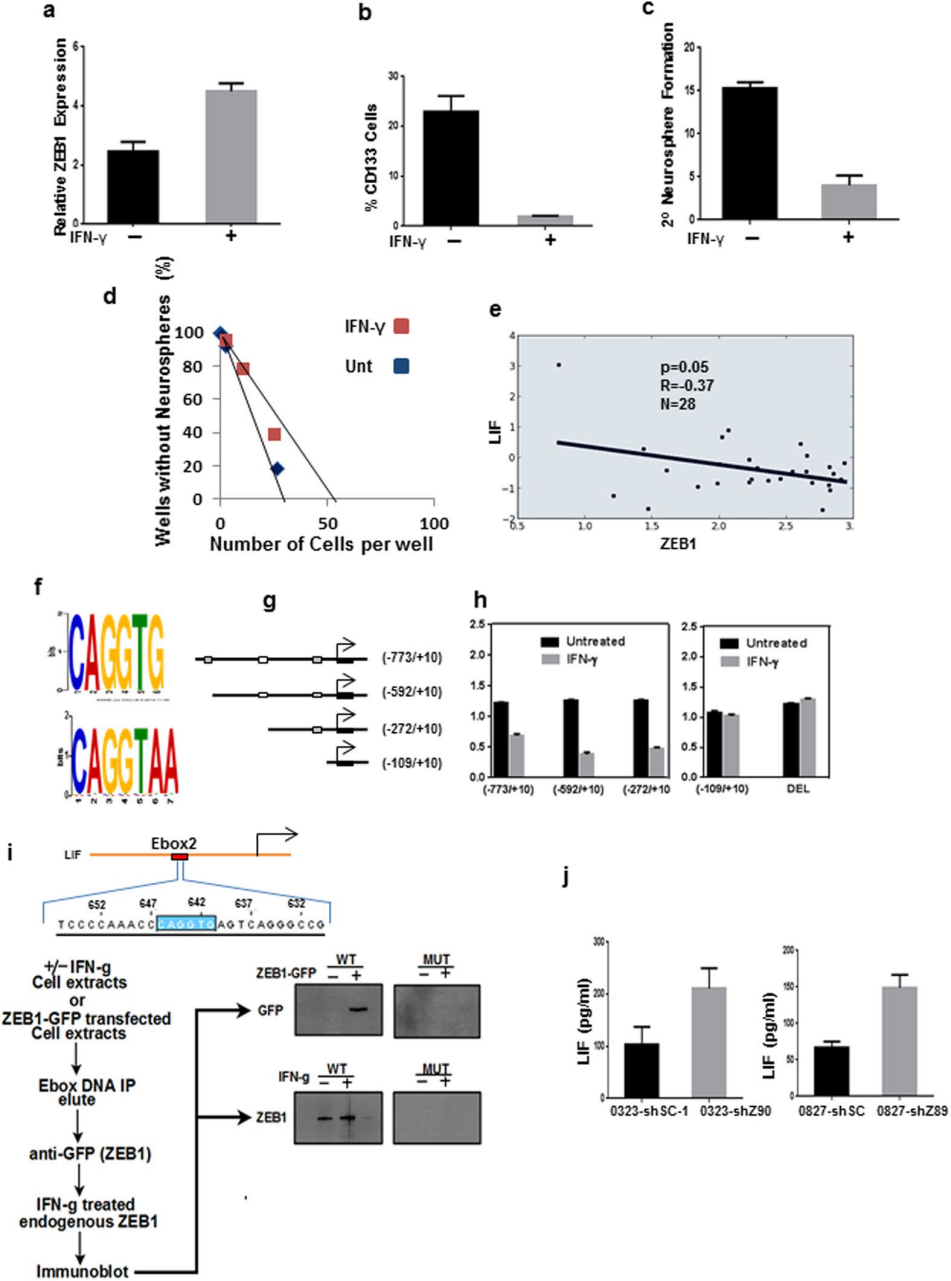
Effect of IFN-γ on LIF and ZEB1 activation in patient derived glioma cancer stem cells (GCSCs). (**a**) GCSCs were treated with IFN-γ for 3-days and ZEB1 expression levels were determined by qRT-PCR. (**b**) The percentage of CD133^+^ GCSCs in the presence and absence of IFN-γ were determined by flow cytometry. GCSCs were incubated with IFN-γ for 7-days. (**c**) The effects of IFN-γ on secondary neurosphere formation. (**d**) Limiting dilution sphere-forming assay indicated that cells not exposed to IFN-γ had increased self-renewal *in vitro*. (**e**) Spearman correlation between LIF and ZEB1 GBM patients (*n* = 28), rank correlation (R) and two-tailed significance is shown. (**f**) ZEB1 binding motifs within the LIF promoter (CAGGTG, ***P < 0.0001 and CAGGTA, ***P < 0.0001). (**g**) Schematic of LIF deletion constructs. (**h**) GCSCs transfected with LIF luciferase deletion constructs −773/+10, −592/+10, −272/+10, or −109/+10 or with ZEB1 binding sites deleted/mutated (DEL). The GCSCs were then incubated with IFN-γ to cause ZEB1 induction. (**i**) Oligonucleotide precipitation assay. Nuclear extracts from untreated GCSCs or GCSCs treated with IFN-γ were incubated with biotinylated double-stranded oligonucleotides corresponding to the putative ZEB1 binding motifs in the LIF promoter or a mutant version of that site (bottom western). Similarly, GFP-tagged ZEB1 was transiently transfected into GCSCs and the oligonucleotide precipitation assay was done (top western). (**j**) Determination of secreted LIF protein levels by ELISA after 72 hr treatment with IFN-γ of GCSCs 0323 (left) and 0827 (right). Error bars represent the mean of ± SEM of at least 3 experiments.

IFN-γ has been shown to have antagonistic effects on stem cell maintenance including decreased neurosphere formation, decreased self-renewal, and the promotion of differentiation^29, 30^. We sought to determine whether IFN-γ would cause induction of ZEB1, reinforcing the notion that ZEB1 activation leads to decreased stem cell activation. Exposure of GCSCs to IFN-γ resulted in a significant increase in ZEB1 induction compared to untreated GCSCs (Fig. 6a). Strikingly, in contrast to ZEB1 knockdown of expression by targeted shRNA which resulted in increased CD133 expression, induction of ZEB1 by IFN-γ resulted in decreased CD133 expression (Fig. 6b). IFN-γ also resulted in decreased secondary neurosphere formation (Fig. 6c). Similarly, IFN-γ treated GCSCs had decreased self-renewal capabilities compared to untreated GCSCs (Fig. 6d).

### *ZEB1* represses LIF expression in GCSCs

Through our analysis of GBMs for copy number we also looked at gene expression and found that a strong negative correlation was apparent between ZEB1 and LIF (Fig. 6e), a known regulator of stem cell self-renewal in gliomas^31^. Given that ZEB1 is also known to have repressive functions^3^ we explored a ZEB1 mediated suppression of LIF. Our attention was focused on a 2 kb region prior to the transcriptional start site of the LIF promoter. Analysis of the LIF promoter identified known ZEB1 E-box binding motifs (CAGGTG, *P* < 0.0001 and CAGGTA, *P* < 0.0001) within the LIF promoter region (Fig. 6f). We cloned the human LIF promoter into a luciferase reporter construct and made subsequent deletion constructs, which systematically eliminated the E-box binding sites to which ZEB1 could bind (Fig. 6g,h). We transfected our GCSCs with these constructs and treated our GCSCs with IFN-γ for ZEB1 induction. A suppressive effect was observed in all constructs with the exception of the −109/+10 region where ZEB1 binding sites were eliminated (Fig. 6h, left). Similarly, the deletion of the ZEB1 binding sites via the introduction of mutations in those sites also resulted in the rescue of LIF transcriptional activation (Fig. 6h right). A DNA pull-down of a biotinylated oligonucleotide of the ZEB1 binding site within the LIF promoter in GCSCs resulted in ZEB1 binding of exogenously expressed GFP tagged ZEB1 or to endogenously expressed ZEB1 after IFN-γ treatment (Fig. 6i). GCSCs targeted with shRNAs against ZEB1 (shZ89 or shZ90) resulted in increased LIF protein secretion compared to GCSCs targeted with non-targeting shRNA (shSC-1) as measured by ELISA (Fig. 6j) in normal stem cell media.

## Discussion

We studied the role of *ZEB1* loss in maintaining glioma cancer stem cell properties and its impact on patient survival in gliomas. Our data indicated that ZEB1 expression is lost in a significant number of glioma patients, and that the cause of ZEB1 loss is due in large part to heterozygous deletions in both GBMs and lower grade gliomas with frequent LOH in at least 20% of glioma patients. Despite *ZEB1* not being identified for copy number loss or mutations in TCGA analysis before, other cancers have shown the propensity for *ZEB1* to be deleted^10, 32^. Yet recent evidence and a re-examination of the data indicates that *ZEB1* does not carry deep or homozygous deletions which are identified through typical analyses using TCGA data and databases when looking at copy number, but are rather identified by examining raw copy number where heterozygous deletions can be more readily detected. The impact of ZEB1 copy number loss or decreased expression appears to be in the dysregulation of stemness. ZEB1 loss increases the expression of the stem cell promoting factor LIF which leads to a resistance to differentiation, and increased expression of the stem cell marker CD133. Our data also indicated that ZEB1 loss results in resistance to differentiation of GCSCs shown by increased cell proliferation under differentiation conditions and decreased expression of markers associated with differentiation. A further increase in the enrichment of the stem cell marker CD133 after knockdown of ZEB1 in patient derived GCSCs all indicate gain of function attributes associated with the loss of ZEB1^33^.

*ZEB1* loss and decreased expression is significantly associated with shorter patient survival, which is further exacerbated when we stratify patients who have ZEB1 loss and increased CD133. Furthermore, recent papers now reveal mutations in ZEB1^13, 14^ as did our own sequencing. Data portal sites^16, 17^ that utilize TCGA data also identify the heterozygous deletions consistent with our findings, however, the implications of ZEB1 heterozygous deletions have never been explored. Mutations and loss of heterozygosity may impact ZEB1 expression and play a role in the decreased survival noted. Furthermore, we postulate that in the absence of mutations or LOH that would affect ZEB1, there is a haploinsufficiency that results in a shortened survival for patients who have low expression of ZEB1. Finally, methylation may play a role in the decrease of ZEB1 expression. 

We and others have reported *ZEB1*’s role in the activation of GCSC invasion^3, 34^. It is not surprising, given the dual nature of ZEB1 to be both activator^34, 35^ and repressor^3, 36^, that the presence and absence of ZEB1 affects divergent GCSC functions. Furthermore, we have observed ZEB1 loss associated with cancer stemness and tumorigenicity^10^, however, other studies have shown the opposite^34^. One possible explanation for this discrepancy may be due to the heterogeneity of ZEB1 expression itself, in which two populations of GCSCs may exist, one with “high” ZEB1 expression and one with “low” ZEB1 expression. The low expressing population of ZEB1 GCSCs is consistent with an early less differentiated stem cell-like population and as the expression level of ZEB1 increases particularly in a homozygous wildtype ZEB1 population GCSCs become more differentiated. Interestingly, Siebzehnrubl, *et al.*, also indicated that of the GBM tumors they stained for ZEB1 by immunohistochemistry, 50% did not have ZEB1 expression. Another possible explanation may be that ZEB1 is heterogeneously expressed throughout a GBM or ZEB1 may be present in only in a fraction of the GCSC population. One could make the argument that ZEB1 may be highly expressed in cells that are involved in tumor invasion, accounting for a small fraction of the tumor cells which have highly invasive properties. WNT pathway activation was shown to increase the expression of ZEB1 as part of EMT. Intranuclear β catenin signal was noted in the infiltration zone. In addition, increased WNT activity increased CD133 expression^37^. The increase in CD133 as well as ZEB as a result of WNT pathway activation is counter to what we observed from ZEB1 expression decreasing CD133 expression in our GCSCs. These disparate findings suggest heterogeneous signals may affect “stemness”: ZEB1 loss can increase stemness whereas other signals such as WNT can increase ZEB along with CD133 expression. These findings highlight the importance of context in the expression of transcription factors such as ZEB1. It is not clear what the relationship of LOH of *ZEB1* is with respect to LOH of chromosome 10^38^. LOH of chromosome 10 occurs only in GBMs while LOH of *ZEB1* occurs in both GBM and lower grade gliomas. A large number of primary glioblastomas demonstrate a loss of the entire chromosome 10. In contrast, secondary glioblastomas demonstrate a partial or complete loss of chromosome 10q but no loss of 10p^39^. Given that ZEB1 loss occurs on 10p, it is likely that the occurrence of ZEB1 loss in lower grade gliomas and 10q loss in secondary glioblastomas occur independently. The loss of heterozygosity of chromosome 10q in secondary glioblastoma and the loss of heterozygosity at 10p in lower grade gliomas are distinct events. The LOH that occurs with 10p and the entire chromosome 10 in approximately half of primary glioblastoma may occur concurrently. The LOH of *ZEB1* only occurred in 21–29% in our studies. LOH of chromosome 10 was more frequent in primary GBM (47%) in one study^39^, suggesting that the LOH of chromosome 10 and LOH of ZEB1 are likely to result from distinct events. Our study did not specifically address this question. The fact that both lower grade gliomas and GBMs display similar rates of LOH of *ZEB1* despite the chromosomal loss in GBMs but not in lower grade gliomas suggests the independent occurrence of LOH at *ZEB1.* Furthermore, the prognostic significance of ZEB1 loss in both lower grade gliomas and glioblastomas may suggest that, lower grade gliomas that lose ZEB1 ultimately progress to GBMs, where the continued loss of ZEB1 progressively impacts overall prognosis. Indeed, loss of ZEB1 may continue to occur and impact prognosis in that more ZEB1 loss occurs in recurrent GBMs than in primary GBMs.

IFN-γ treatment of GCSCs like that of subventricular zone neural stem cells and neural progenitor cells, results in decreased self-renewal and neurosphere formation due to LIF suppression. ZEB1 has been shown to carry out both repressive and active functions in cancer. The likely decision to tend toward a more cancer stem cell-like phenotype rests on ZEB1 not binding the LIF promoter. Although IFN-γ has been suggested by some as a treatment for glioblastomas, our data suggest a more focused treatment strategy of IFN-γ targeting GCSCs may inhibit the propagation of this virulent subset of cells. These findings enable the actionable testing of therapies that increase intratumoral IFN-γ release, not only for immunologic ends but also to increase tumor differentiation and inhibit self-renewal. As IFN-γ activates ZEB1, which in turn suppresses LIF expression, ZEB1 expression can be queried as a surrogate measure for therapies that invoke tumor differentiation. These findings have the potential of impacting medical practice by demonstrating that *ZEB1* mutation, gene deletion and LOH impact patient survival. *ZEB1* deletion and expression can be used to prognosticate glioblastoma patients with greater accuracy. Deletion may be evaluated with other gene mutations of gliomas to further aid prognostication. Given the role of ZEB1 in stem cell maintenance, its expression can be used to query the stem cell properties of the tumor and assess the effect of differentiation therapies.

## Methods

### Tumor Samples

Patient brain tumor samples were classified as GBM based on the World Health Organization (WHO)^40^ criteria. All blood, brain tumors and patient derived GCSCs were approved by the Cedars-Sinai Medical Center institutional review board (IRB). Informed patient consent was obtained from all patients. All methods were carried out in accordance with the relevant guidelines of the IRB at Cedars-Sinai Medical Center.

### Archival Sources of specimens

DNA or RNA from GBM samples, GCSCs and patient blood were analyzed from TCGA and GEO datasets or samples were obtained from Cedars-Sinai Medical Center and extracted for whole genome copy number analysis, Sanger sequencing, real-time PCR and Exome sequencing. base calling, mutations and LOH identification were called using various software (dChip, MutsigCV, Phred, Chromosome Analysis Suite).

### Glioma Cancer Stem Cells (GCSCs)

GCSCs were isolated as previously described^24, 41^ and cultured in NBE media or differentiation media and infected with shRNAs as previously described^3^ and used in limiting dilution assays, neurosphere formation assays, ELISA, FACs or orthotopic xenograft mouse models. Some of these assays were also done with GCSCs exposed to IFN-ɣ for 3 or 7 days.

### Animals

Tumorigenicity was determined using GCSCs cultured in NBE media that were resuspended in HBSS and injected stereotactically into SCID mice. SCID mice were housed in a specific pathogen-free environment. Mice were sacrificed in accordance to NIH guidelines for the Care and Use of Laboratory Animals. All animal experiments were reviewed and approved by the Institutional Animal Care and Use Committee at Cedars-Sinai Medical Center.

### Methodology of Copy Number Loss of ZEB1

Our approach to the role of *ZEB1* copy number loss utilized the following methods assembling a variety of resources. For example, we obtained an initial 87 TCGA (The Cancer Genome Atlas) GBM patients using the Nexus biodiscovery application which contained curated copy number information for both primary and recurrent GBMs. We compared well characterized genes in GBM pathology for copy number alterations (e.g. *PTEN*, *EGFR*, *NF1*) as determined by the TCGA GBM Analysis Working Group, to the *ZEB1* gene in primary and recurrent cohorts. Our findings were supported by analyzing GBM patient samples from Cedars-Sinai Medical Center and 238 glioblastoma patient samples for *ZEB1* deletion downloaded from the TCGA data portal (https://tcga-data.nci.nih.gov/tcga/). We further confirmed *ZEB1* deletion through cBioportal and the COSMIC database (frozen Jan 2014). LOH and decreased ZEB1 expression was confirmed through GEO datasets and GBM patient samples (Cedars-Sinai Medical Center).

### Statistical Analysis

Data are expressed as mean ± s.e.m. Kaplan-Meier curves and *P* values were generated using Prism 6.0v. Two-tailed student’s *t*-test, were used. A *P* value of * <0.05 was considered significant.

## Electronic supplementary material

Supplementary information
