# Supplementary material for: ZEB1 regulates glioma stemness through LIF repression
Source: Sci Rep. 2017 Feb 28;7:69. doi: 10.1038/s41598-017-00106-x (PMC5427900; doi:10.1038/s41598-017-00106-x)
Supplement: Supplementary file 1 — Supplementary information [file 41598_2017_106_MOESM1_ESM.pdf]

Supplementary Information

ZEB1 regulates glioma stemness through LIF repression

Lincoln A. Edwards, Aiguo Li, Dror Berel, Mecca Madany, Nam-Ho Kim , Minzhi Liu, Mitch Hymowitz, Benjamin Uy, Rachel Jung, Minlin Xu, Keith L. Black, Altan Rentsendorj, Xuemo Fan, Wei Zhang, John S. Yu

Supplementary Methods

Clinical Samples and Cell Lines

We analyzed a total of 4,589 tumor genomes from which 22 were acquired from Cedars-Sinai Medical Center. Whole genome copy number was run by the UCLA Clinical Microarray Core for 6 GBMs and copy number was determined by Cytoscan® HD arrays. Due to the availability of the matched-normal blood plasma, tumor content, and amount of DNA we Sanger sequenced 7 tumor/normal pairs (Supplementary Fig. 2b). Exome sequencing was performed on 16 samples (Fig. 3a). 3 patient derived glioma cancer stem cell lines (GCSCs) were analyzed using Cytoscan® HD (Supplementary Fig. 2c). The fresh-frozen GBM samples were from primary tumors diagnosed as grade IV GBM tumors, and snap-frozen. All GBMs were assessed by a pathologist to confirm the diagnosis by H&E with no extensive signs of necrosis. Matching normal material was provided in the form of blood plasma. In addition, matching normal material was confirmed to be acquired from the same patient (n=7). Patient material was stored at -80°C. GBM samples were obtained from patients under IRB-approved protocols following written informed consent as were primary patient GCSCs designated 0827 and 0323, CSC-1-3,5,6,8.[26,43] All other tumor genomes analyzed from low grade gliomas and GBM cancer patients were provided by multiple institutions (listed below) by way of The Cancer Genome Atlas which was downloaded from the TCGA data portal (https://tcga-data.nci.nih.gov/tcga/) and the Gene Expression Omnibus (GEO) or Nexus Biodiscovery which was TCGA data.

Patient tumor material derived from:

-Harvard Medical School;
-Broad Institute
- Memorial Sloan Kettering Cancer Center;
- National Cancer Institute;
- University of North Carolina, Chapel Hill
-Kyushu University, Japan;
-Genentech;
- University of California at Los Angeles;
- Cedars-Sinai Medical Center

DNA and RNA extraction

DNA and RNA was extracted using QiaAmp DNA kit (Qiagen) for genomic DNA extraction and RNeasy RNA kit (Qiagen) for RNA extraction in accordance with the manufacturer's instructions.

Loss of Heterozygosity

Loss of Heterozygosity was performed in three different ways and analyzed by three different methods 1) Loss of Heterozygosity (LOH) found through clinical datasets in GEO. The resulting LOH data were analyzed with DNA-Chip Analyzer 2010.01 (www.dchip.org). The dChip program[1] allows for copy number as well as LOH analysis against a user defined reference or matched-pair samples. We normalized arrays using invariant set normalization. Signal intensities were used to infer copy number and LOH by the hidden Markov model (HMM). HMM inferred the probability of LOH based on LOH calls (from the paired tumor/normal samples) and this is displayed from blue (1) to white (0.5) to yellow (0). The dChipSNP was then used to visualize the LOH model for each sample and mapped to chromosome regions (Fig. 2a and Supplementary Fig 2a). 2) GCSCs from the National Cancer Institute and GBM patient tumors from Cedars-Sinai Medical Center were analyzed for LOH using Affymetrix Chromosome Analysis Suite (ChAS)-(Supplementary Fig. 2c) and/or Nexus Copy Number software for ZEB1 loss and determination of LOH after samples were run on Cytoscan® HD (Affymetrix, Cleveland, OH) at the UCLA Clinical Microarray Core. All arrays were performed using the Cytoscan® HD arrays and Cytoscan reagent kits in accordance with the manufacturer's instructions. 3) LOH was also determined through matching patient blood plasma and patient GBM tumor obtained from Cedars-Sinai Medical Center (*n*=7) using FinchTV (Supplementary Fig. 2b) for sequencing alignment after Sanger sequencing of exons in both patient blood plasma and GBM tumor.

Copy number analysis

Human DNA extracted from fresh-frozen GBM samples were hybridized to Cytoscan® HD arrays following the manufacturer's instructions. Signal intensities were processed to analyze for chromosomal gene copy number data .The raw, unsegmented copy number signals were used to analyze for significant copy number

alterations applying the CGARS[15] method. Significant amplifications were determined with the upper quantiles 0.25, 0.15, 0.1, and 0.05; deletions were computed in reference to the 0.25 lower quantile. The significance threshold was set at a q-value of 0.02 (Fig. 1a). Additionally, copy number was analyzed using snapCGH package for Rstudio (Supplementary Fig. d-f). Chromosomal gains and losses using snapCGH were defined by predicted values more than 0.75 times the interquartile range of the difference between observed and predicted for each region. The whisker boxplots of ZEB1 expression analysis associated with *ZEB1* genomic status were created using Prism v. 6.0. A two-tailed student t-test with unequal variation was used to measure the differences between groups (Fig. 1d, e and Supplementary Fig. 3a).

Dideoxy sequencing for validation of LOH

Initially exome sequencing was used to look for somatic mutations. Alternatively, dideoxynucleotide chain termination sequencing (Sanger sequencing) was performed to validate mutations and LOH. Coding sequences of *ZEB1* from GBM patient samples and patient blood were obtained using PCR and Sanger sequencing on genomic DNA. Primers (Supplementary Fig. 2b and Supplementary Table 2) were designed to cover the coding sequences plus at least 10 nucleotides in the intron region on both ends. Primer extension sequencing were performed by GENEWIZ, Inc. (South Plainfield, NJ) using Applied Biosystems BigDye version 3.1. Both forward and reverse strands were sequenced. The reactions were then run on Applied Biosystem's 3730xl DNA Analyzer. The sequencing data were analyzed with Lasergene SeqMan software and Finch TV (Geospiza, Inc) to detect any mutations compared to the genomic DNA reference sequence._

Data processing

The raw sequencing reads of human samples acquired from whole-genome, whole-exome were aligned to the respective human (NCBI37/hg19) reference genome. The data processing details could be found in the followingURL:(https://gforge.nci.nih.gov/docman/view.php/265/5004/Data_Preparation_and_Transfer_SOP.zip). Briefly, alignment was performed with the BWA aligner (version0.6.1-r104). The quality of the sequencing data was determined and genome sequencing data of human samples was analyzed for purity and ploidy. Somatic mutations were either already determined in retrospective analysis with additional enrichment to

customize the maf files or called at the UCLA CMC Mircoarray Core or called using MutSigCV and copy number alterations were determined as described above.

Reagents

The following antibodies were used: GFAP (Dako), TUJ1 (Covance), Nestin (Covance), Sox2 (Millipore), ZEB1 (Cell Signaling Technologies), ZEB1 (Santa Cruz Biotechnology), Actin (Sigma-Aldrich), CD133 (Miltenyi Biotech), Alexa-Fluor conjugated antibodies (Life Technologies), FITC (Sigma-Aldrich), HRP-secondaries IgG (Promega). IFN- (eBioscience) temozolomide was obtained through Cedars-Sinai Medical Center. ZEB1 constructs: GFP tagged (Origene), shRNA-ZEB1 (Origene), shRNA-nontargeting control (Origene). Temozolomide was obtained from either Cedars-Sinai Medical Center or the National Cancer Institute.

Immunohistochemistry

Immunohistochemistry was performed on paraffin TMAs as previously described.[2]

Immunostaining

GCSCs were plated onto chambered slides (Labtek) coated with poly-ornithine (Sigma-Aldrich) and Fibronectin (Sigma-Aldrich) with the appropriate media. Cells were fixed with 4% Formalin and permeabilized with 0.1% Triton-X-100 in PBS and blocked with 5% goat serum. GCSCs were incubated with primary antibodies overnight at 4 ºC and then washed in PBS before addition of the corresponding Alexa Fluor-conjugated secondary antibody (Life Technologies) for 1 hr at room temperature and mounted with mounting medium containing DAPI (Life Technologies) and analyzed by confocal microscopy.

Intracranial glioma cancer stem cell injection into SCID mice

Previously described in.[3,43] Briefly, To evaluate the tumorigenicity of GCSCs, stem cell media cultured GCSCs were resuspended in 2 μl of HBSS and injected stereotactically into adult SCID mice ~6-8 weeks of age. Tumor histology was evaluated by Hematoxylin and Eosin staining after removal of the mouse brain. Coordinates for

stereotactical injections into SCID mice were 3 mm distal to the midline, 2 mm anterior to the coronal suture, and 2.5 mm deep from the dura.

Western Blotting

Protein content was extracted from GCSCs in lysate form and protiein concentration was determined using a Bradford protein assay (Bio-Rad Laboratories). Equivalent amounts of protein were resolved by electrophoresis on premade 4%–15% gradient SDS–polyacrylamide gels (Bio-Rad Laboratories) and transferred to nitrocellulose membranes (Invitrogen). The membranes were incubated with either a ZEB1 antibody (Santa Cruz Biotechnology), or an Actin antibody (Sigma-Aldrich) was used to control for equal protein loading. The secondary antibodies were horseradish peroxidase–conjugated anti-mouse IgG and anti-rabbit IgG (Promega). Proteins were detected with the use of SuperSignal West Pico Chemiluminescent substrate (Pierce) and visualized after exposure to Kodak BioMax MS autoradiography films (Sigma).

GCSCs,Transient and Stable Infections

To generate GCSCs that stably express short hairpin RNAs (shRNAs) that target ZEB1, we co-transfected shRNA (Origene, Rockville MD), - that target ZEB1 into our 0827 or 0323 GCSCs, with a VSV-G expression plasmid (Clontech) into the GP2-293 packaging cell line (Clontech) according to the manufacturer's instructions. The resulting retroviral supernatants containing shRNA were used to infect 0827 and 0323. We used two shRNAs for targeting ZEB1, shRNA was not used together but were separately infected into either GCSCs. The shRNAs were designated as shZ89 or shZ90 for infection into GCSCs. Similarly, a non-targeting shRNAs shSC-1 was infected into either the 0827 or infected into 0323 GCSCs or GCSC-3. Forty-eight hours after infection, the medium was replaced with complete medium containing 0.1 ʒg/mL puromycin (Gibco) to select for shRNA-expressing GCSCs. Cells that were resistant to puromycin were characterized for ZEB1 expression by immunoblotting and subsequent cell proliferation using 5-ethynyl-2´-deoxyuridine (EdU) Click-IT assay (Life Technologies) using fluorescence activated cell sorting (FACs) analysis according to the manufacturer's instuctions. GCSCs were incubated with IFN- for either 3 days (200 ng/ml) or 7 days (100 ng/ml). GCSCs were incubated with temozolomide for 48 hr (25 µM). Transient transfection of ZEB1-GFP was done using X-treme gene HP DNA (Roche) according to the manufacturer's instructions.

Limiting Dilution Assay

Neural Basal A media (Invitrogen) supplemented with EGF and bFGF (R&D Systems) were used to culture primary patient derived GCSCs which were dissociated into single cells sorted for CD133 expression and plated onto 24 well plates with various seeding densities (4-100 cells/well). GCSCs were incubated at 37 эC at 5% CO_2 for 2 to 3 weeks. GCSCs were then quantified for neurosphere formation.

Fluorescence activated cell sorting/Magnetic activated cell sorting

GCSCs were washed with 1X PBS buffer 3 times and resuspended in 1X PBS. GCSCs were fixed in 4% formaldehyde for 15 min at room temperature. Cells were washed with 1X PBS buffer and were incubated in 0.1% Triton X-100 for 5 min, washed and then incubated with FcR Blocker (Mitenyi Biotech) followed by incubation with CD133 antibody conjugated to Phycoerythrin (PE) or although not shown an isotype control was also performed (Miltenyi Biotech), protected from light for 1 hazard ratio at room temperature. Cells were washed and analyzed on a FACscan flow cytometer (BD Biosciences). MACs sorting was performed as previously described.[3]

Oligonucleotide Precipitation Assays

Were performed as previously described[3] with the exception of the identification of the ZEB1 binding sites within the LIF promoter which were identified by Pscan (http://www.beaconlab.it/pscan,[4] and by comparing known E-box binding sites for ZEB1 and using the TOMTOM alogorithm.[5]

Luciferase Reporter Assays

To measure transcriptional activity of LIF, 0827 GCSCs (1 x 10^4 cells per transfection, three replicates per condition) were transiently transfected with one of several deletion LIF luciferase reporter plasmids (1 ug; Switchgear) with the use of X-treme gene HP DNA (Roche), seeded in six-well plates (1 x 10^4 cell per well), and incubated for 48 hrs. IFN- cytokine (200 ng/mL) was added to the cultures and the cells were incubated for 72 hrs. The cells were harvested and the luciferase activity was measured with the use of a GloMax 20/20 Luminometer (Promega, Madison, WT). These experiments were carried out in triplicate on three different

occasions. Note the original LIF luciferase reporter plasmid obtained from Switchgear was then subjected to site directed mutagenesis to obtain the appropriate deletion constructs.

Quantitative real time RT-PCR

Total RNAs from either GCSCs or GBM patient samples were isolated using RNeasy mini kit (Qiagen). Real-time PCR was performed using the IQ5 (Bio-rad) system according to the manufacturer's instructions. Template controls and samples were assayed in triplicate. The relative number of target transcripts was normalized to the number of human GAPDH transcripts found in the same sample. The relative quantitation of target gene expression was performed using the comparative cycle threshold (C_T) method. Human primers (Qiagen) used in the real time PCR were the following ZEB1, LIF, GAPDH,OLIG2, NOS2 and CD133.

ELISA

To determine quantitatively the total LIF secreted protein amount we used a LIF Human Quantikine ELISA kit (R&D systems) according to the manufacturer's specifications. The kit presents >95% cross-reactivity with human LIF relative to related molecules. 72 hrs after treatment with IFN- GCSC culture supernatants stably infected with either shRNAs targeting ZEB1 or non-targeting control were centrifuged to remove particles and concentrated with Amicon Ultra-4 Centrifugal Filters-10K (Millipore) to a final volume of 200 µl.

Differentiation of GCSCs

Was performed as previously described.[3]

References

1. Lin, M., Wei, L.J., Sellers, W.R., Lieberfarb, M., Wong, W.H., Li, C. dChipSNP: Significance Curve and Clustering of SNP-Array-Based Loss-of-Heterozygosity Data. *Bioinformatics* **20**, 1233-1240 (2004).
2. Spoelstra, N.S. *et al*. The Transcription Factor ZEB1 is Aberrantly Expressed in Aggressive Uterine Cancers. *Cancer Res*. **66**, 3893-3902 (2006).
3. Son, M.J., Woolard, K., Nam, D-H., Lee, J., Fine, H.A. SSEA-1 Is an Enrichment Marker for Tumor-Initiating Cells in Human Glioblastoma. *Cell Stem Cell* **4**, 440-452 (2009).
4. Zambelli, F., Pesole, G., Pavesi, G. Pscan: finding over-represented transcription factor binding site motifs in sequences from co-regulated or co-expressed genes. *Nucleic Acids Research* **37**, W247-W252 (2009).
5. Gupta, S., Stamatoyannopolous, J.A., Bailey, T., Noble, W.S. Quantifying similarity between motifs. *Genome Biology* **8**, R24-R32 (2007).

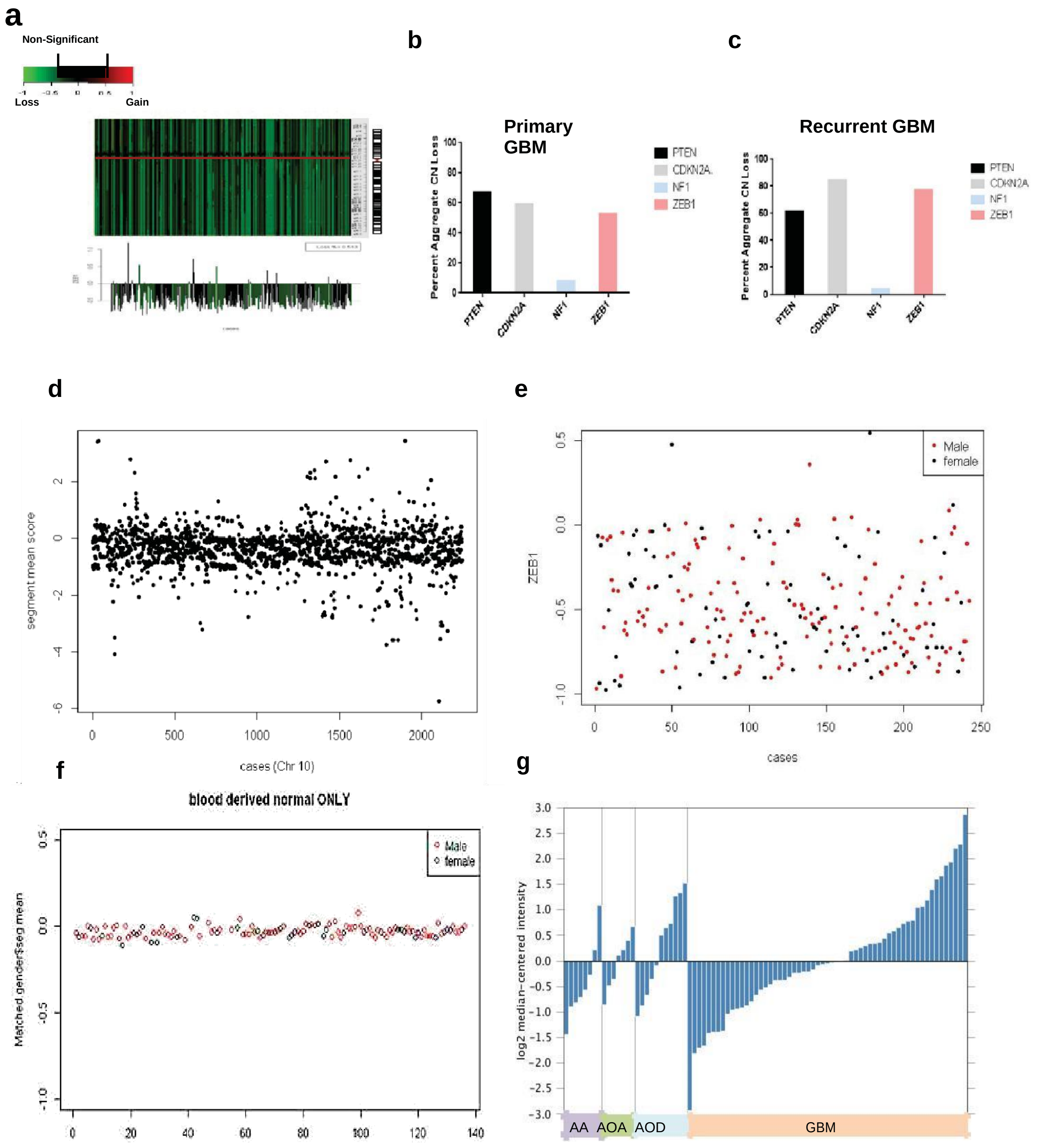

Supplementary Figure S1. *ZEB1* copy number variation.

(a) From a collection of 238 glioblastomas, copy number variation was visualized across chromosome 10 and specifically *ZEB1* was identified to have significant copy number loss (over 50% of cases-shown by orange line) indicative of ZEB1 deletion. Gene dosage profiles are indicated below copy number visualization. (b) Aggregate copy number loss from the most significant copy number loss genes from genome-wide analysis in Primary and (c) Recurrent glioblastomas. (d) aCGH ratio plots showing chromosome 10 from GBM tumor tissue compared to (e) specific ZEB1 region. (f) normal patient blood highlights loss of ZEB1. (g) ZEB1 expression measured by qPCR in Anaplastic Astrocytoma=AA *n*=8, Anaplastic Oligoastrocytoma=AOA *n*= 7, Anaplastic Oligodendroglioma=AOD *n*=11 and Glioblastoma=GBM *n*=59.

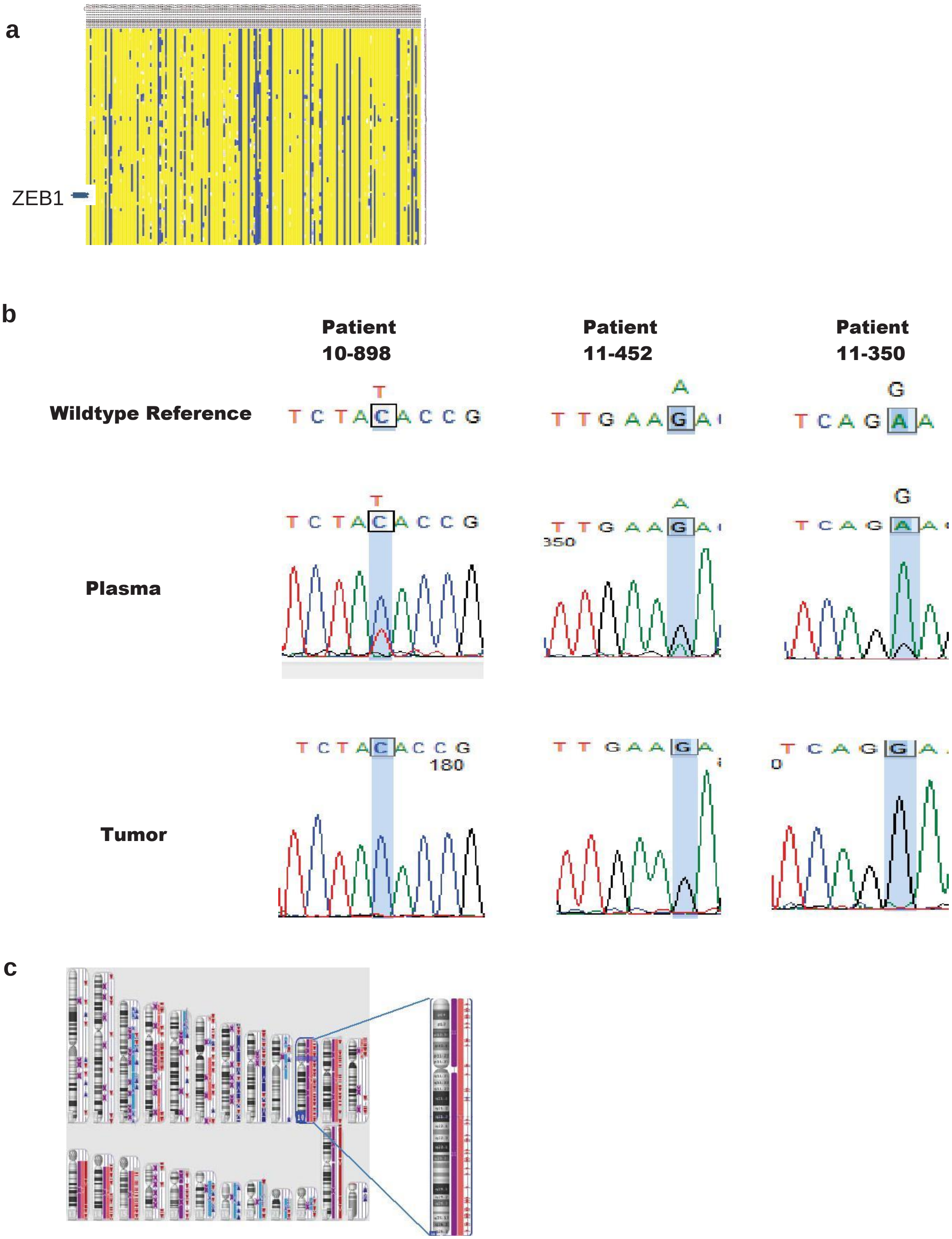

Supplementary Figure S2. Representative mutations and Loss of heterozygosity in *ZEB1.*

(a) HuSNP analysis to determine genome-wide LOH on 178 patient GBM samples. Approximate location of *ZEB1* gene across GBM patient samples from left to right is given by the arrow. Blue=LOH; yellow=retention. Threshold=0.46.(b) Sanger sequencing of GBM patients and matching blood plasma. (c) Karyotype analysis of patient derived GBM GSC 0827 indicating copy number changes loss=orange, copy number changes gain=light blue, LOH=purple, deletions= red arrows, amplifications=blue arrows. Chromosome 10 where *ZEB1* resides is blown up.

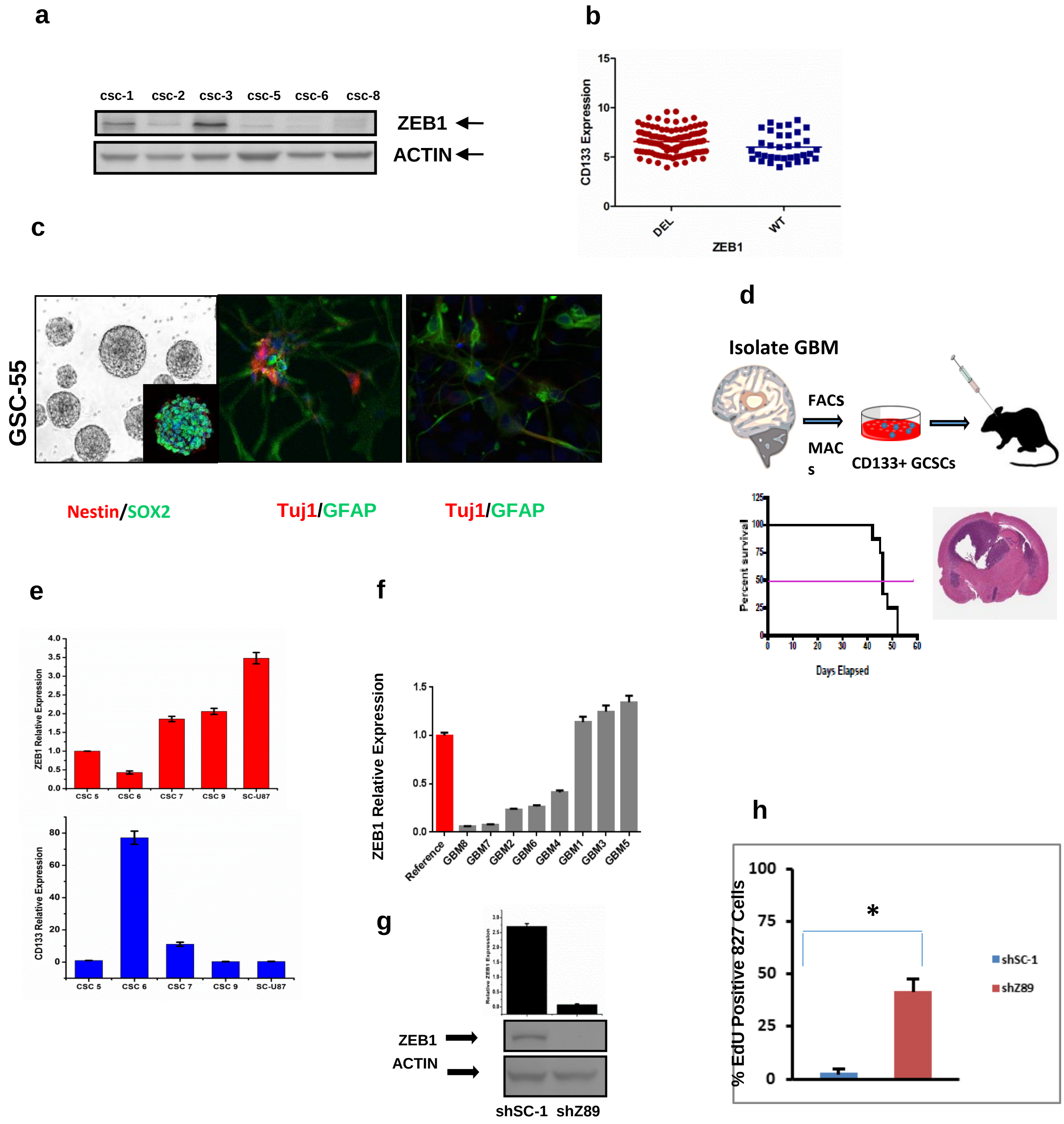

Supplementary Figure S3. Suppression of ZEB1 expression and its effect on CD133 and stemness.

(a) Protein determination by Western blot of ZEB1 loss in GSCs. (b) ZEB1 deletions (DEL), defined as copy number less than or equal to -0.5 (n=117); wildtype (WT) defined as copy number greater than or equal to zero (n=152) analyzed against CD133 expression (P=0.023). (c) Left, GCSCs forming neurospheres and expressing Nestin and Sox2. Middle, Right, GCSCs induced to differentiate expressing TUJ1 and GFAP. NBE =Neural Basal A media, WD = growth factor withdrawal, FBS = fetal bovine serum. (d) Validation of GCSC tumorigenicity. Top, schematic of GCSC isolation and subsequent intracranial injection. Bottom, Kaplan-Meier estimate of survival from injected GCSCs 0827 to form intracranial xenograft mouse (n=5) models, representative H&E of brain tumor formation. Mouse image provided by Soreu Loritzen. (e) Panel of primary GBMs from 8 patients were analyzed for ZEB1 expression. (f) RT-PCR of GSCs from Cedars-Sinai designated as cancer stem cells (CSCs) were quantified for ZEB1 expression and CD133 expression with an apparent inverse correlation in trend. (g) RT-PCR and Western blot confirmation of ZEB1 knockdown in 0827 GSCs with shRNA targeting ZEB1 (shZ89), a nontargeting shRNA (shSC-1) was included as a control. panel of primary GBMs from 8 patients were analyzed for ZEB1 expression. (h) Quantitation of 0827 GSCs transduced with either shSC-1 or shZ89 and cell proliferation of each was determined as measured by 5-ethynyl-2'-deoxyuridine (EdU) incorporation. *P<0.05. Error bars represent the mean ± SEM of at least 3 measurements

Supplementary Table 1: Primary and Recurrent Aggregate Copy Number gain and loss

		**Primary**		
Gene	CN Loss %	Homozygous Copy Loss %	CN Gain %	High Copy Gain %
PTEN	67·21		1·64	
NF1	8·2		21·31	
CCNE1	3·28		49·18	
CDK4	9·84		24·59	
EGFR			50·82	1·64
HYDIN	6·56		21·31	
LSAMP	8·2		21·31	
MDM4			31·15	1·64
MYC	3·28		22·95	1·64
PDGFRA			13·11	4·92
CDKN2A	18·03	40·98	13·11	
ZEB1	52·46	8·2	3·28	

Recurrent

Gene	CN Loss %	Homozygous Copy Loss %	CN Gain %	High Copy Gain %
PTEN	61·54			
NF1	3·85		23·08	
CCNE1	3·85		53·85	3·85
CDK4	19·23		15·38	
EGFR			26·92	23·08
HYDIN	19·23		11·54	
LSAMP	7·69		19·23	
MDM4			38·46	
MYC	7·69		19·23	3·85
PDGFRA	7·69		19·23	
CDKN2A	30·77	53·85	3·85	
ZEB1	76·92		3·85	

Supplementary Table 2: COSMIC copy number loss of ZEB1 in GBM patient tumors

CNV_id	Gene	Study	Sample	Copy Number	Type
322742	ZEB1	329	TCGA-06-0133-01	1	loss
323243	ZEB1	329	TCGA-06-0138-01	1	loss
323402	ZEB1	329	TCGA-06-0166-01	1	loss
323641	ZEB1	329	TCGA-06-0138-01	1	loss
323766	ZEB1	329	TCGA-06-0152-01	2	loss
324154	ZEB1	329	TCGA-06-0158-01	1	loss
324489	ZEB1	329	TCGA-06-0154-01	1	loss
324777	ZEB1	329	TCGA-06-0174-01	1	loss
325045	ZEB1	329	TCGA-06-0195-01	2	loss
325209	ZEB1	329	TCGA-06-0197-01	1	loss
325266	ZEB1	329	TCGA-06-0201-01	2	loss
325569	ZEB1	329	TCGA-06-0211-01	1	loss
325596	ZEB1	329	TCGA-06-0206-01	2	loss
325682	ZEB1	329	TCGA-06-0166-01	1	loss
325848	ZEB1	329	TCGA-06-0208-01	1	loss
325920	ZEB1	329	TCGA-06-0184-01	1	loss
326127	ZEB1	329	TCGA-06-0185-01	1	loss
326236	ZEB1	329	TCGA-06-0209-01	1	loss
326262	ZEB1	329	TCGA-06-0168-01	1	loss
326446	ZEB1	329	TCGA-06-0168-01	1	loss
326493	ZEB1	329	TCGA-06-0187-01	1	loss
326551	ZEB1	329	TCGA-06-0214-01	1	loss
326631	ZEB1	329	TCGA-06-0210-01	1	loss
326825	ZEB1	329	TCGA-06-0169-01	1	loss
326884	ZEB1	329	TCGA-06-0188-01	1	loss
327011	ZEB1	329	TCGA-06-0210-01	2	loss
327319	ZEB1	329	TCGA-06-0219-01	1	loss
327468	ZEB1	329	TCGA-06-0190-01	2	loss
327627	ZEB1	329	TCGA-06-0211-01	1	loss
327844	ZEB1	329	TCGA-06-0173-01	1	loss
328113	ZEB1	329	TCGA-06-0237-01	1	loss
328183	ZEB1	329	TCGA-06-0238-01	2	loss
328765	ZEB1	329	TCGA-06-0241-01	1	loss
328966	ZEB1	329	TCGA-06-0644-01	2	loss
329182	ZEB1	329	TCGA-06-0645-01	1	loss
329528	ZEB1	329	TCGA-06-0646-01	1	loss
329738	ZEB1	329	TCGA-06-0648-01	2	loss
333837	ZEB1	329	TCGA-08-0244-01	2	loss
334402	ZEB1	329	TCGA-08-0347-01	2	loss
334518	ZEB1	329	TCGA-08-0357-01	2	loss
334859	ZEB1	329	TCGA-08-0353-01	1	loss
334920	ZEB1	329	TCGA-08-0246-01	1	loss
335005	ZEB1	329	TCGA-08-0348-01	2	loss

335317	ZEB1	329	TCGA-08-0349-01	1	loss
335577	ZEB1	329	TCGA-08-0354-01	1	loss
312509	ZEB1	329	TCGA-02-0009-01	1	loss
312588	ZEB1	329	TCGA-02-0016-01	2	loss
313479	ZEB1	329	TCGA-02-0021-01	1	loss
314046	ZEB1	329	TCGA-02-0023-01	1	loss
314178	ZEB1	329	TCGA-02-0027-01	1	loss
314418	ZEB1	329	TCGA-02-0003-01	1	loss
314534	ZEB1	329	TCGA-02-0033-01	1	loss
314891	ZEB1	329	TCGA-02-0034-01	1	loss
315001	ZEB1	329	TCGA-02-0006-01	2	loss
315139	ZEB1	329	TCGA-02-0037-01	1	loss
315432	ZEB1	329	TCGA-02-0052-01	1	loss
315848	ZEB1	329	TCGA-02-0055-01	2	loss
315882	ZEB1	329	TCGA-02-0068-01	2	loss
315982	ZEB1	329	TCGA-02-0043-01	2	loss
316065	ZEB1	329	TCGA-02-0057-01	2	loss
316212	ZEB1	329	TCGA-02-0046-01	1	loss
316391	ZEB1	329	TCGA-02-0102-01	1	loss
316569	ZEB1	329	TCGA-02-0089-01	1	loss
317014	ZEB1	329	TCGA-02-0048-01	1	loss
317138	ZEB1	329	TCGA-02-0070-01	1	loss
317555	ZEB1	329	TCGA-02-0071-01	2	loss
317586	ZEB1	329	TCGA-02-0086-01	2	loss
317820	ZEB1	329	TCGA-02-0084-01	3	loss
317974	ZEB1	329	TCGA-02-0064-01	1	loss
318035	ZEB1	329	TCGA-02-0099-01	1	loss
318248	ZEB1	329	TCGA-02-0075-01	2	loss
318507	ZEB1	329	TCGA-02-0085-01	2	loss
318630	ZEB1	329	TCGA-06-0124-01	2	loss
318884	ZEB1	329	TCGA-06-0125-01	1	loss
319304	ZEB1	329	TCGA-06-0125-01	1	loss
319539	ZEB1	329	TCGA-02-0107-01	1	loss
319934	ZEB1	329	TCGA-02-0113-01	3	loss
320128	ZEB1	329	TCGA-06-0126-01	1	loss
320204	ZEB1	329	TCGA-06-0122-01	1	loss
320727	ZEB1	329	TCGA-06-0126-01	1	loss
320826	ZEB1	329	TCGA-06-0122-01	1	loss
320961	ZEB1	329	TCGA-02-0116-01	1	loss
321267	ZEB1	329	TCGA-06-0124-01	1	loss
321363	ZEB1	329	TCGA-06-0127-01	1	loss
321750	ZEB1	329	TCGA-02-0038-01	1	loss
321814	ZEB1	329	TCGA-06-0130-01	1	loss
321912	ZEB1	329	TCGA-06-0154-01	1	loss
321946	ZEB1	329	TCGA-06-0130-01	1	loss
322276	ZEB1	329	TCGA-06-0147-01	1	loss
322374	ZEB1	329	TCGA-06-0132-01	1	loss

322700	ZEB1	329	TCGA-06-0157-01	1	loss
335961	ZEB1	329	TCGA-08-0358-01	1	loss
336163	ZEB1	329	TCGA-08-0345-01	1	loss
336277	ZEB1	329	TCGA-08-0355-01	2	loss
336312	ZEB1	329	TCGA-08-0359-01	2	loss
336567	ZEB1	329	TCGA-08-0346-01	2	loss
336722	ZEB1	329	TCGA-08-0373-01	1	loss
336823	ZEB1	329	TCGA-08-0352-01	1	loss
336857	ZEB1	329	TCGA-08-0356-01	1	loss
337930	ZEB1	329	TCGA-12-0615-01	1	loss
338322	ZEB1	329	TCGA-12-0616-01	1	loss
338900	ZEB1	329	TCGA-08-0386-01	1	loss
338949	ZEB1	329	TCGA-12-0618-01	2	loss
339525	ZEB1	329	TCGA-08-0389-01	1	loss
339587	ZEB1	329	TCGA-12-0619-01	2	loss
340005	ZEB1	329	TCGA-12-0620-01	1	loss
340342	ZEB1	329	TCGA-08-0390-01	1	loss

Supplementary Table 3: ZEB1 Exon Sequencing Primers

Gene	Exon	Forward Primer	Reverse Primer
ZEB1	1	TTCGAGCCATCATTAAAATCAC	CTGGGTGGTTCAGACTCACA
	2	GGGTAGCTACTATTTGTCATTTTGG	TTGATTTCAAACTTTTCATCCAAT
	3	TCGGGAAGTTAAAATGTTTGTG	GGAAACGGACTAAATTCAGGA
	4	TTCTGCAGATTCAAGAACAATCA	TGCATGGTCATCATAGTGTTCC
	5	TGGAACATAGCATAGGGACTCA	TCAGGAATGACCAGATAACTCAAA
	6	TTCTGTCCCCACTATCACTATCC	GCCAAAAGAAATGCAAGGAG
	7-1	CCGCTTGTTTTAGGGAAATG	ATGGCCACCTTGTTGTATGG
	7-2	CGTCTCTTTCAGCATCACCA	CTCCTGGACAATCATCACACA
	7-3	AGCCATCAGTCTTCCTTTGG	ACTTTGCCTGGTTCAGGAGA
	7-4	TACTCAGCCTCCTCCACTCC	TGTCCTTTTGTGGCTCCTTT
	7-5	CCACCAATGGTTCCAGAAGT	AGTTGGCTCTACGGGACTGA
	8	GATCAGTGTGCTTGCTTTGG	AAAGAAAGAAAATTCTAAAAC
	9-1	TTGGGACCTGGAAATGTTTT	TCATCAGACCTTCAGTTTTTGC
	9-2	GAGAAGCGGAAGAACGTGAC	GCACACCCGGATTTATTTTG
	9*		TGAACAGGAATCACAGCATACA*

*The ZEB1-Ex9-2SeqR primer was used for the additional sequencing performed on exon 9

Supplementary Table 4. Whole genome copy number analysis

#Sample = 1904-EA-2662_7-JY_20131107

Chromosome Region	Event	Length	Cytoband	% CNV	% Heterozygous
chr1:4,536,073-4,645,033	LOH	108961	p36.32	0	0
chr1:9,507,979-10,232,941	LOH	724963	p36.22	1.73402192	5.494505495
chr1:20,613,564-21,009,170	LOH	395607	p36.12	0	0
chr1:21,949,462-22,576,673	LOH	627212	p36.12	9.1830341	5.223880597
chr1:23,266,426-23,786,152	LOH	519727	p36.12	0.24416712	3.47826087
chr1:25,466,582-26,106,742	LOH	640161	p36.11	26.1992314	1.086956522
chr1:26,746,567-27,366,915	LOH	620349	p36.11	0.25276135	1.5625
chr1:28,426,499-29,660,275	LOH	1233777	p35.3	0.29827132	4.294478528
chr1:31,863,044-32,992,895	LOH	1129852	p35.2 - p35.1	1.5415307	5.202312139
chr1:34,727,652-34,867,865	LOH	140214	p34.3	0	1.449275362
chr1:35,368,765-36,974,107	LOH	1605343	p34.3	4.4914417	3.90070922
chr1:39,603,277-40,083,845	LOH	480569	p34.3	0.52188244	1.086956522
chr1:45,082,373-45,523,437	LOH	441065	p34.1	0	1.449275362
chr1:48,715,637-49,190,114	LOH	474478	p33	1.90230506	3.846153846
chr1:52,397,263-52,890,520	LOH	493258	p32.3	1.00576373	2.247191011
chr1:55,518,775-55,792,064	LOH	273290	p32.3	0	2.325581395
chr1:56,412,426-56,756,970	LOH	344545	p32.2	0	0
chr1:58,190,707-58,401,063	LOH	210357	p32.2	0	1.136363636
chr1:59,051,639-59,290,504	LOH	238866	p32.1	0.60159504	1.265822785
chr1:59,951,980-60,702,023	LOH	750044	p32.1	2.05854864	3.603603604
chr1:61,302,280-61,738,268	LOH	435989	p31.3	0	4.787234043
chr1:62,438,073-62,546,195	CN Gain	108123	p31.3	13.2285751	0
chr1:62,438,073-62,546,195	LOH	108123	p31.3	13.2285751	0
chr1:63,835,277-64,088,270	LOH	252994	p31.3	0	1.694915254
chr1:64,088,270-64,099,209	CN Gain	10940	p31.3	0	20
chr1:64,171,058-64,483,736	LOH	312679	p31.3	0	0
chr1:64,927,424-65,163,371	LOH	235948	p31.3	0	1.315789474
chr1:65,563,921-65,869,161	LOH	305241	p31.3	0	4.225352113
chr1:67,374,656-67,609,196	LOH	234541	p31.3	0	2.857142857
chr1:67,775,075-67,789,572	CN Gain	14498	p31.3	0	
chr1:68,731,228-68,939,145	LOH	207918	p31.3 - p31.2	0	1.639344262
chr1:69,117,219-69,791,817	LOH	674599	p31.2 - p31.1	0	2.877697842
chr1:71,280,061-71,923,004	LOH	642944	p31.1	0.38214896	2.395209581
chr1:72,284,010-72,337,403	CN Gain	53394	p31.1	0	46.66666667
chr1:72,337,403-72,516,002	LOH	178600	p31.1	0.81019491	0
chr1:72,768,081-72,786,439	ligh Copy Gail	18359	p31.1	100	
chr1:72,786,439-72,812,172	CN Gain	25734	p31.1	99.7240897	0
chr1:75,273,920-76,607,860	LOH	1333941	p31.1	0	1.88172043
chr1:80,168,559-80,649,932	LOH	481374	p31.1	53.1756039	1.169590643

chr1:83,129,624-83,284,692	LOH	155069	p31.1	0	1.052631579
chr1:85,574,878-86,078,426	LOH	503549	p22.3	0	1.986754967
chr1:88,646,040-88,866,400	LOH	220361	p22.2	0	0
chr1:90,816,350-91,043,586	LOH	227237	p22.2	0	0
chr1:92,227,896-92,680,072	LOH	452177	p22.1	3.35820565	3.846153846
chr1:95,591,998-95,849,999	LOH	258002	p21.3	0	0
chr1:96,645,551-96,888,516	LOH	242966	p21.3	0	3.278688525
chr1:97,944,436-98,554,312	LOH	609877	p21.3	0	3.100775194
chr1:101,274,646-101,621,934	LOH	347289	p21.2	0	2.352941176
chr1:102,744,966-102,894,397	LOH	149432	p21.1	78.8865764	1.666666667
chr1:103,565,491-104,101,987	LOH	536497	p21.1	0	3.539823009
chr1:104,440,432-104,813,448	LOH	373017	p21.1	0.72436571	1.041666667
chr1:106,001,908-106,198,147	LOH	196240	p21.1	42.6785705	0
chr1:107,538,582-107,866,764	LOH	328183	p13.3	2.56900135	2.631578947
chr1:111,377,955-111,390,740	ozygous Copy	12786	p13.3	87.4540477	100
chr1:113,918,793-114,417,373	LOH	498581	p13.2	100	2.5
chr1:114,765,040-115,621,694	LOH	856655	p13.2	21.4788001	3.225806452
chr1:116,275,623-116,905,850	LOH	630228	p13.1	63.7871751	2.510460251
chr1:119,501,086-119,801,899	LOH	300814	p12	16.550814	0
chr1:120,551,399-120,630,807	CN Loss	79409	p12 - p11.2	100	
chr1:120,630,807-121,339,317	LOH	708511	p11.2	39.4923149	6.666666667
chr1:143,932,350-143,991,505	CN Loss	59156	q21.1	8.25120446	
chr1:149,995,654-151,524,230	LOH	1528577	q21.2 - q21.3	5.78760886	4.583333333
chr1:151,143,887-152,756,790	CN Gain	1612904	q21.3	19.9034288	34.15300546
chr1:151,524,230-152,756,790	llelic Imbalanc	1232561	q21.3	19.198822	39.36507937
chr1:152,771,308-158,324,427	llelic Imbalanc	5553120	q21.3 - q23.1	14.6805246	28.22445561
chr1:152,771,308-163,185,190	CN Gain	10413883	q21.3 - q23.3	15.5529705	26.17449664
chr1:158,324,427-158,729,334	LOH	404908	q23.1	2.00638665	1.234567901
chr1:158,729,334-163,185,190	llelic Imbalanc	4455857	q23.1 - q23.3	17.8712463	27.37556561
chr1:163,943,940-164,455,579	LOH	511640	q23.3	0	3.012048193
chr1:164,626,492-164,668,584	CN Gain	42093	q23.3	0	13.33333333
chr1:165,465,358-166,274,876	LOH	809519	q23.3 - q24.1	2.20550995	3.829787234
chr1:167,816,748-168,266,276	LOH	449529	q24.2	83.0818103	1.923076923
chr1:170,642,704-171,071,496	LOH	428793	q24.2 - q24.3	0	1.851851852
chr1:172,602,937-172,946,733	LOH	343797	q24.3 - q25.1	0.7702242	2.150537634
chr1:173,389,371-173,813,172	LOH	423802	q25.1	0	2.105263158
chr1:174,084,923-175,020,025	LOH	935103	q25.1	0.5835727	0.568181818
chr1:175,889,037-176,563,406	LOH	674370	q25.1 - q25.2	2.38326495	2.631578947
chr1:178,043,706-178,460,687	LOH	416982	q25.2	0	2.777777778
chr1:179,274,119-179,284,030	CN Gain	9912	q25.2	0	37.5
chr1:181,480,603-182,080,295	LOH	599693	q25.3	0.06670091	5.202312139
chr1:184,269,780-184,421,639	LOH	151860	q25.3	0	0
chr1:186,852,619-187,054,842	LOH	202224	q31.1	0	0
chr1:188,773,148-189,138,229	LOH	365082	q31.1	41.9290514	2.325581395
chr1:191,745,400-192,111,748	LOH	366349	q31.2	12.4258901	2.150537634
chr1:193,994,042-194,545,025	LOH	550984	q31.3	1.37227464	1.27388535
chr1:195,827,009-195,875,756	CN Loss	48748	q31.3	0	15

chr1:195,827,009-195,875,756	LOH	48748	q31.3	0	15
chr1:200,177,389-200,668,427	LOH	491039	q32.1	0.83394768	0.775193798
chr1:207,253,611-207,681,678	LOH	428068	q32.2	0.99633936	1.709401709
chr1:208,039,593-208,548,993	LOH	509401	q32.2	0	3.804347826
chr1:209,844,348-210,486,686	LOH	642339	q32.2	1.68727368	2.985074627
chr1:213,953,514-214,423,925	LOH	470412	q32.3	0	5.128205128
chr1:215,963,635-216,154,295	LOH	190661	q41	0	0
chr1:216,578,966-216,743,407	LOH	164442	q41	0	0
chr1:218,875,154-219,106,266	LOH	231113	q41	0	2.409638554
chr1:220,574,295-220,970,441	LOH	396147	q41	0.17316848	2.02020202
chr1:222,800,848-223,191,940	LOH	391093	q41	1.60550459	5.696202532
chr1:224,252,307-224,562,902	LOH	310596	q42.11	0.66742865	1.219512195
chr1:225,059,408-225,508,697	LOH	449290	q42.12	0.27265301	0.980392157
chr1:229,918,076-230,197,716	LOH	279641	q42.13	0.47739951	5.555555556
chr1:239,316,994-239,943,496	LOH	626503	q43	0	2.18579235
chr1:241,459,247-241,581,516	LOH	122270	q43	0	0
chr1:241,864,726-242,009,927	LOH	145202	q43	0	2.898550725
chr1:243,662,775-244,099,494	LOH	436720	q43 - q44	1.47715121	3.846153846
chr1:247,028,036-247,513,305	LOH	485270	q44	90.4395294	0.862068966
chr1:247,785,212-248,278,931	LOH	493720	q44	100	4.093567251
chr2:0-24,645	High Copy Gai	24646	p25.3	0	0
chr2:0-30,950	LOH	30951	p25.3	0	0
chr2:30,950-35,657	ozygous Copy	4708	p25.3	46.7813894	100
chr2:35,657-4,338,628	LOH	4302972	p25.3	15.7474684	2.785923754
chr2:4,338,628-4,831,706	llelic Imbalanc	493079	p25.3 - p25.2	4.42262685	36.25730994
chr2:4,338,628-4,831,706	CN Gain	493079	p25.3 - p25.2	4.42262685	36.25730994
chr2:4,831,706-4,914,619	High Copy Gai	82914	p25.2	0	33.92857143
chr2:4,914,619-5,575,733	CN Gain	661115	p25.2	26.861328	22.54901961
chr2:5,273,184-5,464,832	LOH	191649	p25.2	0.92095926	1.369863014
chr2:5,464,832-5,575,733	llelic Imbalanc	110902	p25.2	0	32.5
chr2:5,575,733-5,662,597	High Copy Gai	86865	p25.2	0	40.90909091
chr2:5,996,563-6,362,658	LOH	366096	p25.2	6.82910174	5.780346821
chr2:6,362,658-6,796,338	llelic Imbalanc	433681	p25.2	0.49829367	36.91588785
chr2:7,582,970-8,037,755	LOH	454786	p25.1	7.20736172	1.973684211
chr2:9,323,374-9,719,024	LOH	395651	p25.1	2.14204474	0
chr2:12,285,034-12,376,606	LOH	91573	p24.3	0.92277115	0
chr2:13,914,592-14,304,336	LOH	389745	p24.3	2.91704298	3.883495146
chr2:15,367,198-15,726,314	LOH	359117	p24.3	7.77019125	0
chr2:21,614,762-21,888,361	LOH	273600	p24.1	0	0
chr2:22,431,135-22,994,560	LOH	563426	p24.1	0.29693393	0.675675676
chr2:24,056,334-24,687,248	LOH	630915	p23.3	2.02309665	0.840336134
chr2:25,049,511-25,367,799	LOH	318289	p23.3	1.34940683	0
chr2:25,367,799-25,782,807	llelic Imbalanc	415009	p23.3	0.69131197	29.48717949
chr2:25,782,807-26,541,292	LOH	758486	p23.3	0.29572108	3.875968992
chr2:26,541,292-27,067,427	llelic Imbalanc	526136	p23.3	0	25.43859649
chr2:27,067,427-27,743,595	LOH	676169	p23.3	1.31653672	0.970873786
chr2:27,743,595-27,913,265	llelic Imbalanc	169671	p23.3 - p23.2	9.6169034	31.25

chr2:27,913,265-28,290,037	LOH	376773	p23.2	0	0
chr2:28,290,037-29,977,928	llelic Imbalanc	1687892	p23.2	0.65418916	31.68498168
chr2:5,662,597-52,746,735	CN Gain	47084139	p25.2 - p16.3	5.63104076	26.98109479
chr2:29,977,928-30,198,085	LOH	220158	p23.2 - p23.1	0.0490559	2.469135802
chr2:30,198,085-30,626,469	llelic Imbalanc	428385	p23.1	0	34
chr2:30,626,469-31,292,766	LOH	666298	p23.1	0	6.422018349
chr2:31,292,766-31,554,360	llelic Imbalanc	261595	p23.1	0	55.26315789
chr2:31,554,360-32,642,320	LOH	1087961	p23.1 - p22.3	0.96556859	2.209944751
chr2:32,642,320-33,059,285	llelic Imbalanc	416966	p22.3	100	25
chr2:33,059,285-33,331,337	LOH	272053	p22.3	99.492744	1.086956522
chr2:33,331,337-33,515,845	llelic Imbalanc	184509	p22.3	0	37.80487805
chr2:33,710,350-34,195,432	LOH	485083	p22.3	2.90033438	5.235602094
chr2:34,195,432-34,815,420	llelic Imbalanc	619989	p22.3	11.8787783	34.57943925
chr2:34,815,420-35,056,931	LOH	241512	p22.3	1.16143778	1.176470588
chr2:35,056,931-35,254,032	llelic Imbalanc	197102	p22.3	2.04717378	25.45454545
chr2:35,254,032-35,745,102	LOH	491071	p22.3	13.1645183	0.819672131
chr2:35,745,102-38,209,002	llelic Imbalanc	2463901	p22.3 - p22.2	8.20479727	27.94432548
chr2:38,209,002-38,331,917	LOH	122916	p22.2	0.1317984	1.428571429
chr2:38,331,917-39,154,670	llelic Imbalanc	822754	p22.2 - p22.1	3.3471771	40
chr2:39,154,670-39,730,206	LOH	575537	p22.1	0	4.511278195
chr2:39,730,206-42,310,596	llelic Imbalanc	2580391	p22.1 - p21	8.36191428	44.63768116
chr2:42,310,596-43,315,080	LOH	1004485	p21	0.09477503	0.373134328
chr2:43,315,080-44,392,963	llelic Imbalanc	1077884	p21	10.9585178	37.11656442
chr2:44,392,963-44,755,214	LOH	362252	p21	0.54271762	2.312138728
chr2:44,755,214-48,473,110	llelic Imbalanc	3717897	p21 - p16.3	0.62930754	33.01204819
chr2:48,473,110-48,845,877	LOH	372768	p16.3	0	0
chr2:48,845,877-50,623,740	llelic Imbalanc	1777864	p16.3	0.91643732	33.5483871
chr2:50,623,740-50,725,363	LOH	101624	p16.3	0	0
chr2:50,725,363-52,746,735	llelic Imbalanc	2021373	p16.3	3.3826035	35.85164835
chr2:52,746,735-52,785,847	ozygous Copy	39113	p16.3	96.1776437	
chr2:52,785,847-52,922,325	LOH	136479	p16.3 - p16.2	0	0
chr2:52,922,325-53,492,704	llelic Imbalanc	570380	p16.2	0.54069312	34.84848485
chr2:53,492,704-53,741,990	LOH	249287	p16.2	1.11077237	2.307692308
chr2:53,741,990-53,861,584	llelic Imbalanc	119595	p16.2	3.82544275	37.20930233
chr2:53,861,584-54,391,050	LOH	529467	p16.2	0	3.875968992
chr2:54,391,050-55,683,689	llelic Imbalanc	1292640	p16.2 - p16.1	1.75919185	27.20930233
chr2:55,683,689-56,212,870	LOH	529182	p16.1	5.35714623	3.875968992
chr2:56,212,870-57,042,397	llelic Imbalanc	829528	p16.1	4.14067294	44.15584416
chr2:57,042,397-57,267,181	LOH	224785	p16.1	0.56810093	0
chr2:57,267,181-59,803,136	llelic Imbalanc	2535956	p16.1	2.41853661	32.5170068
chr2:59,803,136-59,999,427	LOH	196292	p16.1	0	1.388888889
chr2:59,999,427-61,268,189	llelic Imbalanc	1268763	p16.1	1.01957656	37.1657754
chr2:52,785,847-68,943,752	CN Gain	16157906	p16.3 - p13.3	4.32315947	24.22852377
chr2:61,268,189-62,035,120	LOH	766932	p16.1 - p15	18.2008551	4.411764706
chr2:62,035,120-62,620,605	llelic Imbalanc	585486	p15	59.665918	26.41509434
chr2:62,620,605-64,372,887	LOH	1752283	p15 - p14	0.41192	2.760736196
chr2:64,372,887-64,624,288	llelic Imbalanc	251402	p14	0	25.24271845

chr2:64,624,288-64,984,597	LOH	360310	p14	0	2.173913043
chr2:64,984,597-66,137,005	llelic Imbalanc	1152409	p14	0.47387731	29.12087912
chr2:66,137,005-66,334,070	LOH	197066	p14	5.65092736	0
chr2:66,334,070-68,310,191	llelic Imbalanc	1976122	p14	0.72784005	31.05950653
chr2:68,310,191-68,868,854	LOH	558664	p14 - p13.3	0	4.605263158
chr2:68,868,854-68,943,752	llelic Imbalanc	74899	p13.3	0	52.38095238
chr2:69,417,091-69,991,802	LOH	574712	p13.3	0	0.67114094
chr2:70,613,291-70,764,587	LOH	151297	p13.3	1.07735829	0
chr2:71,384,408-71,681,501	LOH	297094	p13.3 - p13.2	0	0
chr2:72,166,361-73,131,001	LOH	964641	p13.2	0.07723088	2.923976608
chr2:73,566,475-74,123,813	LOH	557339	p13.1	15.047063	2.884615385
chr2:74,466,599-74,987,489	LOH	520891	p13.1	34.209718	1.818181818
chr2:79,299,170-79,539,813	LOH	240644	p12	7.46624668	2.597402597
chr2:79,761,051-79,934,342	LOH	173292	p12	0	0
chr2:79,982,754-80,004,189	CN Gain	21436	p12	0	26.66666667
chr2:80,071,215-80,092,055	CN Gain	20841	p12	100	47.05882353
chr2:81,934,293-82,569,328	LOH	635036	p12	0	0.952380952
chr2:83,108,958-83,356,634	LOH	247677	p12 - p11.2	49.6410633	1.666666667
chr2:86,036,364-87,633,240	LOH	1596877	p11.2	33.272715	1.408450704
chr2:98,118,169-98,163,648	CN Gain	45480	q11.2	90.505508	
chr2:98,625,278-98,999,148	LOH	373871	q11.2	8.28443042	2.469135802
chr2:99,859,053-100,639,764	LOH	780712	q11.2	0.27807985	5.617977528
chr2:100,923,723-101,156,428	LOH	232706	q11.2	0	0.961538462
chr2:101,820,860-102,143,518	LOH	322659	q11.2	58.3776011	0
chr2:103,273,084-103,521,309	LOH	248226	q12.1	0	0
chr2:103,792,091-104,042,782	LOH	250692	q12.1	0	2.040816327
chr2:104,024,817-104,042,782	CN Gain	17966	q12.1	0	0
chr2:105,795,211-106,073,389	LOH	278179	q12.1 - q12.2	0	0
chr2:108,944,526-109,525,834	LOH	581309	q12.3	0.70737028	2.150537634
chr2:114,548,199-114,867,264	LOH	319066	q14.1	0.27611929	4.761904762
chr2:117,233,086-117,723,329	LOH	490244	q14.1	0.40979677	3.623188406
chr2:117,982,514-118,515,231	LOH	532718	q14.1	0	2.197802198
chr2:121,917,320-122,566,455	LOH	649136	q14.2 - q14.3	0.08180117	5
chr2:123,410,184-123,768,134	LOH	357951	q14.3	1.70722168	5.035971223
chr2:125,491,634-126,292,571	LOH	800938	q14.3	1.57665334	5.357142857
chr2:129,424,480-129,785,520	LOH	361041	q14.3	2.7983049	2.586206897
chr2:129,912,692-130,316,679	LOH	403988	q21.1	78.1525148	0.680272109
chr2:135,485,214-136,967,585	LOH	1482372	q21.3 - q22.1	32.9321742	5.033557047
chr2:144,557,075-145,137,911	LOH	580837	q22.3	0	4.6875
chr2:145,303,692-145,639,168	LOH	335477	q22.3	1.30590564	1.111111111
chr2:147,792,312-148,287,637	LOH	495326	q22.3	1.03911573	4.672897196
chr2:151,997,539-152,279,971	LOH	282433	q23.3	0	0
chr2:152,487,666-153,377,972	LOH	890307	q23.3	0	2.109704641
chr2:162,998,864-164,436,027	LOH	1437164	q24.2 - q24.3	0	2.388059701
chr2:169,969,567-170,149,847	LOH	180281	q31.1	0	0
chr2:172,877,663-173,123,516	LOH	245854	q31.1	3.12056391	1.666666667
chr2:174,308,636-174,672,377	LOH	363742	q31.1	1.95936119	5.217391304

chr2:175,904,703-176,422,017	LOH	517315	q31.1	0	1.754385965
chr2:177,680,575-177,828,759	LOH	148185	q31.1	2.2384333	2.702702703
chr2:178,240,605-178,552,731	LOH	312127	q31.2	0.12494954	1.149425287
chr2:179,245,217-179,651,896	LOH	406680	q31.2	0.25425458	3.296703297
chr2:181,824,940-182,023,137	LOH	198198	q31.3	13.866002	0
chr2:182,437,120-182,859,730	LOH	422611	q31.3	1.17413218	2.150537634
chr2:184,074,860-184,091,579	CN Loss	16720	q32.1	38.5070877	0
chr2:184,074,860-184,091,579	LOH	16720	q32.1	38.5070877	0
chr2:184,932,839-185,369,255	LOH	436417	q32.1	13.318256	0.884955752
chr2:188,868,241-189,758,707	LOH	890467	q32.1 - q32.2	1.60724834	1.639344262
chr2:190,498,014-190,763,721	LOH	265708	q32.2	0	0
chr2:192,077,359-192,344,732	LOH	267374	q32.3	0	1.315789474
chr2:193,317,648-194,389,675	LOH	1072028	q32.3	0.12882138	3.317535545
chr2:196,739,058-197,083,494	LOH	344437	q32.3	0	0
chr2:198,534,888-198,960,031	LOH	425144	q33.1	0	3.03030303
chr2:201,741,266-202,246,213	LOH	504948	q33.1	1.13932749	1.369863014
chr2:206,933,447-207,311,261	LOH	377815	q33.3	0	1.369863014
chr2:210,379,846-210,632,771	LOH	252926	q34	0	0
chr2:214,617,633-214,802,505	LOH	184873	q34	0	1.587301587
chr2:214,896,928-215,575,906	LOH	678979	q34 - q35	0	4.807692308
chr2:215,970,324-216,363,922	LOH	393599	q35	0	2.830188679
chr2:219,249,014-219,721,119	LOH	472106	q35	0.03791529	4.705882353
chr2:226,362,455-226,693,204	LOH	330750	q36.3	0	1.333333333
chr2:226,796,136-227,129,195	LOH	333060	q36.3	2.13325567	0
chr2:227,464,937-227,763,182	LOH	298246	q36.3	0.48383041	0
chr2:239,216,214-239,484,900	LOH	268687	q37.3	8.77529905	0
chr2:239,860,565-240,193,305	LOH	332741	q37.3	0.16198834	4.929577465
chr3:0-652,018	llelic Imbalanc	652019	p26.3	11.1070553	20.61281337
chr3:0-652,018	CN Loss	652019	p26.3	11.1070553	20.61281337
chr3:652,018-832,441	LOH	180424	p26.3	6.29132649	7.407407407
chr3:2,157,399-2,174,335	CN Gain	16937	p26.3	0	25
chr3:2,275,050-2,576,258	LOH	301209	p26.3	0.15371438	6.306306306
chr3:2,687,441-2,801,855	LOH	114415	p26.3 - p26.2	0	1.886792453
chr3:6,239,436-6,364,744	LOH	125309	p26.1	7.84307466	0
chr3:7,212,515-7,372,370	LOH	159856	p26.1	1.10913015	1.639344262
chr3:8,649,551-9,074,839	LOH	425289	p26.1 - p25.3	10.3146574	2.958579882
chr3:11,272,868-11,618,575	LOH	345708	p25.3	1.3187468	0
chr3:12,499,216-12,861,525	LOH	362310	p25.2	53.4218029	0.751879699
chr3:16,873,845-17,137,083	LOH	263239	p24.3	6.38547626	0
chr3:17,875,244-18,400,595	LOH	525352	p24.3	0	4.098360656
chr3:22,885,014-23,149,394	LOH	264381	p24.3	1.42559952	1.369863014
chr3:25,256,219-25,480,330	LOH	224112	p24.2	0	1.063829787
chr3:25,928,554-26,551,304	LOH	622751	p24.2 - p24.1	2.79566439	3.428571429
chr3:27,031,142-27,444,003	LOH	412862	p24.1	0	0.763358779
chr3:30,830,630-31,037,225	LOH	206596	p24.1 - p23	8.43195624	0
chr3:31,351,895-32,158,324	LOH	806430	p23 - p22.3	0.77700579	3.333333333
chr3:32,463,478-32,877,622	LOH	414145	p22.3	0.22407665	1.030927835

chr3:33,255,718-33,928,286	LOH	672569	p22.3	0	3.225806452
chr3:34,411,911-34,989,555	LOH	577645	p22.3	0	2.48447205
chr3:35,986,992-36,348,592	LOH	361601	p22.3	100	2.127659574
chr3:36,963,338-37,281,531	LOH	318194	p22.2	0	1.408450704
chr3:40,004,487-40,596,557	LOH	592071	p22.1	0	2.836879433
chr3:41,680,774-42,094,150	LOH	413377	p22.1	12.678288	1.298701299
chr3:44,232,477-45,036,161	LOH	803685	p21.31	0	0
chr3:47,373,860-47,663,043	CN Gain	289184	p21.31	0.93159003	41.37931034
chr3:47,868,779-48,394,644	LOH	525866	p21.31	0.3057819	2.564102564
chr3:56,521,232-56,797,809	LOH	276578	p14.3	5.21337638	0
chr3:58,590,179-59,118,749	LOH	528571	p14.3 - p14.2	0.26354125	2.362204724
chr3:59,670,461-59,784,099	LOH	113639	p14.2	0.84566782	3.658536585
chr3:62,659,880-62,863,148	LOH	203269	p14.2	16.5308853	0.909090909
chr3:63,752,316-64,111,073	LOH	358758	p14.1	0	1.149425287
chr3:67,271,531-67,624,355	LOH	352825	p14.1	0.46992268	3.048780488
chr3:78,507,695-79,264,589	LOH	756895	p12.3	0.49610646	6.818181818
chr3:81,662,324-82,048,946	LOH	386623	p12.2	0	5.660377358
chr3:84,480,497-84,868,748	LOH	388252	p12.1	4.73379335	0
chr3:85,387,320-85,659,267	LOH	271948	p12.1	0	0
chr3:86,201,078-91,000,000	LOH	4798923	p12.1 - q11.1	5.78773733	1.763485477
chr3:93,536,054-95,375,846	LOH	1839793	q11.1 - q11.2	0.38379339	2.314814815
chr3:96,548,071-97,362,218	LOH	814148	q11.2	0	2.325581395
chr3:97,962,606-98,150,158	LOH	187553	q11.2	5.99460416	0
chr3:98,191,248-98,619,010	LOH	427763	q11.2 - q12.1	1.44052066	3.225806452
chr3:107,296,441-107,556,642	LOH	260202	q13.12	0.94965046	1.666666667
chr3:109,954,549-110,366,662	LOH	412114	q13.13	0	0.724637681
chr3:111,292,541-111,593,760	LOH	301220	q13.13 - q13.2	0	0
chr3:112,164,430-112,338,567	LOH	174138	q13.2	0	1.923076923
chr3:112,338,567-112,814,368	llelic Imbalanc	475802	q13.2	0	33.33333333
chr3:112,814,368-113,008,995	LOH	194628	q13.2	0	1.369863014
chr3:112,112,174-114,740,115	CN Gain	2627942	q13.2 - q13.31	0.57075102	25.44378698
chr3:113,008,995-114,740,115	llelic Imbalanc	1731121	q13.2 - q13.31	0.79942465	29.52853598
chr3:114,740,115-115,196,107	LOH	455993	q13.31	0.12061615	2.830188679
chr3:115,353,454-115,825,587	llelic Imbalanc	472134	q13.31	0	27.77777778
chr3:115,353,454-115,825,587	CN Gain	472134	q13.31	0	27.77777778
chr3:117,998,945-118,905,250	LOH	906306	q13.32	8.87052372	1.287553648
chr3:121,781,557-122,022,312	LOH	240756	q13.33 - q21.1	1.15054724	3.03030303
chr3:122,291,190-122,642,998	LOH	351809	q21.1	0	1.204819277
chr3:129,747,238-130,283,086	LOH	535849	q22.1	36.0490288	2.325581395
chr3:133,711,122-134,022,985	LOH	311864	q22.2	0	0
chr3:134,414,262-134,625,030	LOH	210769	q22.2	0	1.111111111
chr3:140,450,766-141,021,711	LOH	570946	q23	0.47920553	5.882352941
chr3:143,158,771-143,306,093	LOH	147323	q24	0.96048112	1.666666667
chr3:144,394,839-144,638,238	LOH	243400	q24	0	0
chr3:144,951,156-145,162,179	LOH	211024	q24	5.78941632	1.449275362
chr3:145,448,796-145,655,262	LOH	206467	q24	14.1669815	0
chr3:147,548,484-147,607,534	CN Gain	59051	q24	1.03471634	64.28571429

chr3:147,791,995-148,189,788	LOH	397794	q24	0.75013889	0
chr3:149,127,343-149,151,338	llelic Imbalanc	23996	q25.1	0	14.28571429
chr3:149,127,343-149,151,338	CN Loss	23996	q25.1	0	14.28571429
chr3:151,082,616-151,279,619	LOH	197004	q25.1	0	2.702702703
chr3:154,095,094-154,827,776	LOH	732683	q25.2	0.04490352	2.424242424
chr3:155,102,372-155,408,534	LOH	306163	q25.31	0	2.702702703
chr3:156,832,994-157,053,914	LOH	220921	25.31 - q25.3	0	1.639344262
chr3:157,681,423-158,627,406	LOH	945984	q25.32	0.357406	0.869565217
chr3:158,921,595-159,501,027	LOH	579433	25.32 - q25.3	0.12391445	2
chr3:159,624,144-159,846,364	LOH	222221	q25.33	0	0
chr3:160,314,898-160,595,361	LOH	280464	q25.33	1.90577723	0
chr3:161,742,021-162,321,203	LOH	579183	q26.1	17.5849733	8.024691358
chr3:165,470,249-165,938,495	LOH	468247	q26.1	0.22061053	3.03030303
chr3:167,902,359-168,214,124	LOH	311766	q26.2	0	2.857142857
chr3:169,355,639-169,711,922	LOH	356284	q26.2	0	1.136363636
chr3:170,649,751-171,091,481	LOH	441731	q26.2 - q26.31	0	1.526717557
chr3:172,689,592-173,131,354	LOH	441763	q26.31	0	5.769230769
chr3:174,226,986-174,485,705	LOH	258720	q26.31	0	0
chr3:174,532,767-174,788,846	LOH	256080	q26.31	0	2.5
chr3:175,187,273-175,426,321	LOH	239049	q26.31	16.5234597	0
chr3:177,592,331-178,105,830	LOH	513500	q26.32	0.20993225	2.752293578
chr3:180,348,354-181,042,159	LOH	693806	q26.33	0	4.615384615
chr3:181,639,571-182,227,276	LOH	587706	q26.33	0	3.03030303
chr3:183,410,094-183,792,787	LOH	382694	q27.1	0.71650122	0
chr3:188,695,254-188,955,859	LOH	260606	q28	0.34880375	0
chr3:191,063,766-191,322,206	LOH	258441	q28	5.82262808	3.921568627
chr3:193,268,313-193,487,829	LOH	219517	q29	0	1.351351351
chr4:1,589,051-2,343,313	LOH	754263	p16.3	0.43419926	3.731343284
chr4:2,945,971-3,505,148	LOH	559178	p16.3	4.78542572	0
chr4:4,348,249-5,032,105	LOH	683857	p16.3 - p16.2	0.07413842	3.459119497
chr4:5,809,898-6,750,539	LOH	940642	p16.2 - p16.1	1.4642143	2.356902357
chr4:11,628,305-11,767,446	LOH	139142	p15.33	100	1.612903226
chr4:14,393,452-14,562,817	LOH	169366	p15.33	0	1.538461538
chr4:15,696,885-15,985,028	LOH	288144	p15.32	3.0370337	3.703703704
chr4:16,790,756-17,058,420	LOH	267665	p15.32	1.4944109	1.369863014
chr4:19,424,563-19,594,021	CN Gain	169459	p15.31	0	64.17910448
chr4:20,629,313-20,985,129	LOH	355817	p15.31	0	1.754385965
chr4:21,000,773-21,034,589	CN Gain	33817	p15.31	0	54.54545455
chr4:21,428,679-21,516,982	CN Gain	88304	p15.2	21.3209064	25.45454545
chr4:22,859,678-23,259,049	LOH	399372	p15.2	0.40488668	1.25
chr4:23,582,895-23,787,491	LOH	204597	p15.2	0	1.369863014
chr4:31,217,627-31,319,381	CN Gain	101755	p15.1	0.64076105	55.55555556
chr4:32,311,836-32,704,141	LOH	392306	p15.1	1.64565835	1.754385965
chr4:32,996,144-33,581,385	LOH	585242	p15.1	1.54893454	4.109589041
chr4:34,354,208-35,034,363	LOH	680156	p15.1	41.3238159	3.333333333
chr4:36,299,097-36,421,633	LOH	122537	p14	0	0
chr4:44,381,583-44,863,754	LOH	482172	p13 - p12	0	0

chr4:45,837,575-46,391,491	LOH	553917	p12	0.48021722	0.740740741
chr4:53,298,358-53,638,415	LOH	340058	q12	0	0
chr4:53,872,714-54,172,366	LOH	299653	q12	3.5838239	0
chr4:55,364,151-55,598,003	LOH	233853	q12	0	1.388888889
chr4:58,241,692-58,544,286	LOH	302595	q12	1.76903706	1.785714286
chr4:61,005,416-62,862,582	llelic Imbalanc	1857167	q13.1	1.17943146	35.54216867
chr4:62,862,582-63,660,426	LOH	797845	q13.1	0	5.633802817
chr4:63,660,426-64,408,096	llelic Imbalanc	747671	q13.1	9.01962096	35.71428571
chr4:64,408,096-64,743,118	LOH	335023	q13.1	14.9002155	4.819277108
chr4:64,743,118-65,111,522	llelic Imbalanc	368405	q13.1	0.24429702	41.25
chr4:65,111,522-65,240,787	CN Loss	129266	q13.1	0	30.3030303
chr4:65,240,787-65,322,553	llelic Imbalanc	81767	q13.1	0	56.75675676
chr4:65,322,553-65,553,186	CN Loss	230634	q13.1	0	25
chr4:65,376,608-65,642,107	LOH	265500	q13.1	0	5.714285714
chr4:65,642,107-66,307,742	CN Loss	665636	q13.1	3.18072217	42.7184466
chr4:66,382,561-66,522,625	CN Loss	140065	q13.1	0	13.15789474
chr4:66,382,561-66,522,625	LOH	140065	q13.1	0	13.15789474
chr4:66,522,625-67,116,067	llelic Imbalanc	593443	q13.1 - q13.2	11.1977245	25.98870056
chr4:66,632,969-67,116,067	CN Loss	483099	q13.2	5.00478164	24.65753425
chr4:67,116,067-67,611,470	LOH	495404	q13.2	0	7.874015748
chr4:67,611,470-67,754,995	llelic Imbalanc	143526	q13.2	0	32.0754717
chr4:67,221,646-68,190,705	CN Loss	969060	q13.2	0	15.78947368
chr4:67,754,995-67,936,107	LOH	181113	q13.2	0	0
chr4:67,936,107-68,190,705	llelic Imbalanc	254599	q13.2	0	31.25
chr4:68,338,381-68,649,068	CN Loss	310688	q13.2	1.08791163	22.78481013
chr4:68,338,381-68,987,379	llelic Imbalanc	648999	q13.2	12.8776976	30.17751479
chr4:68,801,222-69,366,734	CN Loss	565513	q13.2	17.0196212	12.24489796
chr4:68,987,379-69,366,734	LOH	379356	q13.2	5.24416444	0
chr4:69,366,734-69,536,787	CN Gain	170054	q13.2	100	0
chr4:69,536,787-70,325,218	llelic Imbalanc	788432	q13.2	45.4166566	36.50793651
chr4:69,536,787-70,325,218	CN Loss	788432	q13.2	45.4166566	36.50793651
chr4:70,509,526-70,913,014	llelic Imbalanc	403489	q13.3	0	48.42105263
chr4:70,509,526-71,534,848	CN Loss	1025323	q13.3	11.1387447	28.802589
chr4:70,913,014-71,215,721	LOH	302708	q13.3	2.62696271	4.672897196
chr4:71,215,721-71,534,848	llelic Imbalanc	319128	q13.3	33.2958352	35.51401869
chr4:71,534,848-72,091,859	LOH	557012	q13.3	1.81576306	7.272727273
chr4:71,857,998-72,091,859	CN Loss	233862	q13.3	4.32479122	7.547169811
chr4:72,091,859-72,397,797	llelic Imbalanc	305939	q13.3	0	25.97402597
chr4:72,397,797-72,464,927	CN Loss	67131	q13.3	0	9.523809524
chr4:72,397,797-72,464,927	LOH	67131	q13.3	0	9.523809524
chr4:72,464,927-72,924,953	llelic Imbalanc	460027	q13.3	0	29.20353982
chr4:72,924,953-72,972,205	CN Loss	47253	q13.3	0	6.25
chr4:72,924,953-72,972,205	LOH	47253	q13.3	0	6.25
chr4:72,972,205-73,102,739	llelic Imbalanc	130535	q13.3	2.43461474	50
chr4:73,102,739-73,408,164	CN Loss	305426	q13.3	18.6582631	23.88059701
chr4:73,408,164-73,646,931	LOH	238768	q13.3	22.0202122	3.488372093
chr4:73,646,931-74,034,326	llelic Imbalanc	387396	q13.3	0	43.82022472

chr4:73,445,437-74,514,951	CN Loss	1069515	q13.3	2.16397354	23.828125
chr4:74,514,951-74,726,736	llelic Imbalanc	211786	q13.3	0.66104776	32.20338983
chr4:74,576,467-74,726,736	CN Loss	150270	q13.3	0	41.02564103
chr4:74,890,723-75,088,571	CN Loss	197849	q13.3	4.15622094	15
chr4:75,088,571-75,271,709	LOH	183139	q13.3	0	2.040816327
chr4:75,158,877-75,339,048	CN Loss	180172	q13.3	0	11.11111111
chr4:75,271,709-75,339,048	llelic Imbalanc	67340	q13.3	0	55.55555556
chr4:75,524,063-75,606,566	llelic Imbalanc	82504	q13.3	0	40.625
chr4:75,524,063-75,606,566	CN Loss	82504	q13.3	0	40.625
chr4:75,606,566-75,800,256	LOH	193691	q13.3	0	7.142857143
chr4:75,800,256-76,084,380	llelic Imbalanc	284125	q13.3	0	25.64102564
chr4:75,916,875-76,422,552	CN Loss	505678	q13.3 - q21.1	0	7.006369427
chr4:76,084,380-76,422,552	LOH	338173	q13.3 - q21.1	0	1.923076923
chr4:76,422,552-76,821,049	llelic Imbalanc	398498	q21.1	0.26850892	34.52380952
chr4:76,821,049-76,977,188	CN Loss	156140	q21.1	0.30613748	10
chr4:76,821,049-77,101,142	LOH	280094	q21.1	0.1706576	6.18556701
chr4:77,031,298-77,101,142	CN Loss	69845	q21.1	0	3.846153846
chr4:77,101,142-77,188,280	llelic Imbalanc	87139	q21.1	13.121715	11.76470588
chr4:77,188,280-77,455,240	CN Loss	266961	q21.1	0.9671861	18.94736842
chr4:77,582,500-77,731,535	CN Loss	149036	q21.1	0	15.38461538
chr4:77,731,535-77,834,775	LOH	103241	q21.1	6.40255715	0
chr4:77,951,421-78,011,984	llelic Imbalanc	60564	q21.1	0	38.88888889
chr4:77,951,421-78,011,984	CN Loss	60564	q21.1	0	38.88888889
chr4:78,193,829-78,583,979	llelic Imbalanc	390151	q21.1	2.51441753	63.38028169
chr4:78,583,979-78,722,498	CN Loss	138520	q21.1	0	9.523809524
chr4:78,583,979-78,722,498	LOH	138520	q21.1	0	9.523809524
chr4:78,805,642-79,025,335	CN Loss	219694	q21.1 - q21.21	0	53.22580645
chr4:78,722,498-80,804,795	llelic Imbalanc	2082298	q21.1 - q21.21	0.17173343	29.40140845
chr4:79,433,150-81,242,404	CN Loss	1809255	q21.21	0.33632646	19.39655172
chr4:80,814,678-81,665,259	LOH	850582	q21.21	1.0990135	4.14507772
chr4:81,665,259-81,831,711	CN Loss	166453	q21.21	10.4438517	20.58823529
chr4:82,135,334-82,266,780	CN Loss	131447	q21.21	0	33.33333333
chr4:82,596,776-82,905,995	CN Loss	309220	q21.22	0	28.8
chr4:81,665,259-83,914,712	llelic Imbalanc	2249454	21.21 - q21.2	1.0402529	30.15625
chr4:83,667,232-83,914,712	CN Loss	247481	q21.22	0	35.55555556
chr4:84,163,106-84,176,912	CN Loss	13807	q21.23	0	85.71428571
chr4:84,163,106-85,386,296	llelic Imbalanc	1223191	q21.23	0.46207049	32.53333333
chr4:84,550,745-85,386,296	CN Loss	835552	q21.23	0.67643986	32.15547703
chr4:85,803,430-86,288,461	LOH	485032	q21.23	0	1.333333333
chr4:86,288,461-86,388,903	llelic Imbalanc	100443	q21.23	0	57.14285714
chr4:86,388,903-86,643,916	LOH	255014	q21.23	0.64388874	4.87804878
chr4:86,509,438-86,643,916	CN Loss	134479	q21.23	1.22101756	7.317073171
chr4:86,823,672-87,157,693	CN Loss	334022	q21.23 - q21.3	11.2451014	15.90909091
chr4:86,643,916-87,435,032	llelic Imbalanc	791117	q21.23 - q21.3	5.76729582	27.57009346
chr4:87,372,837-87,435,032	CN Loss	62196	q21.3	0	18.75
chr4:87,435,032-87,773,032	LOH	338001	q21.3	0	5.172413793
chr4:88,058,145-88,184,438	CN Loss	126294	q22.1	0	24.13793103

chr4:88,184,438-89,091,732	llelic Imbalanc	907295	q22.1	0.48672205	32.29461756
chr4:88,669,197-88,771,029	CN Loss	101833	q22.1	0	23.91304348
chr4:89,010,071-89,091,732	CN Loss	81662	q22.1	0	25.92592593
chr4:89,248,599-89,606,630	LOH	358032	q22.1	0	5.970149254
chr4:89,606,630-89,926,240	CN Loss	319611	q22.1	0	37.14285714
chr4:90,019,399-90,246,992	CN Loss	227594	q22.1	2.81115852	16.86746988
chr4:89,606,630-90,762,629	llelic Imbalanc	1156000	q22.1	1.72482848	32.58426966
chr4:90,426,875-90,762,629	CN Loss	335755	q22.1	4.03301226	29
chr4:90,762,629-91,069,921	LOH	307293	q22.1	0	3.896103896
chr4:90,827,075-91,069,921	CN Loss	242847	q22.1	0	1.851851852
chr4:91,254,159-91,297,396	CN Loss	43238	q22.1	12.3343433	73.91304348
chr4:91,539,379-91,577,418	CN Loss	38040	q22.1	0	50
chr4:91,069,921-92,262,061	llelic Imbalanc	1192141	q22.1	6.18148875	42.06896552
chr4:92,123,166-92,262,061	CN Loss	138896	q22.1	1.44785629	47.36842105
chr4:92,960,229-93,136,775	llelic Imbalanc	176547	q22.1	0	21.875
chr4:92,960,229-93,136,775	CN Loss	176547	q22.1	0	21.875
chr4:93,136,775-93,449,109	LOH	312335	q22.1	0	3.571428571
chr4:93,267,883-93,320,547	CN Loss	52665	q22.1	0	0
chr4:93,360,927-93,449,109	CN Loss	88183	q22.1	0	0
chr4:93,449,109-93,560,286	llelic Imbalanc	111178	q22.1	0	65.71428571
chr4:93,560,286-93,759,015	CN Loss	198730	q22.1 - q22.2	1.44568734	25
chr4:93,759,015-94,218,345	llelic Imbalanc	459331	q22.2	0.59717414	38.29787234
chr4:94,218,345-94,252,615	CN Loss	34271	q22.2	0	16.66666667
chr4:94,362,035-94,433,584	llelic Imbalanc	71550	q22.2	0	33.33333333
chr4:94,362,035-94,433,584	CN Loss	71550	q22.2	0	33.33333333
chr4:94,433,584-94,528,181	LOH	94598	q22.2	93.6023341	5.555555556
chr4:94,528,181-94,920,047	CN Loss	391867	q22.2	100	33.89830508
chr4:94,528,181-95,821,114	llelic Imbalanc	1292934	q22.2 - q22.3	45.5772264	48.03625378
chr4:95,821,114-95,951,749	CN Loss	130636	q22.3	0	29.62962963
chr4:95,951,749-97,102,360	llelic Imbalanc	1150612	q22.3	0	39.12037037
chr4:97,327,134-98,132,446	CN Loss	805313	q22.3	0	46.15384615
chr4:98,132,446-98,818,399	llelic Imbalanc	685954	q22.3 - q23	3.08680041	31.91489362
chr4:98,409,423-98,818,399	CN Loss	408977	q22.3 - q23	0	31.16883117
chr4:99,011,656-99,077,411	CN Loss	65756	q23	0	15.38461538
chr4:99,011,656-99,323,870	llelic Imbalanc	312215	q23	0	34.56790123
chr4:99,283,138-99,323,870	CN Loss	40733	q23	0	70
chr4:99,673,131-99,811,388	CN Loss	138258	q23	1.86464338	11.42857143
chr4:99,673,131-99,811,388	LOH	138258	q23	1.86464338	11.42857143
chr4:99,811,388-99,819,409	CN Gain	8022	q23	58.6834559	0
chr4:99,819,409-99,835,914	CN Loss	16506	q23	0	0
chr4:99,835,914-99,990,281	llelic Imbalanc	154368	q23	0	38.70967742
chr4:99,990,281-100,043,448	CN Loss	53168	q23	0	56.25
chr4:100,172,315-100,251,678	CN Loss	79364	q23	0	50
chr4:100,043,448-100,663,160	llelic Imbalanc	619713	q23	0	40
chr4:100,299,796-100,967,351	CN Loss	667556	q23	0.43996375	15.24390244
chr4:100,663,160-100,967,351	LOH	304192	q23	0.9655118	5.797101449
chr4:101,298,204-102,248,859	CN Loss	950656	q24	0.41339918	33.10810811

chr4:100,967,351-102,692,720	llelic Imbalanc	1725370	q23 - q24	0.89291044	32.2
chr4:102,551,391-102,692,720	CN Loss	141330	q24	0	28.88888889
chr4:102,747,433-103,111,141	llelic Imbalanc	363709	q24	4.21436977	32.29166667
chr4:103,111,141-103,337,509	LOH	226369	q24	0	5.333333333
chr4:104,385,458-104,773,395	llelic Imbalanc	387938	q24	3.1726801	38.83495146
chr4:102,747,433-107,247,290	CN Loss	4499858	q24	4.31064809	28.74306839
chr4:104,773,395-105,418,903	LOH	645509	q24	10.2317864	6.504065041
chr4:106,936,796-107,301,659	LOH	364864	q24	4.52854907	2.5
chr4:107,301,659-107,975,407	CN Loss	673749	q24 - q25	0.74033615	20.77922078
chr4:107,511,823-107,768,812	LOH	256990	q24 - q25	1.94093911	3.448275862
chr4:107,768,812-107,975,407	llelic Imbalanc	206596	q25	0	34.69387755
chr4:108,058,619-108,621,794	CN Loss	563176	q25	4.71238958	40.60606061
chr4:108,621,794-109,248,905	llelic Imbalanc	627112	q25	0	30.92105263
chr4:108,964,771-109,248,905	CN Loss	284135	q25	0	20.73170732
chr4:109,667,597-110,422,963	CN Loss	755367	q25	0.27086207	27.31958763
chr4:110,209,800-110,422,963	LOH	213164	q25	0	9.523809524
chr4:110,422,963-110,582,562	llelic Imbalanc	159600	q25	0	37.93103448
chr4:110,582,562-111,078,389	CN Loss	495828	q25	1.31537815	28.47682119
chr4:110,902,754-111,078,389	llelic Imbalanc	175636	q25	0.54089447	33.87096774
chr4:111,269,626-111,498,386	CN Loss	228761	q25	2.12056304	33.78378378
chr4:111,556,457-111,740,828	LOH	184372	q25	0	2.5
chr4:111,740,828-112,292,808	llelic Imbalanc	551981	q25	1.06815464	32.5
chr4:112,292,808-112,521,966	LOH	229159	q25	5.39540404	0
chr4:111,556,457-114,227,430	CN Loss	2670974	q25 - q26	0.9434764	17.21518987
chr4:112,677,096-113,595,720	LOH	918625	q25	0.52306493	4.219409283
chr4:113,595,720-114,227,430	llelic Imbalanc	631711	q25 - q26	0.28905669	23.61111111
chr4:114,392,528-114,767,055	llelic Imbalanc	374528	q26	0	39.68253968
chr4:114,392,528-114,767,055	CN Loss	374528	q26	0	39.68253968
chr4:114,954,697-115,210,714	llelic Imbalanc	256018	q26	7.23701942	42.42424242
chr4:115,296,794-115,541,593	LOH	244800	q26	2.76308318	1.388888889
chr4:114,954,697-116,865,887	CN Loss	1911191	q26	7.07355103	33.33333333
chr4:115,541,593-116,865,887	llelic Imbalanc	1324295	q26	8.29853492	36.89320388
chr4:116,989,604-118,203,302	llelic Imbalanc	1213699	q26	0.07019868	34.90566038
chr4:116,989,604-118,203,302	CN Loss	1213699	q26	0.07019868	34.90566038
chr4:118,203,302-119,148,039	LOH	944738	q26	0.52480214	7.604562738
chr4:118,375,193-119,257,167	CN Loss	881975	q26	1.82329638	11.49425287
chr4:119,148,039-119,980,443	llelic Imbalanc	832405	q26	7.38055079	42.85714286
chr4:119,980,443-120,086,706	CN Loss	106264	q26	0.10163462	25.45454545
chr4:120,086,706-120,417,089	llelic Imbalanc	330384	q26	0	20.27027027
chr4:120,417,089-120,902,533	LOH	485445	q26 - q27	0.85900742	6.666666667
chr4:120,417,089-121,308,615	CN Loss	891527	q26 - q27	0.65225243	16.66666667
chr4:120,902,533-121,713,771	llelic Imbalanc	811239	q27	1.75349281	26.69322709
chr4:121,713,771-121,761,296	CN Loss	47526	q27	0	40
chr4:121,761,296-122,302,771	llelic Imbalanc	541476	q27	1.5969343	33.54037267
chr4:121,947,696-122,302,771	CN Loss	355076	q27	2.43526016	24.52830189
chr4:122,302,771-122,428,518	LOH	125748	q27	39.3909994	3.703703704
chr4:122,428,518-122,749,987	CN Loss	321470	q27	0	27.88461538

chr4:122,428,518-123,377,806	llelic Imbalanc	949289	q27	0	25.43859649
chr4:123,377,806-123,658,274	CN Loss	280469	q27	0.33978921	26.08695652
chr4:123,876,777-124,949,411	llelic Imbalanc	1072635	q28.1	64.2642318	30.60498221
chr4:123,760,738-125,505,580	CN Loss	1744843	q27 - q28.1	57.0502086	28.47682119
chr4:124,949,411-125,259,845	LOH	310435	q28.1	98.6090441	2.739726027
chr4:125,505,580-125,922,495	llelic Imbalanc	416916	q28.1	0	40.35087719
chr4:125,922,495-126,181,406	CN Loss	258912	q28.1	0	43.47826087
chr4:126,181,406-126,523,549	llelic Imbalanc	342144	q28.1	0	38.61386139
chr4:126,250,908-126,523,549	CN Loss	272642	q28.1	0	35
chr4:126,919,544-127,154,401	CN Loss	234858	q28.1	0	4.285714286
chr4:126,919,544-127,154,401	LOH	234858	q28.1	0	4.285714286
chr4:127,399,093-128,637,248	CN Loss	1238156	q28.1	0	38.81987578
chr4:127,154,401-129,080,997	llelic Imbalanc	1926597	q28.1 - q28.2	0.01609056	35.74561404
chr4:129,080,997-129,297,222	CN Loss	216226	q28.2	1.81061394	25
chr4:129,297,222-130,049,190	llelic Imbalanc	751969	q28.2	0	26.66666667
chr4:130,049,190-130,078,019	CN Loss	28830	q28.2	10.6073745	33.33333333
chr4:130,078,019-130,156,271	llelic Imbalanc	78253	q28.2	0	68.75
chr4:130,156,271-130,300,752	CN Loss	144482	q28.2	1.56075885	43.75
chr4:130,300,752-130,379,101	llelic Imbalanc	78350	q28.2	0	62.06896552
chr4:130,379,101-130,555,550	LOH	176450	q28.2	0	8.064516129
chr4:130,555,550-130,763,440	llelic Imbalanc	207891	q28.2	1.28385204	27.94117647
chr4:130,555,550-130,763,440	CN Loss	207891	q28.2	1.28385204	27.94117647
chr4:130,763,440-130,951,198	LOH	187759	q28.2	0	2.564102564
chr4:130,951,198-132,017,621	llelic Imbalanc	1066424	q28.2 - q28.3	0.05213691	45.13888889
chr4:131,530,288-132,017,621	CN Loss	487334	q28.3	0	38.18181818
chr4:132,409,866-132,690,294	CN Loss	280429	q28.3	48.1503274	18.18181818
chr4:133,099,504-133,470,596	llelic Imbalanc	371093	q28.3	0.50149289	51.38888889
chr4:133,099,504-133,470,596	CN Loss	371093	q28.3	0.50149289	51.38888889
chr4:133,668,736-134,646,431	llelic Imbalanc	977696	q28.3	0.02761597	23.16602317
chr4:133,668,736-134,646,431	CN Loss	977696	q28.3	0.02761597	23.16602317
chr4:134,972,732-136,033,877	llelic Imbalanc	1061146	q28.3	0.30551904	41.33858268
chr4:135,479,820-136,212,036	CN Loss	732217	q28.3	0	23.91304348
chr4:136,033,877-136,527,246	LOH	493370	q28.3	0.05330696	3.149606299
chr4:136,527,246-136,605,603	llelic Imbalanc	78358	q28.3	0	36.84210526
chr4:136,331,932-137,226,322	CN Loss	894391	q28.3	0.02940552	22.85714286
chr4:136,963,134-137,226,322	LOH	263189	q28.3	0	7.272727273
chr4:137,226,322-138,067,738	llelic Imbalanc	841417	q28.3	0.96408911	41.17647059
chr4:137,684,554-138,067,738	CN Loss	383185	q28.3	0.6023216	37.77777778
chr4:138,611,743-138,899,513	CN Loss	287771	q28.3	0	45.23809524
chr4:139,026,725-139,091,963	CN Loss	65239	q28.3	0	57.14285714
chr4:138,899,513-139,909,323	llelic Imbalanc	1009811	q28.3 - q31.1	0.10764401	36.70886076
chr4:139,157,769-139,909,323	CN Loss	751555	q28.3 - q31.1	0	35.91836735
chr4:140,006,021-140,046,840	CN Loss	40820	q31.1	0	0
chr4:139,909,323-140,421,785	LOH	512463	q31.1	1.09744723	4.705882353
chr4:140,078,513-140,606,564	CN Loss	528052	q31.1	1.78808486	19.13043478
chr4:140,421,785-140,878,565	llelic Imbalanc	456781	q31.1	0.83585096	30.51948052
chr4:140,878,565-141,021,991	CN Loss	143427	q31.1	0	58.82352941

chr4:141,021,991-141,298,615	llelic Imbalanc	276625	q31.1	0	25.3968254
chr4:141,298,615-141,506,384	CN Loss	207770	q31.1 - q31.21	0	12.28070175
chr4:141,327,585-141,506,384	LOH	178800	q31.1 - q31.21	0	6.52173913
chr4:141,506,384-141,903,213	llelic Imbalanc	396830	q31.21	0	26.72413793
chr4:141,982,796-142,117,537	llelic Imbalanc	134742	q31.21	0	41.17647059
chr4:141,903,213-142,255,857	CN Loss	352645	q31.21	0.92472862	32.32323232
chr4:142,117,537-142,448,731	LOH	331195	q31.21	0.98461929	6.25
chr4:142,448,731-142,570,379	CN Loss	121649	q31.21	0	56.52173913
chr4:142,448,731-143,053,760	llelic Imbalanc	605030	q31.21	0	43.19526627
chr4:142,986,877-143,530,248	CN Loss	543372	q31.21	0	34.9112426
chr4:143,585,305-143,746,543	CN Loss	161239	q31.21	0	2.702702703
chr4:143,530,248-143,929,482	LOH	399235	q31.21	1.89738349	3.409090909
chr4:143,929,482-144,039,442	llelic Imbalanc	109961	q31.21	0	77.27272727
chr4:144,039,442-144,284,908	CN Loss	245467	q31.21	0.10592098	1.960784314
chr4:144,039,442-144,527,297	LOH	487856	q31.21	0.05329452	2.97029703
chr4:144,527,297-144,589,777	CN Loss	62481	q31.21	0	38.46153846
chr4:144,589,777-145,339,401	llelic Imbalanc	749625	q31.21	50.0482909	49.68553459
chr4:145,339,401-145,577,503	CN Loss	238103	q31.21	0	24.44444444
chr4:145,577,503-145,660,312	LOH	82810	q31.21	0	0
chr4:145,660,312-146,630,062	llelic Imbalanc	969751	q31.21	0.23892756	45.25139665
chr4:145,660,312-146,630,062	CN Loss	969751	q31.21	0.23892756	45.25139665
chr4:146,784,148-147,964,898	llelic Imbalanc	1180751	31.21 - q31.2	1.11835698	28.95622896
chr4:146,784,148-148,496,395	CN Loss	1712248	31.21 - q31.2	0.77290251	24.07002188
chr4:147,964,898-148,321,100	LOH	356203	q31.22	0.00814145	4.210526316
chr4:148,845,684-149,023,709	LOH	178026	q31.23	0	4.761904762
chr4:148,845,684-151,285,450	CN Loss	2439767	q31.23 - q31.3	0.60641061	33.4375
chr4:149,377,041-151,316,476	llelic Imbalanc	1939436	q31.23 - q31.3	0.6720514	35.0877193
chr4:151,316,476-151,951,119	CN Loss	634644	q31.3	1.47941441	50
chr4:151,951,119-152,067,118	llelic Imbalanc	116000	q31.3	0	37.5
chr4:152,067,118-152,176,795	CN Loss	109678	q31.3	43.4065483	50
chr4:152,351,437-154,331,334	CN Loss	1979898	q31.3	5.18203725	42.35976789
chr4:152,819,894-154,496,452	llelic Imbalanc	1676559	q31.3	3.27725018	38.74709977
chr4:154,496,452-154,623,015	CN Loss	126564	q31.3	0	24.32432432
chr4:154,623,015-154,745,297	llelic Imbalanc	122283	q31.3	0	48.27586207
chr4:154,745,297-155,524,041	CN Loss	778745	q31.3	1.28565999	36.65338645
chr4:155,969,670-156,191,460	CN Loss	221791	q32.1	0	54.54545455
chr4:155,895,071-156,856,615	llelic Imbalanc	961545	q32.1	0.98861831	33.81294964
chr4:156,344,869-156,856,615	CN Loss	511747	q32.1	1.77939837	21.76870748
chr4:156,856,615-156,949,680	LOH	93066	q32.1	2.36393918	3.448275862
chr4:156,949,680-157,241,226	CN Loss	291547	q32.1	8.58080715	36.17021277
chr4:157,241,226-157,390,844	llelic Imbalanc	149619	q32.1	1.11818097	51.11111111
chr4:157,638,098-158,206,748	CN Loss	568651	q32.1	0.16706234	7.407407407
chr4:157,638,098-158,206,748	LOH	568651	q32.1	0.16706234	7.407407407
chr4:158,206,748-158,391,545	llelic Imbalanc	184798	q32.1	0	55
chr4:158,430,475-158,701,433	LOH	270959	q32.1	0	1.063829787
chr4:158,391,545-158,872,502	CN Loss	480958	q32.1	0.75266604	18.66666667
chr4:158,872,502-158,941,632	llelic Imbalanc	69131	q32.1	0	65.2173913

chr4:158,941,632-159,324,705	CN Loss	383074	q32.1	0	26.82926829
chr4:159,324,705-160,642,586	llelic Imbalanc	1317882	q32.1	0.05440552	43.93939394
chr4:159,442,774-162,193,441	CN Loss	2750668	q32.1 - q32.2	12.973108	30.11889036
chr4:160,642,586-161,231,572	LOH	588987	q32.1	9.22687466	5.405405405
chr4:161,231,572-161,763,237	llelic Imbalanc	531666	q32.1	0	19.16167665
chr4:162,305,919-162,513,063	CN Loss	207145	q32.2	1.15040745	27.77777778
chr4:162,305,919-162,718,387	llelic Imbalanc	412469	q32.2	0.57774179	28.57142857
chr4:162,718,387-163,970,842	CN Loss	1252456	q32.2	1.16626945	37.64367816
chr4:164,262,619-165,628,828	CN Loss	1366210	q32.2 - q32.3	0.58190218	31.5270936
chr4:166,163,646-166,304,172	CN Loss	140527	q32.3	0	35.29411765
chr4:165,628,828-167,129,049	llelic Imbalanc	1500222	q32.3	2.18894416	32.73137698
chr4:166,384,611-166,428,758	CN Loss	44148	q32.3	0	16.66666667
chr4:167,129,049-167,488,464	LOH	359416	q32.3	0.17723245	3.448275862
chr4:167,129,049-168,105,966	CN Loss	976918	q32.3	0.13778038	19.14893617
chr4:167,488,464-168,574,933	llelic Imbalanc	1086470	q32.3	0.83914037	31.43939394
chr4:168,574,933-169,168,789	CN Loss	593857	q32.3	51.2238657	43.15068493
chr4:168,995,618-169,168,789	llelic Imbalanc	173172	q32.3	59.9170762	48.48484848
chr4:169,326,359-169,564,869	llelic Imbalanc	238511	q32.3	0	18.18181818
chr4:169,326,359-169,564,869	CN Loss	238511	q32.3	0	18.18181818
chr4:169,610,170-169,679,327	llelic Imbalanc	69158	q32.3	0	23.80952381
chr4:169,610,170-169,679,327	CN Loss	69158	q32.3	0	23.80952381
chr4:169,679,327-169,969,058	LOH	289732	q32.3	34.0467537	6.25
chr4:169,969,058-170,157,854	llelic Imbalanc	188797	q32.3 - q33	0	25.49019608
chr4:169,768,481-170,984,555	CN Loss	1216075	q32.3 - q33	8.21052008	18.81533101
chr4:170,157,854-170,780,414	LOH	622561	q33	0.19307376	6.976744186
chr4:170,984,555-171,298,206	llelic Imbalanc	313652	q33	3.08655161	33.33333333
chr4:171,298,206-171,578,829	LOH	280624	q33	0	2.941176471
chr4:171,578,829-172,068,794	llelic Imbalanc	489966	q33 - q34.1	0	23.02158273
chr4:171,298,206-173,423,734	CN Loss	2125529	q33 - q34.1	1.56347035	20.07434944
chr4:172,068,794-172,709,798	LOH	641005	q34.1	1.80872506	7.189542484
chr4:172,709,798-173,423,734	llelic Imbalanc	713937	q34.1	3.03080388	35.39325843
chr4:173,596,998-173,842,462	CN Loss	245465	q34.1	0	53.03030303
chr4:173,596,998-174,425,690	llelic Imbalanc	828693	q34.1	0	31.01604278
chr4:173,876,669-174,425,690	CN Loss	549022	q34.1	0	18.75
chr4:174,572,951-175,274,119	CN Loss	701169	q34.1	0	26.3681592
chr4:175,598,265-175,846,268	CN Loss	248004	q34.1	0.78587759	43.82022472
chr4:175,274,119-176,257,583	llelic Imbalanc	983465	q34.1	0.19817706	36.78571429
chr4:176,257,583-176,475,752	LOH	218170	q34.1 - q34.2	2.5837768	2.272727273
chr4:176,257,583-176,825,261	CN Loss	567679	q34.1 - q34.2	10.3361413	29.23076923
chr4:177,009,468-177,108,204	llelic Imbalanc	98737	q34.2	100	58.33333333
chr4:177,009,468-177,197,579	CN Loss	188112	q34.2	100	22.05882353
chr4:177,108,204-177,382,828	LOH	274625	q34.2	56.1513196	3.191489362
chr4:177,382,828-177,499,642	llelic Imbalanc	116815	q34.2	0	58.73015873
chr4:177,499,642-177,896,143	CN Loss	396502	q34.2 - q34.3	0.31349227	6.086956522
chr4:177,499,642-177,896,143	LOH	396502	q34.2 - q34.3	0.31349227	6.086956522
chr4:177,978,862-178,152,942	LOH	174081	q34.3	3.23701746	0
chr4:177,978,862-178,269,367	CN Loss	290506	q34.3	4.87495912	23.80952381

chr4:178,269,367-178,494,166	llelic Imbalanc	224800	q34.3	5.02715759	49.18032787
chr4:178,494,166-178,924,593	CN Loss	430428	q34.3	5.98823959	15.17857143
chr4:178,698,464-178,924,593	LOH	226130	q34.3	4.04194066	9.375
chr4:178,924,593-179,213,415	llelic Imbalanc	288823	q34.3	0.15580531	41.97530864
chr4:179,213,415-179,275,334	CN Loss	61920	q34.3	11.2372616	20
chr4:179,377,371-179,692,342	CN Loss	314972	q34.3	0.64291633	29.05982906
chr4:179,692,342-179,935,593	llelic Imbalanc	243252	q34.3	0	31.14754098
chr4:180,017,001-180,133,971	LOH	116971	q34.3	0	1.724137931
chr4:179,935,593-180,220,175	CN Loss	284583	q34.3	0	31.09243697
chr4:180,301,427-181,126,519	llelic Imbalanc	825093	q34.3	0	37.29508197
chr4:180,416,368-181,126,519	CN Loss	710152	q34.3	0	33.49514563
chr4:181,126,519-181,175,075	LOH	48557	q34.3	8.53859461	12.5
chr4:181,175,075-181,448,283	CN Loss	273209	q34.3	1.42492167	35.77981651
chr4:181,529,150-183,059,560	CN Loss	1530411	q34.3	1.02083755	37.69363167
chr4:181,448,283-183,250,176	llelic Imbalanc	1801894	q34.3 - q35.1	0.97852647	40
chr4:183,250,176-183,301,830	CN Loss	51655	q35.1	0	6.666666667
chr4:183,250,176-183,301,830	LOH	51655	q35.1	0	6.666666667
chr4:183,301,830-183,632,526	llelic Imbalanc	330697	q35.1	0.58754869	30.95238095
chr4:183,361,350-183,578,448	CN Loss	217099	q35.1	0.89498752	31.46067416
chr4:183,632,526-184,290,650	CN Loss	658125	q35.1	0	9.770114943
chr4:183,632,526-184,290,650	LOH	658125	q35.1	0	9.770114943
chr4:184,449,597-185,860,000	llelic Imbalanc	1410404	q35.1	1.22929404	43.40044743
chr4:184,449,597-186,027,681	CN Loss	1578085	q35.1	1.28149072	42.61363636
chr4:186,027,681-187,115,736	llelic Imbalanc	1088056	q35.1 - q35.2	3.11574323	28.0653951
chr4:186,173,709-187,115,736	CN Loss	942028	q35.1 - q35.2	3.59872912	24.32432432
chr4:187,137,157-187,194,339	CN Loss	57183	q35.2	0	5.263157895
chr4:187,115,736-187,232,970	LOH	117235	q35.2	0	2.127659574
chr4:187,232,970-188,250,154	CN Loss	1017185	q35.2	7.17362837	24.6835443
chr4:187,232,970-189,397,229	llelic Imbalanc	2164260	q35.2	6.20706671	27.16049383
chr4:188,459,555-188,814,236	CN Loss	354682	q35.2	0	37.11340206
chr4:189,150,386-189,397,229	CN Loss	246844	q35.2	5.15833951	16.48351648
chr4:189,493,726-189,567,921	llelic Imbalanc	74196	q35.2	0	28
chr4:189,567,921-189,783,144	LOH	215224	q35.2	1.0454273	1.351351351
chr4:189,493,726-190,249,909	CN Loss	756184	q35.2	7.73899969	28.90855457
chr4:190,249,909-191,154,276	llelic Imbalanc	904368	q35.2	52.9454303	40.88050314
chr5:0-171,266	ozygous Copy	171267	p15.33	35.7893569	70
chr5:171,266-187,249	llelic Imbalanc	15984	p15.33	0	33.33333333
chr5:171,266-187,249	CN Loss	15984	p15.33	0	33.33333333
chr5:187,249-679,100	LOH	491852	p15.33	26.186589	5.042016807
chr5:679,100-842,061	ozygous Copy	162962	p15.33	100	16.66666667
chr5:842,061-1,268,764	LOH	426704	p15.33	57.0045207	1.333333333
chr5:1,268,764-1,274,450	CN Loss	5687	p15.33	0	50
chr5:1,274,450-1,590,468	LOH	316019	p15.33	11.917992	3.296703297
chr5:1,590,468-9,061,666	llelic Imbalanc	7471199	15.33 - p15.3	4.65543812	32.15619694
chr5:9,061,666-9,401,380	LOH	339715	p15.31	0	10.96774194
chr5:1,590,468-17,624,308	CN Loss	16033841	p15.33 - p15.1	6.04339946	24.29419169
chr5:9,401,380-11,588,391	llelic Imbalanc	2187012	p15.31 - p15.2	2.2361113	19.7320341

chr5:11,588,391-17,624,308	LOH	6035918	p15.2 - p15.1	9.48104489	13.7195122
chr5:21,693,683-22,139,823	LOH	446141	p14.3	21.7386919	0.980392157
chr5:23,629,083-23,961,489	LOH	332407	p14.2	0	4.597701149
chr5:24,594,590-25,386,250	LOH	791661	p14.2 - p14.1	0.95103959	4.678362573
chr5:27,289,506-27,707,732	LOH	418227	p14.1	0.72998809	2.298850575
chr5:29,155,679-29,349,820	LOH	194142	p13.3	0	1.538461538
chr5:32,635,753-32,855,142	LOH	219390	p13.3	0	1.282051282
chr5:33,610,551-33,893,249	LOH	282699	p13.3 - p13.2	0	5.769230769
chr5:35,345,190-35,539,689	LOH	194500	p13.2	0	1.666666667
chr5:36,034,269-36,437,983	LOH	403715	p13.2	0	0.806451613
chr5:36,857,320-37,567,455	LOH	710136	p13.2	0.30529406	2.631578947
chr5:39,194,314-39,432,113	LOH	237800	p13.1	3.11271284	0
chr5:40,086,270-40,280,351	LOH	194082	p13.1	0	0
chr5:43,611,131-44,414,288	LOH	803158	p12	0	4.419889503
chr5:44,626,545-44,982,657	LOH	356113	p12	0	1.369863014
chr5:45,213,365-46,383,335	LOH	1169971	p12 - p11	16.2994778	5.681818182
chr5:49,519,999-49,869,289	LOH	349291	q11.1	13.2978327	4
chr5:51,134,835-51,703,846	LOH	569012	q11.2	0.79436074	1.639344262
chr5:52,390,556-53,190,951	LOH	800396	q11.2	2.28949456	2.183406114
chr5:54,059,050-54,233,094	LOH	174045	q11.2	0	0
chr5:56,065,790-56,346,980	LOH	281191	q11.2	0	0
chr5:56,953,995-57,441,565	LOH	487571	q11.2	2.13015567	1.183431953
chr5:58,448,250-58,482,231	CN Gain	33982	q11.2	0	66.66666667
chr5:61,541,065-61,966,053	LOH	424989	q12.1	4.14270521	2.298850575
chr5:63,974,850-64,240,189	LOH	265340	q12.3	0	0
chr5:66,032,738-66,223,931	LOH	191194	q12.3	17.2945662	2.469135802
chr5:66,542,489-67,021,859	LOH	479371	q12.3 - q13.1	0.04610218	5.714285714
chr5:67,819,927-68,035,094	LOH	215168	q13.1	0	1.449275362
chr5:70,306,646-70,975,556	LOH	668911	q13.2	53.1288215	0
chr5:71,850,985-73,104,047	LOH	1253063	q13.2	0.39551116	2.608695652
chr5:74,540,504-75,224,301	LOH	683798	q13.3	0	3.797468354
chr5:79,894,019-80,299,218	LOH	405200	q14.1	8.90624113	3.296703297
chr5:87,101,256-87,851,585	LOH	750330	q14.3	0.55655586	4.109589041
chr5:90,379,900-91,129,810	LOH	749911	q14.3	1.65859903	1.630434783
chr5:96,885,012-97,255,606	LOH	370595	q15	29.6059839	0
chr5:100,594,279-101,119,869	LOH	525591	q21.1	0	1.242236025
chr5:102,282,427-105,018,857	LOH	2736431	q21.1 - q21.3	6.21886911	2.698650675
chr5:107,710,591-107,949,972	LOH	239382	q21.3	0	1.449275362
chr5:108,636,753-109,214,525	LOH	577773	q21.3	1.61309305	0.632911392
chr5:109,351,647-109,610,136	LOH	258490	q21.3 - q22.1	10.8716425	0
chr5:109,636,457-110,052,412	LOH	415956	q22.1	6.06195382	3.488372093
chr5:110,236,986-110,966,211	LOH	729226	q22.1	0.70129247	6.481481481
chr5:111,159,257-111,515,833	LOH	356577	q22.1 - q22.2	0	1.020408163
chr5:113,197,115-113,396,331	LOH	199217	q22.3	3.84959039	1.333333333
chr5:115,352,258-115,718,756	LOH	366499	q23.1	100	2.919708029
chr5:117,955,285-118,404,207	LOH	448923	q23.1	0	0.819672131
chr5:119,394,798-119,591,119	LOH	196322	q23.1	0	0

chr5:119,906,394-120,207,420	LOH	301027	q23.1	0.38534877	2.739726027
chr5:121,463,294-121,501,017	CN Gain	37724	q23.2	0	0
chr5:121,463,294-121,501,017	LOH	37724	q23.2	0	0
chr5:122,569,749-122,653,949	CN Gain	84201	q23.2	0.695962	31.03448276
chr5:126,701,718-126,964,119	LOH	262402	q23.2	0	0
chr5:127,237,460-127,622,815	LOH	385356	q23.2 - q23.3	1.52897977	0
chr5:128,861,968-129,447,523	LOH	585556	q23.3	0.18358651	3.731343284
chr5:135,841,626-136,263,448	LOH	421823	q31.1 - q31.2	2.6914196	0
chr5:139,945,489-140,565,056	LOH	619568	q31.3	7.91407548	0
chr5:142,184,170-142,499,716	LOH	315547	q31.3	0.58058096	0.943396226
chr5:142,617,723-142,969,050	LOH	351328	q31.3	0	1.25
chr5:143,484,790-143,861,791	LOH	377002	q31.3	0	1.904761905
chr5:147,392,313-147,867,644	LOH	475332	q32	0.47651005	0.571428571
chr5:148,989,384-149,239,019	LOH	249636	q32	0.60848839	1.449275362
chr5:150,573,287-150,887,874	LOH	314588	q33.1	2.04998935	0
chr5:153,093,457-153,666,372	LOH	572916	q33.2	0	3.144654088
chr5:159,326,487-159,694,018	LOH	367532	q33.3	0.35724878	1.351351351
chr5:160,353,716-160,541,680	llelic Imbalanc	187965	q34	0	41.79104478
chr5:160,353,716-160,541,680	CN Gain	187965	q34	0	41.79104478
chr5:160,698,779-160,909,825	LOH	211047	q34	18.6461719	0
chr5:164,333,084-164,584,228	LOH	251145	q34	0	1.098901099
chr5:165,308,506-165,652,336	LOH	343831	q34	0	1.204819277
chr5:166,451,018-166,789,764	LOH	338747	q34	0	2.43902439
chr5:167,905,143-168,207,901	LOH	302759	q34	9.59578277	0
chr5:170,276,997-170,831,214	LOH	554218	q35.1	0	3.921568627
chr5:171,296,159-171,554,503	LOH	258345	q35.1	0	4.494382022
chr5:174,097,360-174,255,229	LOH	157870	q35.2	0.88047685	0
chr5:175,329,079-175,985,030	LOH	655952	q35.2	63.809187	1.25
chr5:180,374,710-180,442,420	CN Loss	67711	q35.3	100	100
chr6:1,017,454-1,248,498	LOH	231045	p25.3	0.15581448	2.4
chr6:5,304,880-5,446,315	LOH	141436	p25.1	0	0
chr6:7,976,533-8,391,904	LOH	415372	p24.3	2.08054968	2.994011976
chr6:10,362,636-10,724,540	LOH	361905	p24.3 - p24.2	16.0868628	4
chr6:13,109,565-13,261,508	LOH	151944	p24.1	0.72987897	0
chr6:17,259,752-18,160,757	LOH	901006	p22.3	1.67823708	3.225806452
chr6:20,617,982-20,748,264	CN Gain	130283	p22.3	0	37.5
chr6:21,945,107-22,116,115	LOH	171009	p22.3	2.17007391	2.272727273
chr6:26,948,184-28,448,000	LOH	1499817	p22.2 - p22.1	2.77220672	2.312138728
chr6:29,837,115-29,840,184	CN Gain	3070	p22.1	100	25
chr6:29,840,184-29,936,129	CN Loss	95946	p22.1	100	20
chr6:44,637,299-45,487,429	LOH	850131	p21.1	0	0.995024876
chr6:45,560,227-45,933,311	LOH	373085	p21.1	0.18494495	4.72972973
chr6:47,450,905-48,006,914	LOH	556010	p12.3	0.75448419	3.389830508
chr6:48,369,810-48,858,044	LOH	488235	p12.3	0	1.796407186
chr6:50,610,532-50,961,478	LOH	350947	p12.3	0	2.5
chr6:51,541,665-51,712,802	LOH	171138	p12.3	1.86458802	2.564102564
chr6:53,380,204-53,573,343	LOH	193140	p12.1	0	4.444444444

chr6:55,193,873-55,318,564	LOH	124692	p12.1	0	2.941176471
chr6:64,599,571-65,484,984	LOH	885414	q12	2.6884629	2.702702703
chr6:65,740,336-66,472,768	LOH	732433	q12	2.52282806	3.056768559
chr6:69,523,366-69,676,748	LOH	153383	q12	0	0
chr6:70,110,914-70,367,483	LOH	256570	q13	6.25445787	2.272727273
chr6:71,083,548-71,352,370	LOH	268823	q13	0.32437821	1.639344262
chr6:72,073,888-72,420,692	LOH	346805	q13	4.97024256	2.380952381
chr6:73,215,539-73,475,721	LOH	260183	q13	0.52732318	1.428571429
chr6:74,433,836-74,765,110	LOH	331275	q13	6.11729263	3.47826087
chr6:75,016,336-75,802,325	LOH	785990	q13	12.9449649	5.747126437
chr6:77,438,359-77,458,077	ozygous Copy	19719	q14.1	100	100
chr6:78,386,251-78,731,837	LOH	345587	q14.1	0	1.111111111
chr6:78,948,857-79,198,367	LOH	249511	q14.1	58.1740211	2.666666667
chr6:82,581,703-82,778,487	LOH	196785	q14.1	0	0
chr6:84,740,929-84,977,179	LOH	236251	q14.2 - q14.3	0	2.816901408
chr6:85,832,693-86,050,843	LOH	218151	q14.3	0	0
chr6:88,027,120-88,475,055	LOH	447936	q15	0.13171554	1.612903226
chr6:88,621,938-88,877,437	LOH	255500	q15	8.23173476	1.075268817
chr6:91,370,751-91,609,191	LOH	238441	q15	0	2.912621359
chr6:92,798,872-93,486,581	LOH	687710	q15 - q16.1	1.85252774	1.047120419
chr6:94,273,908-94,490,379	LOH	216472	q16.1	0	1.515151515
chr6:95,417,605-95,891,715	LOH	474111	q16.1	0	2.173913043
chr6:96,491,585-97,095,009	LOH	603425	q16.1	0	3.723404255
chr6:97,344,494-97,539,350	LOH	194857	q16.1	2.66555816	1.639344262
chr6:99,059,045-99,667,106	LOH	608062	q16.1 - q16.2	1.68371265	0.483091787
chr6:100,886,406-101,970,041	LOH	1083636	q16.3	3.86024815	4.776119403
chr6:101,968,092-101,970,041	CN Loss	1950	q16.3	0	0
chr6:102,002,092-102,666,336	LOH	664245	q16.3	0.8322243	5.882352941
chr6:102,737,910-103,143,727	LOH	405818	q16.3	0.21832501	2.105263158
chr6:103,895,802-104,232,763	LOH	336962	q16.3	1.61413339	3.496503497
chr6:105,811,525-106,145,074	LOH	333550	q21	0	4.87804878
chr6:108,026,258-108,291,325	LOH	265068	q21	5.02929448	3.092783505
chr6:109,650,151-110,065,635	LOH	415485	q21	0	1.694915254
chr6:110,358,186-110,714,968	LOH	356783	q21	6.72455449	3.191489362
chr6:113,280,281-113,668,714	LOH	388434	q21	0	0.751879699
chr6:115,313,742-115,986,171	LOH	672430	q22.1	5.57560724	2.684563758
chr6:118,522,252-118,704,050	LOH	181799	q22.31	0	1.470588235
chr6:119,878,645-120,474,705	LOH	596061	q22.31	0	2.162162162
chr6:122,807,960-122,911,752	CN Gain	103793	q22.31	0	52.63157895
chr6:128,365,083-129,092,345	LOH	727263	q22.33	0.77427392	2.884615385
chr6:140,128,805-141,283,627	LOH	1154823	q24.1	0.29485063	2.429149798
chr6:142,332,278-143,219,737	LOH	887460	q24.1 - q24.2	0.18524799	5.263157895
chr6:144,486,442-145,003,942	LOH	517501	q24.2	0	1.775147929
chr6:145,315,629-145,855,597	LOH	539969	q24.2 - q24.3	0.21779068	3.553299492
chr6:149,071,987-149,101,224	CN Gain	29238	q25.1	0	23.07692308
chr6:151,963,731-152,199,090	LOH	235360	q25.1	0	2.702702703
chr6:153,254,061-153,282,855	CN Gain	28795	q25.2	0	40

chr6:153,700,358-153,929,474	LOH	229117	q25.2	0	1.351351351
chr6:154,350,515-154,601,416	LOH	250902	q25.2	0	3
chr6:158,236,637-158,832,838	LOH	596202	q25.3	0	1.123595506
chr6:161,397,625-161,458,279	CN Gain	60655	q26	0	37.5
chr6:162,869,342-163,346,293	LOH	476952	q26	36.6461125	0.540540541
chr6:165,887,857-166,060,323	LOH	172467	q27	0	3.448275862
chr6:166,060,323-166,104,643	CN Gain	44321	q27	11.6719314	52.63157895
chr6:170,756,538-171,115,067	LOH	358530	q27	42.7644626	1.694915254
chr7:3,624,514-4,239,713	LOH	615200	p22.2	47.3074566	3.149606299
chr7:16,115,803-16,316,483	LOH	200681	p21.2	1.26818816	2.898550725
chr7:19,024,048-19,138,365	LOH	114318	p21.1	0	2.816901408
chr7:24,109,160-24,194,264	LOH	85105	p15.3	0	0
chr7:24,534,847-24,859,981	LOH	325135	p15.3	0	0
chr7:30,239,194-30,536,525	LOH	297332	p14.3	0	4.081632653
chr7:33,553,046-33,852,062	LOH	299017	p14.3	0	4
chr7:34,133,219-34,183,469	CN Gain	50251	p14.3	0	58.62068966
chr7:34,696,330-34,944,476	LOH	248147	p14.3	10.2786263	0
chr7:38,744,621-38,984,340	LOH	239720	p14.1	0	2.150537634
chr7:39,787,795-40,699,188	LOH	911394	p14.1	4.54524009	2.912621359
chr7:41,072,332-41,335,710	LOH	263379	p14.1	0	1.149425287
chr7:42,257,207-42,940,417	LOH	683211	p14.1	0	4.132231405
chr7:43,564,172-44,098,773	LOH	534602	p13	22.3282411	4
chr7:47,029,506-47,194,475	LOH	164970	p12.3	1.86398657	1.149425287
chr7:49,285,646-49,519,551	LOH	233906	p12.2	0	2.247191011
chr7:50,669,600-50,899,947	LOH	230348	p12.1	0	2.18579235
chr7:53,491,618-53,785,092	LOH	293475	p12.1	33.6084287	1.785714286
chr7:64,393,226-64,965,449	LOH	572224	q11.21	69.0337508	2.222222222
chr7:68,232,490-68,432,374	LOH	199885	q11.22	0	0
chr7:70,738,618-71,039,506	LOH	300889	q11.22	0	1
chr7:74,396,456-74,457,222	CN Gain	60767	q11.23	100	
chr7:78,048,615-78,176,420	LOH	127806	q21.11	0	5.084745763
chr7:79,343,368-79,888,665	LOH	545298	q21.11	2.47956618	5.084745763
chr7:80,157,962-80,334,027	LOH	176066	q21.11	0	0
chr7:80,334,027-80,735,376	llelic Imbalanc	401350	q21.11	0.0896975	20.16129032
chr7:80,735,376-81,401,399	LOH	666024	q21.11	0	4.6875
chr7:81,401,399-81,711,311	llelic Imbalanc	309913	q21.11	0.3836573	42.1686747
chr7:81,711,311-81,827,199	LOH	115889	q21.11	0	0
chr7:81,827,199-82,978,345	llelic Imbalanc	1151147	q21.11	0.58020442	31.89873418
chr7:82,978,345-83,174,023	LOH	195679	q21.11	1.06143767	0
chr7:83,174,023-84,296,029	llelic Imbalanc	1122007	q21.11	0	23.76237624
chr7:84,296,029-84,545,308	LOH	249280	q21.11	0	2.857142857
chr7:84,545,308-85,031,266	llelic Imbalanc	485959	q21.11	0.8772363	42.63565891
chr7:85,031,266-85,336,758	LOH	305493	q21.11	0	0
chr7:85,336,758-85,609,732	llelic Imbalanc	272975	q21.11	10.7519397	40.47619048
chr7:85,609,732-86,171,210	LOH	561479	q21.11	0.42423746	2
chr7:86,171,210-86,474,584	llelic Imbalanc	303375	21.11 - q21.1	3.58303612	36.53846154
chr7:86,474,584-87,138,588	LOH	664005	q21.12	0	4.444444444

chr7:87,138,588-87,967,706	llelic Imbalanc	829119	q21.12	1.45588445	24.50331126
chr7:87,967,706-88,928,460	LOH	960755	21.12 - q21.1	0.16622361	3.963414634
chr7:80,334,027-97,390,607	CN Gain	17056581	q21.11 - q21.3	2.55263951	22.02268431
chr7:88,928,460-92,043,046	llelic Imbalanc	3114587	q21.13 - q21.2	10.590011	32.09200438
chr7:92,043,046-92,646,299	LOH	603254	q21.2	0	5.555555556
chr7:92,646,299-94,676,388	llelic Imbalanc	2030090	q21.2 - q21.3	0.75789781	24.65331279
chr7:94,676,388-94,908,761	LOH	232374	q21.3	0	2.702702703
chr7:94,908,761-97,182,451	llelic Imbalanc	2273691	q21.3	0.4695451	27.83505155
chr7:97,182,451-97,390,607	LOH	208157	q21.3	4.15938046	3.571428571
chr7:97,390,607-97,403,357	CN Loss	12751	q21.3	97.8666667	
chr7:97,403,357-98,013,109	CN Gain	609753	q21.3 - q22.1	0.97219853	31.42857143
chr7:97,403,357-98,058,319	llelic Imbalanc	654963	q21.3 - q22.1	0.90509068	31.19266055
chr7:98,013,109-98,058,319	High Copy Gai	45211	q22.1	0	25
chr7:98,058,319-98,235,184	CN Gain	176866	q22.1	1.4926639	13.04347826
chr7:98,058,319-98,243,678	LOH	185360	q22.1	1.42426319	12
chr7:98,235,184-98,243,678	High Copy Gai	8495	q22.1	0	0
chr7:98,243,678-98,311,403	llelic Imbalanc	67726	q22.1	0	22.22222222
chr7:98,243,678-100,299,687	CN Gain	2056010	q22.1	7.53878023	9.090909091
chr7:98,311,403-100,995,736	LOH	2684334	q22.1	14.0936315	8.179419525
chr7:100,299,687-100,370,983	High Copy Gai	71297	q22.1	27.2371522	0
chr7:100,370,983-100,995,736	CN Gain	624754	q22.1	32.6373783	13.04347826
chr7:100,995,736-101,022,455	llelic Imbalanc	26720	q22.1	100	50
chr7:100,995,736-101,022,455	High Copy Gai	26720	q22.1	100	50
chr7:101,022,455-102,474,825	CN Gain	1452371	q22.1	28.6913803	11.2244898
chr7:101,022,455-103,070,708	LOH	2048254	q22.1	21.6919492	9.785932722
chr7:102,474,825-102,556,293	High Copy Gai	81469	q22.1	0	8
chr7:102,556,293-102,919,741	CN Gain	363449	q22.1	7.5942088	9.090909091
chr7:102,919,741-103,115,528	High Copy Gai	195788	q22.1	0	18.51851852
chr7:103,070,708-103,115,528	llelic Imbalanc	44821	q22.1	0	57.14285714
chr7:103,115,528-103,137,661	CN Gain	22134	q22.1	0	8.333333333
chr7:103,115,528-103,137,661	LOH	22134	q22.1	0	8.333333333
chr7:103,137,661-103,205,630	llelic Imbalanc	67970	q22.1	0	18.60465116
chr7:103,137,661-103,205,630	High Copy Gai	67970	q22.1	0	18.60465116
chr7:103,205,630-103,233,575	CN Gain	27946	q22.1	0	0
chr7:103,205,630-103,233,575	LOH	27946	q22.1	0	0
chr7:103,233,575-103,237,452	llelic Imbalanc	3878	q22.1	0	33.33333333
chr7:103,233,575-103,237,452	High Copy Gai	3878	q22.1	0	33.33333333
chr7:103,237,452-103,367,051	CN Gain	129600	q22.1	0	6.779661017
chr7:103,237,452-103,488,908	LOH	251457	q22.1	0	8.527131783
chr7:103,367,051-103,450,476	High Copy Gai	83426	q22.1	0	10.90909091
chr7:103,450,476-103,488,908	CN Gain	38433	q22.1	0	6.666666667
chr7:103,488,908-103,501,187	llelic Imbalanc	12280	q22.1	0	26.92307692
chr7:103,488,908-103,501,187	High Copy Gai	12280	q22.1	0	26.92307692
chr7:103,501,187-103,526,426	CN Gain	25240	q22.1	0	4.545454545
chr7:103,501,187-103,526,426	LOH	25240	q22.1	0	4.545454545
chr7:103,526,426-103,619,512	llelic Imbalanc	93087	q22.1	0	21.73913043
chr7:103,526,426-103,619,512	High Copy Gai	93087	q22.1	0	21.73913043

chr7:103,619,512-103,882,162	LOH	262651	q22.1 - q22.2	0	7.766990291
chr7:103,882,162-104,288,333	llelic Imbalanc	406172	q22.2	0	27.77777778
chr7:104,288,333-104,411,040	LOH	122708	q22.2	0	1.5625
chr7:103,619,512-106,750,321	CN Gain	3130810	q22.1 - q22.3	0.58499257	23.64156339
chr7:104,411,040-106,750,321	llelic Imbalanc	2339282	q22.2 - q22.3	0.78293288	26.92307692
chr7:106,750,321-107,463,111	LOH	712791	q22.3 - q31.1	0	9.604519774
chr7:106,750,321-107,733,654	High Copy Gai	983334	q22.3 - q31.1	0.97037321	15.41218638
chr7:107,463,111-107,791,058	llelic Imbalanc	327948	q31.1	2.90961649	26.31578947
chr7:107,791,058-107,929,465	LOH	138408	q31.1	0	0
chr7:107,733,654-108,062,176	CN Gain	328523	q31.1	0	14.92537313
chr7:107,929,465-108,062,176	llelic Imbalanc	132712	q31.1	0	26.19047619
chr7:108,062,176-108,098,212	High Copy Gai	36037	q31.1	0	0
chr7:108,062,176-108,241,045	LOH	178870	q31.1	0	1.538461538
chr7:108,241,045-108,861,692	llelic Imbalanc	620648	q31.1	0.17368971	30.85714286
chr7:108,861,692-109,102,439	LOH	240748	q31.1	0	2.5
chr7:108,098,212-111,017,921	CN Gain	2919710	q31.1	4.32447206	26.51605232
chr7:109,102,439-111,017,921	llelic Imbalanc	1915483	q31.1	6.53537856	30.79777365
chr7:111,017,921-111,058,551	CN Loss	40631	q31.1	88.3042087	5.263157895
chr7:111,017,921-111,058,551	LOH	40631	q31.1	88.3042087	5.263157895
chr7:111,058,551-111,400,239	llelic Imbalanc	341689	q31.1	17.9538058	24.69135802
chr7:111,400,239-111,629,235	LOH	228997	q31.1	0	1.315789474
chr7:111,629,235-112,212,705	llelic Imbalanc	583471	q31.1	0	27.13567839
chr7:112,212,705-112,823,035	LOH	610331	q31.1	1.15462127	2.649006623
chr7:111,058,551-115,267,861	CN Gain	4209311	q31.1 - q31.2	1.77784482	20.95754291
chr7:112,823,035-115,267,861	llelic Imbalanc	2444827	q31.1 - q31.2	0.26349523	25.5
chr7:115,267,861-115,823,762	LOH	555902	q31.2	1.18600254	9.333333333
chr7:115,267,861-116,381,013	High Copy Gai	1113153	q31.2	1.69518628	17.57925072
chr7:115,823,762-116,381,013	llelic Imbalanc	557252	q31.2	2.20313647	23.85786802
chr7:116,381,013-116,391,581	CN Gain	10569	q31.2	0	0
chr7:116,391,581-116,418,444	llelic Imbalanc	26864	q31.2	0	20
chr7:116,391,581-116,418,444	High Copy Gai	26864	q31.2	0	20
chr7:116,418,444-116,428,610	CN Gain	10167	q31.2	0	0
chr7:116,428,610-116,736,508	High Copy Gai	307899	q31.2	0	9.782608696
chr7:116,736,508-116,783,035	CN Gain	46528	q31.2	0	0
chr7:116,783,035-119,080,302	High Copy Gai	2297268	q31.2 - q31.31	2.18829592	3.630363036
chr7:116,428,610-120,825,768	LOH	4397159	q31.2 - q31.31	2.0792521	3.451327434
chr7:119,080,302-119,378,649	CN Gain	298348	q31.31	5.67627628	0
chr7:120,825,768-121,033,191	llelic Imbalanc	207424	q31.31	0	35.38461538
chr7:121,033,191-123,783,261	LOH	2750071	31.31 - q31.3	0.78183464	8.156028369
chr7:119,378,649-126,506,255	High Copy Gai	7127607	31.31 - q31.3	1.35407316	13.23529412
chr7:123,783,261-125,126,365	llelic Imbalanc	1343105	31.32 - q31.3	0.3791218	23.07692308
chr7:125,126,365-125,869,270	LOH	742906	q31.33	4.37054536	12.95546559
chr7:126,506,255-126,566,728	CN Gain	60474	q31.33	29.3651712	38.46153846
chr7:125,869,270-127,566,511	llelic Imbalanc	1697242	q31.33 - q32.1	2.33602653	23.92086331
chr7:126,566,728-127,900,395	High Copy Gai	1333668	q31.33 - q32.1	0.64941248	19.11764706
chr7:127,566,511-127,900,395	LOH	333885	q32.1	0	9.638554217
chr7:127,900,395-127,959,340	CN Gain	58946	q32.1	0	19.04761905

chr7:127,900,395-128,041,535	llelic Imbalanc	141141	q32.1	0	20.45454545
chr7:127,959,340-127,987,802	High Copy Gai	28463	q32.1	0	22.22222222
chr7:127,987,802-128,041,535	CN Gain	53734	q32.1	0	20
chr7:128,041,535-128,141,040	High Copy Gai	99506	q32.1	0	0
chr7:128,041,535-128,295,904	LOH	254370	q32.1	0	4.166666667
chr7:128,141,040-128,295,904	CN Gain	154865	q32.1	0	14.28571429
chr7:128,295,904-128,409,219	llelic Imbalanc	113316	q32.1	0	18.60465116
chr7:128,295,904-128,409,219	High Copy Gai	113316	q32.1	0	18.60465116
chr7:128,409,219-128,421,425	CN Gain	12207	q32.1	0	0
chr7:128,421,425-128,492,464	High Copy Gai	71040	q32.1	27.4257802	0
chr7:128,409,219-128,565,024	LOH	155806	q32.1	50.5497256	0
chr7:128,492,464-128,565,024	CN Gain	72561	q32.1	81.6923925	0
chr7:128,565,024-128,688,749	High Copy Gai	123726	q32.1	0	17.24137931
chr7:128,565,024-128,751,969	llelic Imbalanc	186946	q32.1	0	19.60784314
chr7:128,688,749-128,751,969	CN Gain	63221	q32.1	0	22.72727273
chr7:128,751,969-128,961,614	High Copy Gai	209646	q32.1	0.7970617	10.52631579
chr7:128,961,614-129,010,091	CN Gain	48478	q32.1	0	11.11111111
chr7:129,010,091-129,418,178	High Copy Gai	408088	q32.1 - q32.2	0	2.325581395
chr7:128,751,969-129,770,908	LOH	1018940	q32.1 - q32.2	0.16399412	5.583756345
chr7:129,418,178-129,490,621	CN Gain	72444	q32.2	0	0
chr7:129,770,908-130,378,939	llelic Imbalanc	608032	q32.2	0	23.80952381
chr7:129,490,621-131,414,267	High Copy Gai	1923647	q32.2 - q32.3	0.22218225	14.80075901
chr7:130,378,939-131,612,492	LOH	1233554	q32.2 - q32.3	0.52506864	8.5995086
chr7:131,414,267-131,435,931	CN Gain	21665	q32.3	0	0
chr7:131,435,931-131,612,492	High Copy Gai	176562	q32.3	1.24772741	1.075268817
chr7:131,612,492-131,751,327	llelic Imbalanc	138836	q32.3	1.0033493	15.2173913
chr7:131,612,492-131,751,327	CN Gain	138836	q32.3	1.0033493	15.2173913
chr7:131,751,327-132,211,238	High Copy Gai	459912	q32.3	1.40788109	11.29032258
chr7:132,211,238-132,240,961	CN Gain	29724	q32.3	0	0
chr7:131,751,327-134,041,248	LOH	2289922	q32.3 - q33	1.4183022	12.53602305
chr7:132,240,961-134,379,636	High Copy Gai	2138676	q32.3 - q33	1.28874186	15.68322981
chr7:134,041,248-134,301,578	llelic Imbalanc	260331	q33	0	32.40740741
chr7:134,301,578-134,466,507	LOH	164930	q33	0.94525523	0
chr7:134,466,507-134,697,077	llelic Imbalanc	230571	q33	0.46233248	22.66666667
chr7:134,697,077-134,924,231	LOH	227155	q33	0	0
chr7:134,924,231-135,040,909	llelic Imbalanc	116679	q33	0	26.02739726
chr7:135,040,909-135,411,436	LOH	370528	q33	0	2.083333333
chr7:135,411,436-136,771,812	llelic Imbalanc	1360377	q33	0.01411375	25.42735043
chr7:136,771,812-136,957,690	LOH	185879	q33	0.67194612	2.857142857
chr7:136,957,690-137,137,291	llelic Imbalanc	179602	q33	0	26.86567164
chr7:137,137,291-137,378,095	LOH	240805	q33	0	0
chr7:137,378,095-141,007,877	llelic Imbalanc	3629783	q33 - q34	13.7814888	23.37246531
chr7:134,379,636-144,759,017	CN Gain	10379382	q33 - q35	22.7143603	20.3960396
chr7:141,007,877-141,414,968	LOH	407092	q34	90.5900646	5.64516129
chr7:141,414,968-142,555,309	llelic Imbalanc	1140342	q34	48.8417938	33.5078534
chr7:142,555,309-142,922,535	LOH	367227	q34	14.3600943	3.448275862
chr7:142,922,535-143,577,078	llelic Imbalanc	654544	q34 - q35	57.7269943	38.38383838

chr7:143,577,078-144,106,033	LOH	528956	q35	94.2492272	0
chr7:144,106,033-144,141,071	llelic Imbalanc	35039	q35	0	52.63157895
chr7:144,141,071-144,377,163	LOH	236093	q35	0	4.04040404
chr7:144,377,163-144,759,017	llelic Imbalanc	381855	q35	0	26.27118644
chr7:144,759,017-144,832,373	High Copy Gai	73357	q35	8.7027646	8
chr7:144,759,017-144,832,373	LOH	73357	q35	8.7027646	8
chr7:144,832,373-146,099,054	llelic Imbalanc	1266682	q35	0.40175861	36.13636364
chr7:144,832,373-146,099,054	CN Gain	1266682	q35	0.40175861	36.13636364
chr7:146,099,054-146,115,152	High Copy Gai	16099	q35	0	0
chr7:146,099,054-146,247,768	LOH	148715	q35	19.2254932	2.197802198
chr7:146,247,768-146,578,919	llelic Imbalanc	331152	q35	2.8847867	27.95698925
chr7:146,578,919-146,759,873	LOH	180955	q35	0	1.234567901
chr7:146,759,873-146,787,828	llelic Imbalanc	27956	q35	0	52
chr7:146,787,828-147,025,925	LOH	238098	q35	0.94457301	2.040816327
chr7:147,025,925-147,874,461	llelic Imbalanc	848537	q35	3.62801343	34.49367089
chr7:146,115,152-149,472,523	CN Gain	3357372	q35 - q36.1	2.43607275	25.06234414
chr7:147,874,461-148,037,974	LOH	163514	q35 - q36.1	3.79297059	1.652892562
chr7:148,037,974-150,245,180	llelic Imbalanc	2207207	q36.1	17.1094587	26.14770459
chr7:149,472,523-149,585,632	High Copy Gai	113110	q36.1	34.4172435	11.76470588
chr7:149,585,632-150,042,589	CN Gain	456958	q36.1	73.1587436	15.55555556
chr7:150,042,589-150,245,180	High Copy Gai	202592	q36.1	0	18.18181818
chr7:150,245,180-150,385,871	LOH	140692	q36.1	11.2075399	5.084745763
chr7:150,385,871-151,042,791	llelic Imbalanc	656921	q36.1	0	43.01075269
chr7:151,042,791-151,371,731	LOH	328941	q36.1	0	2.105263158
chr7:151,371,731-151,677,936	llelic Imbalanc	306206	q36.1	12.3789618	40.24390244
chr7:151,677,936-152,261,694	LOH	583759	q36.1	15.4067268	2.43902439
chr7:152,261,694-155,398,299	llelic Imbalanc	3136606	q36.1 - q36.3	15.9577633	24.62977397
chr7:150,245,180-159,138,663	CN Gain	8893484	q36.1 - q36.3	10.1353092	24.36835106
chr7:155,398,299-155,736,162	LOH	337864	q36.3	1.22090907	6.486486486
chr7:155,736,162-158,954,152	llelic Imbalanc	3217991	q36.3	7.48168888	28.99590164
chr7:158,954,152-159,138,663	LOH	184512	q36.3	6.69553577	3.333333333
chr8:0-6,614,210	LOH	6614211	p23.3 - p23.1	47.3180017	2.170926079
chr8:6,581,994-6,614,210	CN Loss	32217	p23.1	0	10
chr8:6,614,210-6,619,962	llelic Imbalanc	5753	p23.1	0	33.33333333
chr8:6,614,210-6,619,962	CN Gain	5753	p23.1	0	33.33333333
chr8:6,619,962-7,044,071	LOH	424110	p23.1	51.3858466	6.25
chr8:6,825,143-7,044,071	CN Loss	218929	p23.1	98.4912848	14.63414634
chr8:8,093,066-10,003,077	LOH	1910012	p23.1	14.1381908	13.97153946
chr8:10,003,077-14,284,470	llelic Imbalanc	4281394	p23.1 - p22	30.7375894	18.43288375
chr8:14,284,470-14,896,289	LOH	611820	p22	44.9069087	9.019607843
chr8:14,896,289-15,598,809	llelic Imbalanc	702521	p22	100	23.1441048
chr8:15,598,809-16,242,070	LOH	643262	p22	32.5233459	11.15107914
chr8:16,242,070-16,873,346	llelic Imbalanc	631277	p22	11.3476514	23.04147465
chr8:16,873,346-18,009,292	LOH	1135947	p22	28.5900034	14.07129456
chr8:18,009,292-18,521,036	llelic Imbalanc	511745	p22	0.1320973	24.44444444
chr8:18,521,036-19,155,406	LOH	634371	p22 - p21.3	5.54581711	14.23220974
chr8:19,155,406-19,344,676	llelic Imbalanc	189271	p21.3	48.6469065	28.75

chr8:19,344,676-19,607,131	LOH	262456	p21.3	59.7454802	12.82051282
chr8:19,607,131-19,915,897	llelic Imbalanc	308767	p21.3	1.18633528	24.56140351
chr8:19,915,897-20,659,700	LOH	743804	p21.3	0.11387424	11.76470588
chr8:20,659,700-21,723,227	llelic Imbalanc	1063528	p21.3	1.07942723	20.7977208
chr8:21,723,227-24,309,783	LOH	2586557	p21.3 - p21.2	3.24794824	12.77602524
chr8:24,309,783-26,715,271	llelic Imbalanc	2405489	p21.2	2.57536101	20.10582011
chr8:8,093,066-43,778,914	CN Loss	35685849	p23.1 - p11.1	13.848061	16.08527132
chr8:26,715,271-27,524,519	LOH	809249	p21.2 - p21.1	1.67896615	11.78707224
chr8:27,524,519-28,353,626	llelic Imbalanc	829108	p21.1	0.30587126	22.62773723
chr8:28,353,626-38,569,939	LOH	10216314	p21.1 - p11.22	1.21754296	13.17225253
chr8:38,569,939-39,614,622	llelic Imbalanc	1044684	p11.22	27.8137004	20.52631579
chr8:39,614,622-40,633,592	LOH	1018971	11.22 - p11.2	0.86351904	12.97935103
chr8:40,633,592-41,689,070	llelic Imbalanc	1055479	p11.21	0.57310527	23.98523985
chr8:41,689,070-43,397,674	LOH	1708605	p11.21 - p11.1	29.2068262	11.86440678
chr8:43,397,674-43,778,914	llelic Imbalanc	381241	p11.1	100	42.85714286
chr8:46,913,606-49,820,356	LOH	2906751	q11.1 - q11.21	28.7969726	14.39842209
chr8:49,820,356-50,180,661	llelic Imbalanc	360306	q11.21	0.15264845	28.57142857
chr8:50,180,661-51,958,349	LOH	1777689	q11.21	1.58852397	10.83743842
chr8:51,958,349-60,201,548	llelic Imbalanc	8243200	q11.21 - q12.1	3.01494602	17.97255423
chr8:46,913,606-67,333,426	CN Loss	20419821	q11.1 - q13.1	5.77076096	16.11513609
chr8:60,201,548-66,251,311	LOH	6049764	q12.1 - q13.1	1.02734603	14.97157296
chr8:66,251,311-66,387,765	llelic Imbalanc	136455	q13.1	0.13630967	50
chr8:66,387,765-67,333,426	LOH	945662	q13.1	0.17638456	7.511737089
chr8:71,013,812-71,621,688	CN Gain	607877	q13.3	0.1205838	6.666666667
chr8:71,013,812-72,012,455	LOH	998644	q13.3	0.34556894	5.445544554
chr8:72,347,846-72,597,665	LOH	249820	q13.3	0	1.204819277
chr8:73,489,340-73,743,268	LOH	253929	q13.3	0	1.515151515
chr8:74,373,012-75,026,305	LOH	653294	q21.11	0.02694044	3.184713376
chr8:76,701,124-77,031,027	LOH	329904	q21.11	0	2.702702703
chr8:77,845,259-78,446,940	LOH	601682	21.11 - q21.1	1.2785845	0.694444444
chr8:81,199,412-81,798,231	LOH	598820	q21.13	0.07848782	5.882352941
chr8:84,914,165-85,468,041	LOH	553877	q21.2	2.20500617	0
chr8:87,004,336-87,038,042	llelic Imbalanc	33707	q21.3	0	66.66666667
chr8:87,038,042-87,676,256	LOH	638215	q21.3	2.62278797	2.75862069
chr8:87,676,256-88,300,583	llelic Imbalanc	624328	q21.3	0	37.42331288
chr8:88,300,583-89,036,469	LOH	735887	q21.3	0.91223369	0
chr8:89,036,469-93,627,620	llelic Imbalanc	4591152	q21.3 - q22.1	0.22140418	31.10687023
chr8:93,627,620-94,087,515	LOH	459896	q22.1	1.23745638	1.052631579
chr8:94,087,515-95,284,812	llelic Imbalanc	1197298	q22.1	0.50747642	46.03174603
chr8:95,284,812-95,583,469	LOH	298658	q22.1	1.01353727	0
chr8:95,583,469-98,812,103	llelic Imbalanc	3228635	q22.1	0.72780005	34.1986456
chr8:98,812,103-99,090,951	LOH	278849	q22.1 - q22.2	0.44289362	1.234567901
chr8:99,090,951-99,847,235	llelic Imbalanc	756285	q22.2	0.29089601	32.0754717
chr8:99,847,235-100,827,605	LOH	980371	q22.2	0.35700807	2.976190476
chr8:100,827,605-101,978,285	llelic Imbalanc	1150681	q22.2 - q22.3	0	44.19475655
chr8:101,978,285-102,458,447	LOH	480163	q22.3	5.74493608	1.652892562
chr8:102,458,447-102,554,225	llelic Imbalanc	95779	q22.3	0	51.72413793

chr8:102,554,225-102,749,503	LOH	195279	q22.3	4.42036481	1.612903226
chr8:102,749,503-106,775,212	llelic Imbalanc	4025710	q22.3 - q23.1	0.8640714	30.12704174
chr8:87,004,336-123,204,084	CN Gain	36199749	q21.3 - q24.13	1.26608892	25.05344164
chr8:106,775,212-107,098,118	LOH	322907	q23.1	0.72188191	2.272727273
chr8:107,098,118-108,792,201	llelic Imbalanc	1694084	q23.1	2.78970983	29.35606061
chr8:108,792,201-109,609,421	LOH	817221	q23.1	0.29000759	0.490196078
chr8:109,609,421-109,725,651	llelic Imbalanc	116231	q23.1	0	50
chr8:109,725,651-109,996,621	LOH	270971	q23.1	0	0
chr8:109,996,621-111,277,577	llelic Imbalanc	1280957	q23.1 - q23.2	3.36920238	45.54455446
chr8:111,277,577-111,705,379	LOH	427803	q23.2	0	2.127659574
chr8:111,705,379-111,980,571	llelic Imbalanc	275193	q23.2	1.52838745	27.5862069
chr8:111,980,571-112,376,514	LOH	395944	q23.2 - q23.3	0.67913816	0
chr8:112,376,514-113,893,779	llelic Imbalanc	1517266	q23.3	0.77112436	44.6685879
chr8:113,893,779-114,245,065	LOH	351287	q23.3	2.33712701	0
chr8:114,245,065-115,239,824	llelic Imbalanc	994760	q23.3	2.56343496	30.95238095
chr8:115,239,824-116,361,612	LOH	1121789	q23.3	4.30241721	1.465201465
chr8:116,361,612-118,859,354	llelic Imbalanc	2497743	q23.3 - q24.11	0.8972504	28.34224599
chr8:118,859,354-119,303,358	LOH	444005	24.11 - q24.1	0.3524743	1.485148515
chr8:119,303,358-120,217,845	llelic Imbalanc	914488	q24.12	2.14743348	29.09698997
chr8:120,217,845-120,503,385	LOH	285541	q24.12	1.00861526	2.5
chr8:120,503,385-120,611,133	llelic Imbalanc	107749	q24.12	0	50
chr8:124,338,573-124,498,672	CN Gain	160100	q24.13	0	0
chr8:125,352,690-125,448,672	CN Gain	95983	q24.13	0	12
chr8:126,287,578-126,331,086	CN Gain	43509	q24.13	0	0
chr8:121,415,006-133,084,804	LOH	11669799	24.12 - q24.2	1.87747037	3.671830178
chr8:133,084,804-135,637,988	llelic Imbalanc	2553185	q24.22	1.13615	33.40448239
chr8:135,637,988-135,909,801	LOH	271814	q24.22	28.4956937	3.797468354
chr8:135,909,801-136,065,830	llelic Imbalanc	156030	q24.22	24.7562953	41.07142857
chr8:136,065,830-136,366,533	LOH	300704	q24.22	0.7229725	4
chr8:136,366,533-138,699,234	llelic Imbalanc	2332702	24.22 - q24.2	12.1702696	28.40466926
chr8:138,699,234-138,933,553	LOH	234320	q24.23	3.34245196	2.105263158
chr8:133,084,804-144,805,916	CN Loss	11721113	q24.22 - q24.3	5.31457254	26.27024009
chr8:138,933,553-144,002,597	llelic Imbalanc	5069045	q24.23 - q24.3	1.86023242	25.05105514
chr8:144,002,597-144,805,916	LOH	803320	q24.3	11.1585808	10
chr8:145,409,285-146,177,729	LOH	768445	q24.3	24.0746235	2.666666667
chr8:145,409,285-146,364,022	CN Loss	954738	q24.3	25.2705195	12.37113402
chr8:146,177,729-146,364,022	llelic Imbalanc	186294	q24.3	30.2034966	45.45454545
chr9:334,185-407,987	LOH	73803	p24.3	13.1459852	3.448275862
chr9:1,667,800-1,816,337	LOH	148538	p24.3	0	0.854700855
chr9:4,171,957-4,304,216	LOH	132260	p24.2	0	0
chr9:4,970,315-5,270,500	LOH	300186	p24.1	2.44449256	0
chr9:6,995,657-7,136,922	LOH	141266	p24.1	0	2.380952381
chr9:10,774,850-10,840,032	CN Gain	65183	p23	0	68
chr9:11,259,163-11,825,989	LOH	566827	p23	0.57054546	1.538461538
chr9:12,019,585-12,068,285	CN Gain	48701	p23	100	23.07692308
chr9:12,768,844-12,995,714	LOH	226871	p23	0	4
chr9:12,995,714-13,116,847	CN Gain	121134	p23	3.2451933	59.375

chr9:13,509,172-13,743,395	LOH	234224	p23	2.00108444	3.921568627
chr9:15,033,268-15,280,992	LOH	247725	p22.3	0.77465244	0
chr9:15,371,253-16,071,117	LOH	699865	p22.3	1.16808408	3.157894737
chr9:16,366,928-16,825,953	LOH	459026	p22.3 - p22.2	0	2.793296089
chr9:16,908,436-16,971,466	CN Gain	63031	p22.2	0	38.46153846
chr9:19,149,418-19,190,330	CN Gain	40913	p22.1	0	69.23076923
chr9:22,548,184-22,864,321	LOH	316138	p21.3	0.38021491	1.298701299
chr9:24,134,061-24,749,331	LOH	615271	p21.3	5.14408309	3.846153846
chr9:24,502,828-24,517,948	CN Loss	15121	p21.3	100	0
chr9:26,930,422-27,166,813	llelic Imbalanc	236392	p21.2	80.9108638	42.25352113
chr9:27,166,813-27,467,628	LOH	300816	p21.2	0	1.503759398
chr9:26,930,422-29,445,780	CN Gain	2515359	p21.2 - p21.1	16.9196194	28.96551724
chr9:27,467,628-29,445,780	llelic Imbalanc	1978153	p21.2 - p21.1	11.8455508	33.03303303
chr9:29,897,864-30,940,786	LOH	1042923	p21.1	22.7544342	4.580152672
chr9:30,940,786-31,561,904	llelic Imbalanc	621119	p21.1	0.169372	56.28140704
chr9:31,561,904-31,853,182	LOH	291279	p21.1	0	1.123595506
chr9:30,940,786-40,784,142	CN Gain	9843357	p21.1 - p13.1	29.2083107	32.07930859
chr9:31,853,182-40,784,142	llelic Imbalanc	8930961	p21.1 - p13.1	32.1804823	30.85169744
chr9:44,136,675-44,775,384	LOH	638710	p11.2	100	0
chr9:71,647,392-71,871,533	LOH	224142	q21.11	2.67376339	1.388888889
chr9:74,397,315-74,907,594	LOH	510280	q21.13	1.01748259	4.201680672
chr9:75,705,829-76,723,666	LOH	1017838	q21.13	1.61725306	3.896103896
chr9:77,792,433-78,142,003	LOH	349571	q21.13	2.98795663	1.834862385
chr9:79,755,409-79,795,643	CN Gain	40235	q21.2	50.0720783	52.38095238
chr9:80,134,734-80,750,824	LOH	616091	q21.2	0	2.469135802
chr9:83,283,603-83,700,972	LOH	417370	q21.31	1.30675733	2.395209581
chr9:84,483,599-85,167,286	LOH	683688	q21.32	5.70992282	6.837606838
chr9:89,441,642-89,672,827	LOH	231186	q21.33	0	0
chr9:90,133,900-90,262,336	LOH	128437	q21.33	0	0
chr9:92,686,100-92,866,310	LOH	180211	q22.2	0.41840075	0
chr9:93,101,353-93,327,100	LOH	225748	q22.2	0	0
chr9:93,440,348-93,620,522	LOH	180175	q22.2	0	1.5625
chr9:94,646,650-95,630,502	LOH	983853	q22.31	0	4.926108374
chr9:96,356,100-96,627,211	LOH	271112	22.31 - q22.3	2.48422233	1.25
chr9:99,263,859-100,277,555	LOH	1013697	22.32 - q22.3	3.70564745	4.736842105
chr9:101,742,310-102,211,067	LOH	468758	q22.33	0	5.660377358
chr9:103,873,830-104,118,999	LOH	245170	q31.1	0	5.494505495
chr9:104,118,999-104,258,479	llelic Imbalanc	139481	q31.1	0.98795526	36.84210526
chr9:104,118,999-104,258,479	CN Gain	139481	q31.1	0.98795526	36.84210526
chr9:106,401,175-106,428,126	CN Loss	26952	q31.1	80.4534155	0
chr9:106,401,175-106,428,126	LOH	26952	q31.1	80.4534155	0
chr9:107,949,391-108,448,494	LOH	499104	q31.1 - q31.2	0.19595154	0.847457627
chr9:114,951,531-115,405,866	LOH	454336	q32	13.7779392	4
chr9:122,140,818-122,433,755	LOH	292938	q33.1	4.50472286	3.333333333
chr9:126,372,765-126,873,596	LOH	500832	q33.3	0.13697235	1.652892562
chr9:127,174,222-128,154,088	LOH	979867	q33.3	4.85790914	4.736842105
chr9:129,057,198-129,265,373	LOH	208176	q33.3	3.13966615	1.612903226

chr9:129,923,196-130,311,119	LOH	387924	q33.3 - q34.11	1.7315292	1.136363636
chr9:135,807,480-135,814,598	CN Gain	7119	q34.13	0	
chr9:140,805,714-141,213,431	LOH	407718	q34.3	44.4161023	0
chr10:0-1,343,000	LOH	1343001	p15.3	12.3125838	3.481012658
chr10:9,589,287-10,093,207	LOH	503921	p14	0	3.731343284
chr10:10,801,920-11,024,678	LOH	222759	p14	0	0
chr10:14,160,605-14,411,054	LOH	250450	p13	0	1.098901099
chr10:18,995,765-19,190,111	LOH	194347	p12.31	0	2.380952381
chr10:25,200,147-25,555,199	LOH	355053	p12.1	1.13166522	3.529411765
chr10:27,965,140-28,400,719	LOH	435580	p12.1	0	3.125
chr10:30,820,420-36,344,220	LOH	5523801	11.23 - p11.2	1.11381658	2.069425901
chr10:36,925,676-37,243,133	LOH	317458	p11.21	0	2.53164557
chr10:37,973,487-38,987,169	LOH	1013683	p11.21 - p11.1	11.7558564	7.913669065
chr10:43,349,072-52,831,007	llelic Imbalanc	9481936	11.21 - q11.2	41.0815619	17.40595171
chr10:43,349,072-56,792,524	CN Loss	13443453	q11.21 - q21.1	31.9232516	14.79028698
chr10:52,831,007-56,792,524	LOH	3961518	q11.23 - q21.1	10.0027338	11.43884892
chr10:56,792,524-57,179,639	CN Gain	387116	q21.1	23.2036992	41.09589041
chr10:57,656,271-57,726,852	CN Gain	70582	q21.1	0	90.90909091
chr10:57,793,596-59,317,593	LOH	1523998	q21.1	19.2151953	1.502145923
chr10:58,092,315-62,652,986	CN Gain	4560672	q21.1 - q21.2	7.06207486	23.72025955
chr10:60,782,318-61,386,562	LOH	604245	q21.1 - q21.2	0.78792673	1.19047619
chr10:61,841,560-61,957,040	LOH	115481	q21.2	0	0
chr10:62,662,552-63,093,306	LOH	430755	q21.2	0	4.04040404
chr10:62,957,960-63,093,306	CN Gain	135347	q21.2	0	5.714285714
chr10:64,184,671-64,862,259	llelic Imbalanc	677589	q21.2 - q21.3	3.46715113	27.43362832
chr10:64,862,259-65,370,434	LOH	508176	q21.3	0	3.191489362
chr10:64,184,671-66,849,882	CN Gain	2665212	q21.2 - q21.3	1.2015559	23.64945978
chr10:65,370,434-65,873,455	llelic Imbalanc	503022	q21.3	0.37473585	46
chr10:65,873,455-66,303,208	LOH	429754	q21.3	0.1731227	1.111111111
chr10:66,303,208-66,508,995	llelic Imbalanc	205788	q21.3	2.62990374	41.09589041
chr10:66,508,995-66,849,882	LOH	340888	q21.3	0.14374265	1.149425287
chr10:66,849,882-67,800,512	llelic Imbalanc	950631	q21.3	7.42297213	18.27586207
chr10:67,800,512-70,252,798	LOH	2452287	q21.3	2.10411836	10.23738872
chr10:70,252,798-71,517,553	llelic Imbalanc	1264756	q21.3 - q22.1	21.2362869	21.78217822
chr10:71,517,553-80,529,082	LOH	9011530	q22.1 - q22.3	14.6487461	13.46240432
chr10:80,529,082-82,442,373	llelic Imbalanc	1913292	q22.3 - q23.1	2.95726055	23.23943662
chr10:82,442,373-83,975,844	LOH	1533472	q23.1	0.91759153	13.11188811
chr10:83,975,844-84,333,915	llelic Imbalanc	358072	q23.1	0.94813598	25.30864198
chr10:84,333,915-84,622,372	LOH	288458	q23.1	9.87460869	10.45751634
chr10:84,622,372-85,604,254	llelic Imbalanc	981883	q23.1	4.43688753	23.07692308
chr10:85,604,254-86,369,362	LOH	765109	q23.1	0	9.417040359
chr10:86,369,362-86,936,548	llelic Imbalanc	567187	q23.1	0	26.94610778
chr10:86,936,548-87,543,190	LOH	606643	q23.1	0.21083275	6.808510638
chr10:87,543,190-95,063,150	llelic Imbalanc	7519961	q23.1 - q23.33	8.03327411	19.46778711
chr10:95,063,150-96,625,170	LOH	1562021	q23.33	0.09001165	12.25
chr10:96,625,170-97,635,354	llelic Imbalanc	1010185	q23.33 - q24.1	21.6544709	25.0965251
chr10:66,849,882-135,534,747	CN Loss	68684866	q21.3 - q26.3	6.41616024	16.32502891

chr10:97,635,354-106,591,257	LOH	8955904	q24.1 - q25.1	0.77062023	14.01869159
chr10:106,591,257-107,036,122	llelic Imbalanc	444866	q25.1	0	33.33333333
chr10:107,036,122-107,608,601	LOH	572480	q25.1	1.88461061	10.38961039
chr10:107,608,601-108,334,996	llelic Imbalanc	726396	q25.1	0.70361167	21.00840336
chr10:108,334,996-109,502,562	LOH	1167567	q25.1	0	11.68831169
chr10:109,502,562-112,645,306	llelic Imbalanc	3142745	q25.1 - q25.2	0.4283836	20.46242775
chr10:112,645,306-124,964,340	LOH	12319035	q25.2 - q26.13	2.23217178	14.16320885
chr10:124,964,340-125,955,839	llelic Imbalanc	991500	q26.13	10.6639543	26.66666667
chr10:125,955,839-127,941,465	LOH	1985627	q26.13 - q26.2	2.9844996	13.22463768
chr10:127,941,465-128,536,882	llelic Imbalanc	595418	q26.2	0	24.3902439
chr10:128,536,882-129,385,459	LOH	848578	q26.2	0.99154231	12.90322581
chr10:129,385,459-132,380,197	llelic Imbalanc	2994739	q26.2 - q26.3	20.1649694	19.64809384
chr10:132,380,197-133,139,028	LOH	758832	q26.3	1.09919073	11.8705036
chr10:133,139,028-135,178,835	llelic Imbalanc	2039808	q26.3	10.7713132	22.33766234
chr10:135,178,835-135,534,747	LOH	355913	q26.3	97.1481715	9.523809524
chr11:15,300,142-15,471,122	LOH	170981	p15.2	0	0
chr11:15,624,001-15,922,685	LOH	298685	p15.2	0	0
chr11:15,960,504-16,389,277	LOH	428774	p15.2 - p15.1	0	3.496503497
chr11:18,816,250-19,055,716	LOH	239467	p15.1	30.9396741	1.869158879
chr11:18,940,853-18,962,001	CN Gain	21149	p15.1	100	0
chr11:20,746,007-20,853,982	CN Gain	107976	p15.1	5.53276221	46.15384615
chr11:20,874,347-21,018,750	LOH	144404	p15.1	0	0
chr11:21,686,332-21,993,048	LOH	306717	p15.1 - p14.3	3.53486613	1.550387597
chr11:23,022,663-23,051,805	llelic Imbalanc	29143	p14.3	0	20
chr11:23,022,663-23,051,805	CN Loss	29143	p14.3	0	20
chr11:24,406,453-24,668,790	LOH	262338	p14.3	5.79559879	3.669724771
chr11:24,812,000-25,249,034	LOH	437035	p14.3	100	5.172413793
chr11:25,608,209-25,631,096	CN Loss	22888	p14.3	100	0
chr11:25,608,209-25,631,096	LOH	22888	p14.3	100	0
chr11:26,129,516-26,550,805	LOH	421290	p14.2	0	4.545454545
chr11:27,984,196-28,990,144	LOH	1005949	p14.1	0	4.977375566
chr11:31,308,280-31,812,203	LOH	503924	p13	0.82056187	7.476635514
chr11:32,289,972-32,592,123	LOH	302152	p13	0	2.919708029
chr11:34,819,884-35,101,447	LOH	281564	p13	0	3.205128205
chr11:36,412,830-36,736,856	LOH	324027	p12	4.34316999	2.884615385
chr11:36,895,052-37,080,664	LOH	185613	p12	0	0
chr11:39,424,273-39,626,163	CN Loss	201891	p12	0	17.30769231
chr11:39,424,273-42,130,700	llelic Imbalanc	2706428	p12	0.72253935	30.29350105
chr11:41,347,655-41,414,764	CN Loss	67110	p12	0.48577687	38.46153846
chr11:41,821,963-41,850,534	CN Loss	28572	p12	0	20
chr11:42,238,170-42,782,647	LOH	544478	p12	0	0
chr11:43,589,903-43,880,679	LOH	290777	p11.2	0	1.428571429
chr11:45,086,168-45,571,104	LOH	484937	p11.2	0.47903228	0
chr11:46,172,699-47,332,808	LOH	1160110	p11.2	0.90388058	1.503759398
chr11:49,553,512-51,189,484	LOH	1635973	p11.12	70.4238826	1.898734177
chr11:55,263,656-55,360,464	LOH	96809	q11	25.3966614	0
chr11:55,360,464-55,452,585	llelic Imbalanc	92122	q11	100	66.66666667

chr11:55,360,464-55,452,585	CN Gain	92122	q11	100	66.66666667
chr11:59,036,569-59,799,967	LOH	763399	q12.1	0	2.487562189
chr11:60,833,699-61,710,164	LOH	876466	q12.2 - q12.3	7.51165192	1.769911504
chr11:72,265,646-74,131,371	LOH	1865726	q13.4	0.34297659	4.945054945
chr11:75,201,148-75,904,388	LOH	703241	q13.5	0.35848359	1.973684211
chr11:77,877,779-78,431,642	LOH	553864	q14.1	10.5811365	2.649006623
chr11:79,197,395-79,381,314	LOH	183920	q14.1	0	1.030927835
chr11:82,050,518-82,333,942	LOH	283425	q14.1	0.04727899	0
chr11:83,959,234-84,275,074	LOH	315841	q14.1	0	2.325581395
chr11:84,379,065-85,533,621	LOH	1154557	q14.1	2.32037251	3.680981595
chr11:86,850,767-87,587,313	LOH	736547	q14.2	0	1.374570447
chr11:88,819,207-89,197,637	LOH	378431	q14.3	100	3.658536585
chr11:90,401,145-90,634,633	LOH	233489	q14.3	1.94999315	0
chr11:90,922,781-91,238,342	LOH	315562	q14.3	0	3.157894737
chr11:93,544,286-93,781,124	LOH	236839	q21	16.3512612	1.265822785
chr11:94,463,124-94,678,211	LOH	215088	q21	0	2.702702703
chr11:95,383,675-95,802,324	LOH	418650	q21	5.46018264	2
chr11:95,979,731-96,984,366	LOH	1004636	q21	6.70482314	2.536231884
chr11:97,668,025-97,902,827	LOH	234803	q22.1	0	2.222222222
chr11:99,600,547-100,080,135	LOH	479589	q22.1	1.65058342	3.645833333
chr11:102,424,560-102,635,384	LOH	210825	q22.2	8.3733351	2.222222222
chr11:104,194,326-104,781,033	LOH	586708	q22.3	2.68907649	3.723404255
chr11:105,060,065-105,482,151	LOH	422087	q22.3	3.45308776	0
chr11:105,920,719-106,262,352	LOH	341634	q22.3	0	0
chr11:116,575,783-116,973,432	LOH	397650	q23.3	1.96228332	3.333333333
chr11:120,976,420-121,235,400	LOH	258981	q23.3 - q24.1	0.46181172	1.869158879
chr11:126,057,475-126,312,237	LOH	254763	q24.2	6.82597876	0
chr11:128,769,409-129,200,324	LOH	430916	q24.3	0	2.75862069
chr11:133,140,740-133,394,296	LOH	253557	q25	6.81111865	1.834862385
chr11:134,415,528-134,612,095	LOH	196568	q25	100	0
chr11:134,538,927-135,006,516	CN Gain	467590	q25	53.0698969	36.52173913
chr11:134,612,095-135,006,516	llelic Imbalanc	394422	q25	44.3640171	45.40540541
chr12:1,947,360-1,985,768	llelic Imbalanc	38409	p13.33	53.0983129	12.5
chr12:1,947,360-1,985,768	CN Loss	38409	p13.33	53.0983129	12.5
chr12:2,354,870-2,540,995	LOH	186126	p13.33	2.79059772	1.428571429
chr12:5,593,755-5,883,076	LOH	289322	p13.31	6.6804691	4.379562044
chr12:7,816,810-8,007,890	CN Gain	191081	p13.31	57.0206196	34.375
chr12:8,007,890-8,840,289	LOH	832400	p13.31	54.8595085	5.844155844
chr12:9,488,462-9,917,031	LOH	428570	p13.31	56.6104875	1.785714286
chr12:10,493,225-10,776,726	LOH	283502	p13.2	15.8800145	2.083333333
chr12:13,349,783-13,380,609	CN Gain	30827	p13.1	0	61.53846154
chr12:15,348,107-15,636,635	LOH	288529	p12.3	2.31901237	0
chr12:20,986,031-21,312,468	LOH	326438	p12.2 - p12.1	0	0
chr12:22,374,442-22,802,013	LOH	427572	p12.1	4.51527349	3.80952381
chr12:22,802,013-22,897,051	CN Gain	95039	p12.1	0	47.36842105
chr12:23,118,580-23,685,464	LOH	566885	p12.1	0	2.222222222
chr12:24,988,098-25,003,259	CN Gain	15162	p12.1	0	0

chr12:30,377,530-30,405,797	CN Gain	28268	p11.22	0	39.13043478
chr12:31,062,885-31,087,944	CN Gain	25060	p11.21	0	20
chr12:31,495,452-31,570,817	CN Gain	75366	p11.21	24.2367147	20
chr12:31,570,817-32,039,715	LOH	468899	p11.21	14.3896967	1.550387597
chr12:31,935,457-32,076,822	CN Gain	141366	p11.21	65.4631627	32.14285714
chr12:32,187,996-32,305,794	CN Gain	117799	p11.21	0	43.18181818
chr12:32,393,754-32,647,763	CN Gain	254010	p11.21	0.24251109	35.95505618
chr12:32,778,865-33,168,698	CN Gain	389834	p11.21	1.10637119	30.90909091
chr12:33,369,498-33,575,526	CN Gain	206029	p11.1	32.9202827	32.8358209
chr12:33,575,526-33,759,173	LOH	183648	p11.1	100	6.25
chr12:33,759,173-33,857,310	CN Gain	98138	p11.1	100	35.29411765
chr12:38,661,350-39,428,755	LOH	767406	q12	1.11818401	2.487562189
chr12:43,540,024-44,049,545	LOH	509522	q12	0	0.751879699
chr12:44,204,543-44,982,173	LOH	777631	q12	0.17193267	3.278688525
chr12:45,659,092-45,942,394	LOH	283303	q12	2.34908331	0
chr12:48,313,138-49,003,146	LOH	690009	q13.11	0.34622787	1.485148515
chr12:51,134,447-51,595,489	LOH	461043	13.12 - q13.1	58.6497543	1.030927835
chr12:51,911,432-52,286,703	LOH	375272	q13.13	0.38398917	1.492537313
chr12:56,106,766-57,236,619	LOH	1129854	q13.2 - q13.3	0.41633735	4.06504065
chr12:58,738,574-58,944,983	LOH	206410	q14.1	0	0
chr12:60,469,872-60,724,080	LOH	254209	q14.1	1.2953959	0
chr12:69,817,304-70,113,823	LOH	296520	q15	0	1.98019802
chr12:71,713,517-72,344,552	LOH	631036	q21.1	1.49167637	4.142011834
chr12:73,024,089-74,172,527	LOH	1148439	q21.1	0.16274279	0.716845878
chr12:76,522,384-76,583,054	CN Gain	60671	q21.2	0	0
chr12:76,522,384-76,583,054	LOH	60671	q21.2	0	0
chr12:78,573,703-78,920,648	LOH	346946	q21.2	0	0.952380952
chr12:79,727,665-80,879,123	LOH	1151459	q21.2 - q21.31	1.98661176	1.908396947
chr12:82,586,008-83,160,625	LOH	574618	q21.31	0.01879513	2.678571429
chr12:84,919,238-85,578,070	llelic Imbalanc	658833	q21.31	0.88171188	26.99386503
chr12:84,919,238-85,789,602	CN Gain	870365	q21.31	0.66742191	22.56410256
chr12:85,578,070-85,789,602	LOH	211533	q21.31	0	0
chr12:88,346,018-89,248,259	LOH	902242	21.32 - q21.3	1.89949249	2.608695652
chr12:90,271,029-91,066,088	LOH	795060	q21.33	2.08701493	2.717391304
chr12:91,318,001-91,735,182	LOH	417182	q21.33	0.78575007	3.305785124
chr12:92,864,577-93,624,241	LOH	759665	q22	0.24194907	1.363636364
chr12:94,497,941-94,665,800	LOH	167860	q22	0	1.298701299
chr12:95,357,992-95,730,328	LOH	372337	q22	0.901605	2.247191011
chr12:96,167,945-96,364,039	LOH	196095	q22 - q23.1	0	0
chr12:97,801,934-98,060,577	LOH	258644	q23.1	0.58768264	7.954545455
chr12:98,060,577-98,101,401	llelic Imbalanc	40825	q23.1	0	36.36363636
chr12:98,060,577-98,101,401	CN Gain	40825	q23.1	0	36.36363636
chr12:99,244,826-99,576,191	LOH	331366	q23.1	0	2.173913043
chr12:100,301,849-100,943,815	LOH	641967	q23.1	0	2.459016393
chr12:105,444,129-105,816,277	LOH	372149	q23.3	0.20905661	4.201680672
chr12:108,179,345-108,685,829	LOH	506485	q23.3	0.48550398	6.25
chr12:108,897,705-109,251,913	LOH	354209	q23.3 - q24.11	0.112928	1.388888889

chr12:109,251,913-109,330,555	llelic Imbalanc	78643	q24.11	0	59.25925926
chr12:109,251,913-109,330,555	CN Gain	78643	q24.11	0	59.25925926
chr12:109,641,791-109,779,137	llelic Imbalanc	137347	q24.11	0	42.85714286
chr12:109,641,791-109,779,137	CN Gain	137347	q24.11	0	42.85714286
chr12:110,998,179-111,428,255	llelic Imbalanc	430077	q24.11	6.57325682	50
chr12:111,428,255-113,264,845	LOH	1836591	24.11 - q24.1	0.23908439	1.557632399
chr12:113,264,845-121,015,469	llelic Imbalanc	7750625	24.13 - q24.3	1.08476427	31.83505155
chr12:110,998,179-123,930,213	CN Gain	12932035	24.11 - q24.3	3.38652837	27.41984733
chr12:121,015,469-121,680,436	LOH	664968	q24.31	0.90636077	5.333333333
chr12:121,680,436-123,364,762	llelic Imbalanc	1684327	q24.31	5.03358613	28.33333333
chr12:124,124,873-124,182,474	CN Gain	57602	q24.31	0	0
chr12:123,364,762-133,304,566	LOH	9939805	24.31 - q24.3	11.1054101	5.001623904
chr12:129,942,348-129,978,654	CN Gain	36307	q24.33	0	0
chr12:133,304,566-133,309,594	CN Loss	5029	q24.33	20.6046142	
chr12:133,309,594-133,851,895	LOH	542302	q24.33	16.2170086	9.589041096
chr13:19,436,287-19,724,789	llelic Imbalanc	288503	q11 - q12.11	10.2543483	25.26315789
chr13:19,724,789-20,757,287	LOH	1032499	q12.11	2.36106995	9.486166008
chr13:20,757,287-22,375,710	llelic Imbalanc	1618424	q12.11	1.9340432	19.36842105
chr13:22,375,710-23,293,668	LOH	917959	q12.11	1.64811462	13.54466859
chr13:23,293,668-24,094,461	llelic Imbalanc	800794	12.11 - q12.1	3.45320201	22.10144928
chr13:24,094,461-27,902,446	LOH	3807986	q12.12 - q12.2	1.55289477	14.91935484
chr13:27,902,446-29,538,615	llelic Imbalanc	1636170	q12.2 - q12.3	16.985287	18.18181818
chr13:29,538,615-30,156,974	LOH	618360	q12.3	2.28394832	12.39669421
chr13:30,156,974-31,655,126	llelic Imbalanc	1498153	q12.3	0.01735471	21.78988327
chr13:31,655,126-32,329,199	LOH	674074	q12.3 - q13.1	0.18202776	12.39316239
chr13:32,329,199-32,663,244	llelic Imbalanc	334046	q13.1	2.44966995	25.3968254
chr13:32,663,244-32,924,007	LOH	260764	q13.1	0.52691524	14.52991453
chr13:32,924,007-34,173,398	llelic Imbalanc	1249392	q13.1 - q13.2	1.03290323	22.50639386
chr13:19,436,287-48,706,278	CN Loss	29269992	q11 - q14.2	3.69643093	16.71775783
chr13:34,173,398-36,534,681	LOH	2361284	q13.2 - q13.3	2.29375301	13.55463347
chr13:36,534,681-36,818,971	llelic Imbalanc	284291	q13.3	1.24591087	27.18446602
chr13:36,818,971-38,222,865	LOH	1403895	q13.3	28.1650894	12.6984127
chr13:38,222,865-39,702,497	llelic Imbalanc	1479633	q13.3	4.04925008	21.80293501
chr13:39,702,497-42,770,535	LOH	3068039	q13.3 - q14.11	1.00419226	12.51448436
chr13:42,770,535-44,143,984	llelic Imbalanc	1373450	q14.11	1.4149051	22.22222222
chr13:44,143,984-46,080,950	LOH	1936967	14.11 - q14.1	0.78772679	10.80550098
chr13:46,080,950-47,568,578	llelic Imbalanc	1487629	q14.13 - q14.2	0	23.41920375
chr13:47,568,578-48,690,851	LOH	1122274	q14.2	0.02316727	12.42038217
chr13:48,690,851-48,706,278	llelic Imbalanc	15428	q14.2	0	66.66666667
chr13:48,706,278-48,742,219	ozygous Copy	35942	q14.2	0	73.33333333
chr13:48,742,219-48,785,979	llelic Imbalanc	43761	q14.2	9.99771481	100
chr13:48,742,219-48,785,979	CN Loss	43761	q14.2	9.99771481	100
chr13:48,785,979-48,872,318	ozygous Copy	86340	q14.2	0	75
chr13:48,872,318-48,885,369	llelic Imbalanc	13052	q14.2	0	100
chr13:48,872,318-48,885,369	CN Loss	13052	q14.2	0	100
chr13:48,885,369-48,887,650	ozygous Copy	2282	q14.2	0	100
chr13:48,887,650-48,898,298	CN Loss	10649	q14.2	0	

chr13:48,898,298-48,992,583	ozygous Copy	94286	q14.2	0	61.11111111
chr13:48,992,583-48,998,167	CN Loss	5585	q14.2	0	100
chr13:48,998,167-49,016,181	ozygous Copy	18015	q14.2	0	100
chr13:49,016,181-49,022,512	CN Loss	6332	q14.2	0	100
chr13:49,022,512-49,068,958	ozygous Copy	46447	q14.2	0	66.66666667
chr13:49,068,958-51,624,158	llelic Imbalanc	2555201	q14.2 - q14.3	0.90501722	17.48031496
chr13:51,624,158-52,440,427	LOH	816270	q14.3	0.3448618	9.5
chr13:52,440,427-54,569,506	llelic Imbalanc	2129080	q14.3	6.99405705	22.92490119
chr13:54,569,506-55,174,546	LOH	605041	q14.3	0	7.878787879
chr13:55,174,546-57,588,848	llelic Imbalanc	2414303	q14.3 - q21.1	0.13527719	23.6328125
chr13:57,588,848-60,239,728	LOH	2650881	q21.1 - q21.2	10.0871032	14.48863636
chr13:60,239,728-61,201,244	llelic Imbalanc	961517	q21.2	0	22.11981567
chr13:61,201,244-63,376,256	LOH	2175013	q21.2 - q21.31	1.26160223	13.18493151
chr13:63,376,256-65,865,996	llelic Imbalanc	2489741	21.31 - q21.3	6.13907476	20.12678288
chr13:65,865,996-67,127,027	LOH	1261032	q21.32	2.63736577	14.28571429
chr13:67,127,027-72,183,278	llelic Imbalanc	5056252	21.32 - q21.3	5.16540813	21.04918033
chr13:72,183,278-75,754,778	LOH	3571501	q21.33 - q22.2	3.67207056	12.94838145
chr13:75,754,778-76,272,857	llelic Imbalanc	518080	q22.2	2.62778457	28.07017544
chr13:76,272,857-76,655,784	LOH	382928	q22.2	6.74018808	9.219858156
chr13:76,655,784-77,103,671	llelic Imbalanc	447888	q22.2	0	29.44444444
chr13:77,103,671-78,797,791	LOH	1694121	q22.2 - q22.3	0.60096097	13.04347826
chr13:78,797,791-84,777,851	llelic Imbalanc	5980061	q22.3 - q31.1	5.53561336	23.2221523
chr13:49,068,958-115,169,878	CN Loss	66100921	q14.2 - q34	3.77743003	16.50950457
chr13:84,777,851-85,499,559	LOH	721709	q31.1	1.08908312	12.29050279
chr13:85,499,559-85,619,524	llelic Imbalanc	119966	q31.1	0	34.7826087
chr13:85,619,524-91,539,625	LOH	5920102	q31.1 - q31.3	0.66784334	10.76030928
chr13:91,539,625-92,026,884	llelic Imbalanc	487260	q31.3	0.97053107	28.94736842
chr13:92,026,884-92,408,296	LOH	381413	q31.3	1.58332722	9.292035398
chr13:92,408,296-93,097,112	llelic Imbalanc	688817	q31.3	0.82910385	31.4516129
chr13:93,097,112-94,103,545	LOH	1006434	q31.3	8.24605314	11.0864745
chr13:94,103,545-95,032,452	llelic Imbalanc	928908	q31.3 - q32.1	0	17.99242424
chr13:95,032,452-99,438,891	LOH	4406440	q32.1 - q32.3	0.61441904	12.90824261
chr13:99,438,891-99,531,521	llelic Imbalanc	92631	q32.3	0	48.14814815
chr13:99,531,521-102,882,149	LOH	3350629	q32.3 - q33.1	9.01266867	12.53196931
chr13:102,882,149-103,226,012	llelic Imbalanc	343864	q33.1	0	28.81355932
chr13:103,226,012-103,560,636	LOH	334625	q33.1	0	7.03125
chr13:103,560,636-103,769,019	llelic Imbalanc	208384	q33.1	0	21.9047619
chr13:103,769,019-104,718,783	LOH	949765	q33.1	1.11059168	8.767123288
chr13:104,718,783-105,414,346	llelic Imbalanc	695564	q33.1 - q33.2	0.40902118	24.06015038
chr13:105,414,346-114,113,416	LOH	8699071	q33.2 - q34	6.01803411	14.2578125
chr13:114,113,416-114,470,598	llelic Imbalanc	357183	q34	0	40
chr13:114,470,598-115,169,878	LOH	699281	q34	7.4460874	7.608695652
chr14:20,511,673-20,579,891	LOH	68219	q11.2	100	0
chr14:22,592,044-22,737,548	LOH	145505	q11.2	100	1.886792453
chr14:25,089,988-25,261,251	LOH	171264	q12	0	2.597402597
chr14:26,912,365-27,279,269	LOH	366905	q12	2.11581231	1.204819277
chr14:29,078,216-29,186,440	LOH	108225	q12	6.25462005	3.333333333

chr14:29,221,922-29,268,878	CN Gain	46957	q12	0	20
chr14:29,299,892-29,628,425	LOH	328534	q12	0	0
chr14:33,376,396-33,530,352	LOH	153957	q13.1	0	1.333333333
chr14:34,137,271-34,229,381	LOH	92111	q13.1	0	0
chr14:37,203,074-37,279,847	LOH	76774	q13.3	0	1.449275362
chr14:38,735,885-38,764,643	CN Gain	28759	q21.1	0	44.44444444
chr14:39,965,687-40,216,148	LOH	250462	q21.1	0	2.631578947
chr14:42,327,762-42,582,279	LOH	254518	q21.1	0	1.639344262
chr14:44,067,823-44,090,818	CN Gain	22996	q21.2	100	0
chr14:43,317,743-44,889,339	LOH	1571597	q21.1 - q21.2	50.6747917	1.555555556
chr14:46,230,972-46,867,414	LOH	636443	q21.2	4.46922108	1.754385965
chr14:47,721,965-47,776,772	CN Gain	54808	q21.3	0	26.31578947
chr14:48,200,686-48,433,263	LOH	232578	q21.3	19.556534	3.03030303
chr14:48,509,647-49,307,130	LOH	797484	q21.3	1.27463532	5.02283105
chr14:49,575,047-50,461,368	LOH	886322	q21.3	64.1060067	3.913043478
chr14:52,760,439-52,912,690	LOH	152252	q22.1	0.26272405	2.777777778
chr14:54,426,116-54,447,476	CN Gain	21361	q22.2	0	9.090909091
chr14:54,447,476-54,660,628	LOH	213153	q22.2	0	3
chr14:54,778,159-55,106,064	LOH	327906	q22.2	0	0.943396226
chr14:56,087,772-57,525,937	llelic Imbalanc	1438166	q22.3	1.39691899	25.95907928
chr14:57,525,937-57,923,829	LOH	397893	q22.3	0	6.25
chr14:57,923,829-58,237,476	llelic Imbalanc	313648	q22.3 - q23.1	2.63895398	35.09933775
chr14:58,237,476-58,404,777	LOH	167302	q23.1	0.61505909	3.448275862
chr14:58,404,777-61,874,549	llelic Imbalanc	3469773	q23.1	3.02403155	32.30309073
chr14:61,874,549-61,998,619	LOH	124071	q23.1	14.9705811	2.197802198
chr14:61,998,619-63,144,261	llelic Imbalanc	1145643	q23.1 - q23.2	0.38179466	28.05139186
chr14:63,144,261-63,910,518	LOH	766258	q23.2	0.11536599	5.448717949
chr14:63,910,518-65,138,384	llelic Imbalanc	1227867	q23.2 - q23.3	0.45860053	27.85234899
chr14:56,087,772-73,995,963	CN Loss	17908192	q22.3 - q24.3	1.348517	24.92129246
chr14:65,138,384-65,353,793	LOH	215410	q23.3	0	3.529411765
chr14:65,353,793-70,591,668	llelic Imbalanc	5237876	q23.3 - q24.2	0.99985204	28.98453262
chr14:70,591,668-70,741,963	LOH	150296	q24.2	1.0313051	1.538461538
chr14:70,741,963-71,309,599	llelic Imbalanc	567637	q24.2	0	29.72972973
chr14:71,309,599-71,727,412	LOH	417814	q24.2	0	5.109489051
chr14:71,727,412-72,700,882	llelic Imbalanc	973471	q24.2	0	29.49852507
chr14:72,700,882-72,939,189	LOH	238308	q24.2	0	8.73015873
chr14:72,939,189-73,995,963	llelic Imbalanc	1056775	q24.2 - q24.3	2.25100163	24.65753425
chr14:74,038,661-74,668,225	LOH	629565	q24.3	8.68632895	10.14492754
chr14:74,668,225-77,320,605	llelic Imbalanc	2652381	q24.3	0.32099473	31.33514986
chr14:77,320,605-77,721,741	LOH	401137	q24.3	0	6.622516556
chr14:77,721,741-78,694,082	llelic Imbalanc	972342	q24.3	1.51963149	30.43478261
chr14:78,694,082-79,498,574	LOH	804493	q24.3 - q31.1	1.41617319	8.529411765
chr14:74,038,661-85,765,434	CN Loss	11726774	q24.3 - q31.3	1.24450264	25.68691707
chr14:79,498,574-83,225,468	llelic Imbalanc	3726895	q31.1	1.05135805	29.94732882
chr14:83,225,468-83,352,788	LOH	127321	q31.1	0	5.208333333
chr14:83,352,788-85,765,434	llelic Imbalanc	2412647	q31.1 - q31.3	0.7207025	27.25030826
chr14:85,765,434-88,636,170	LOH	2870737	q31.3	2.71505286	13.95555556

chr14:93,787,673-94,202,645	LOH	414973	q32.12	1.26586854	0
chr14:88,636,170-102,622,020	CN Gain	13985851	q31.3 - q32.31	1.75036912	21.54479918
chr14:95,992,348-96,377,865	LOH	385518	q32.13 - q32.2	0	0.518134715
chr14:97,316,245-97,583,470	LOH	267226	q32.2	0	0
chr14:98,351,534-98,612,506	LOH	260973	q32.2	0	2.054794521
chr14:98,648,683-98,762,062	LOH	113380	q32.2	0.97637129	1.098901099
chr14:99,810,156-100,098,103	LOH	287948	q32.2	0	2.666666667
chr14:102,332,951-102,622,020	LOH	289070	q32.31	1.27962528	3.278688525
chr14:102,622,020-103,680,970	llelic Imbalanc	1058951	32.31 - q32.3	1.33925115	21.31979695
chr14:102,660,284-105,345,457	CN Gain	2685174	32.31 - q32.3	4.76475817	23.46723044
chr14:105,347,255-106,335,832	CN Gain	988578	q32.33	64.7030024	3.947368421
chr14:105,347,255-107,349,540	LOH	2002286	q32.33	77.4571552	2.985074627
chr14:106,335,832-106,751,237	High Copy Gai	415406	q32.33	100	0
chr14:106,751,237-107,150,507	CN Gain	399271	q32.33	100	2.586206897
chr15:27,694,105-27,931,492	LOH	237388	q12	4.38271683	1.438848921
chr15:33,353,070-33,490,023	LOH	136954	q13.3	100	1.5625
chr15:34,141,450-34,621,715	LOH	480266	q14	100	2.013422819
chr15:35,105,846-35,320,741	LOH	214896	q14	0.47232369	1.538461538
chr15:36,904,043-37,206,432	LOH	302390	q14	0	6.060606061
chr15:39,320,698-39,622,640	LOH	301943	q14	1.65164171	4.591836735
chr15:39,798,445-40,058,187	LOH	259743	q14	0	0.8
chr15:42,838,582-44,067,268	llelic Imbalanc	1228687	q15.2 - q15.3	18.4515002	26.85185185
chr15:44,067,268-45,272,041	LOH	1204774	q15.3 - q21.1	14.4013022	11.06382979
chr15:45,272,041-45,685,280	llelic Imbalanc	413240	q21.1	27.5254756	28.08988764
chr15:45,685,280-54,774,377	LOH	9089098	q21.1 - q21.3	0.69576769	14.0970516
chr15:42,838,582-60,863,213	CN Loss	18024632	q15.2 - q22.2	7.15728383	15.46339825
chr15:54,774,377-59,911,575	llelic Imbalanc	5137199	q21.3 - q22.2	13.3260972	17.14770798
chr15:59,911,575-60,863,213	LOH	951639	q22.2	2.97245381	12.72264631
chr15:61,013,645-61,055,235	CN Loss	41591	q22.2	0	20
chr15:62,125,913-62,553,303	LOH	427391	q22.2	1.99489927	4.504504505
chr15:63,901,300-65,283,419	LOH	1382120	q22.31	1.07516068	14.04109589
chr15:63,901,300-65,352,999	CN Loss	1451700	q22.31	1.02362818	14.64968153
chr15:68,683,795-69,358,492	LOH	674698	q23	1.89759255	5.319148936
chr15:69,358,492-69,501,350	llelic Imbalanc	142859	q23	40.0999594	24.3902439
chr15:69,358,492-69,501,350	CN Loss	142859	q23	40.0999594	24.3902439
chr15:69,616,541-70,153,555	LOH	537015	q23	0	7.344632768
chr15:70,153,555-70,702,863	llelic Imbalanc	549309	q23	0	25.71428571
chr15:70,702,863-73,024,858	LOH	2321996	q23 - q24.1	2.12700716	14.43123939
chr15:69,827,541-74,949,121	CN Loss	5121581	q23 - q24.1	2.05255019	15.77335375
chr15:73,024,858-73,923,170	llelic Imbalanc	898313	q24.1	0.01202255	18.22429907
chr15:73,923,170-74,949,121	LOH	1025952	q24.1	5.42189637	12.56038647
chr15:78,725,652-79,742,712	LOH	1017061	q25.1	0.95500757	4.166666667
chr15:79,855,841-80,051,001	LOH	195161	q25.1	16.0652798	0
chr15:81,085,547-81,334,452	LOH	248906	q25.1	0	2.272727273
chr15:87,162,729-87,464,482	LOH	301754	q25.3	4.93648779	3.092783505
chr15:92,570,203-93,061,173	LOH	490971	q26.1	0.82815651	2.008032129
chr15:94,216,278-94,522,917	LOH	306640	q26.1 - q26.2	0	1.694915254

chr15:94,845,492-95,073,095	LOH	227604	q26.2	1.36948986	2.5
chr15:96,228,222-96,563,272	LOH	335051	q26.2	0	2.727272727
chr15:96,968,949-97,290,450	LOH	321502	q26.2	0	5
chr15:97,804,064-98,032,228	LOH	228165	q26.2	24.1690188	0
chr15:98,213,074-98,353,919	LOH	140846	q26.2	11.4885157	0
chr15:100,873,543-101,077,343	LOH	203801	q26.3	0.27134446	1.204819277
chr16:1,469,646-1,986,971	LOH	517326	p13.3	19.4790509	2.325581395
chr16:6,113,607-6,140,333	CN Gain	26727	p13.3	0	39.47368421
chr16:6,263,632-6,361,339	LOH	97708	p13.3	0	6.504065041
chr16:6,232,137-6,677,059	CN Gain	444923	p13.3	8.97820292	17.60154739
chr16:6,566,090-6,653,856	LOH	87767	p13.3	19.0768635	5.833333333
chr16:6,751,851-6,855,695	LOH	103845	p13.3	0	1.923076923
chr16:6,855,695-6,873,044	CN Gain	17350	p13.3	0	50
chr16:6,891,431-6,957,911	LOH	66481	p13.3	0	1.219512195
chr16:7,006,372-7,264,533	CN Gain	258162	p13.3	0.34009785	30.79268293
chr16:7,449,788-7,505,230	CN Gain	55443	p13.3	0	44.15584416
chr16:7,540,372-7,605,417	LOH	65046	p13.3	0	0
chr16:7,609,753-7,621,065	CN Gain	11313	p13.3	0	53.33333333
chr16:7,829,301-8,075,639	LOH	246339	p13.3 - p13.2	0	2.4
chr16:10,030,456-10,252,178	LOH	221723	p13.2	0	1.739130435
chr16:12,791,175-13,046,372	LOH	255198	p13.12	0	4.444444444
chr16:14,124,789-15,959,283	LOH	1834495	13.12 - p13.1	16.7914422	6.52173913
chr16:16,071,877-16,103,205	CN Gain	31329	p13.11	100	0
chr16:16,071,877-16,103,205	LOH	31329	p13.11	100	0
chr16:16,812,394-17,133,107	LOH	320714	p12.3	7.0910752	1.315789474
chr16:19,842,748-20,140,535	LOH	297788	p12.3	9.2723994	2.777777778
chr16:20,719,667-21,256,111	LOH	536445	p12.3 - p12.2	0	2.459016393
chr16:27,971,365-28,490,511	LOH	519147	p12.1 - p11.2	21.4149777	2.777777778
chr16:33,564,246-35,220,544	LOH	1656299	p11.2 - p11.1	75.3523219	7.446808511
chr16:35,064,310-35,220,544	CN Loss	156235	p11.1	6.54146985	8.823529412
chr16:46,464,489-48,606,631	LOH	2142143	q11.2 - q12.1	0.4073026	9.433962264
chr16:48,606,631-50,473,725	llelic Imbalanc	1867095	q12.1	0.12077592	27.25409836
chr16:50,473,725-51,449,497	LOH	975773	q12.1	0.9176324	11.2716763
chr16:51,449,497-51,705,624	llelic Imbalanc	256128	q12.1	0.06324987	44.73684211
chr16:51,705,624-52,244,986	LOH	539363	q12.1	0	9.782608696
chr16:52,244,986-53,149,249	llelic Imbalanc	904264	q12.1 - q12.2	0.16510683	23.44322344
chr16:53,149,249-53,992,187	LOH	842939	q12.2	0	10.9375
chr16:53,992,187-59,181,286	llelic Imbalanc	5189100	q12.2 - q21	2.53535344	19.17900404
chr16:59,181,286-59,804,502	LOH	623217	q21	0.3844574	12.99435028
chr16:59,804,502-71,823,060	llelic Imbalanc	12018559	q21 - q22.2	6.23177922	18.72713972
chr16:46,802,098-90,354,753	CN Loss	43552656	q11.2 - q24.3	4.61440066	18.14266698
chr16:71,823,060-73,401,200	LOH	1578141	q22.2 - q22.3	2.00267403	13.54466859
chr16:73,401,200-75,236,960	llelic Imbalanc	1835761	q22.3 - q23.1	8.4170044	21.89473684
chr16:75,236,960-78,385,213	LOH	3148254	q23.1	4.05545552	14.96492595
chr16:78,385,213-79,216,950	llelic Imbalanc	831738	q23.1 - q23.2	15.9841392	23.54048964
chr16:79,216,950-79,946,937	LOH	729988	q23.2	0.93289333	12.98342541
chr16:79,946,937-81,407,914	llelic Imbalanc	1460978	q23.2	7.09052915	20.37325039

chr16:81,407,914-82,485,361	LOH	1077448	q23.2 - q23.3	4.50221681	11.66253102
chr16:82,485,361-87,332,802	llelic Imbalanc	4847442	q23.3 - q24.2	2.2946334	18.63136863
chr16:87,332,802-87,983,038	LOH	650237	q24.2	23.3056921	11.27819549
chr16:87,983,038-90,354,753	llelic Imbalanc	2371716	q24.2 - q24.3	9.98227021	21.06666667
chr17:2,245,683-2,284,193	CN Gain	38511	p13.3	6.20358348	0
chr17:0-16,400,065	LOH	16400066	p13.3 - p11.2	9.85548533	2.318024263
chr17:17,328,300-17,999,387	LOH	671088	p11.2	1.22085512	0.675675676
chr17:26,828,947-26,851,927	CN Loss	22981	q11.2	0	50
chr17:27,888,321-29,784,160	LOH	1895840	q11.2	1.2419831	3.594771242
chr17:30,982,378-31,275,526	LOH	293149	q11.2	0	0
chr17:43,575,180-43,655,356	CN Loss	80177	q21.31	100	
chr17:44,100,569-44,137,410	CN Gain	36842	q21.31	100	85.71428571
chr17:44,212,388-44,789,453	CN Gain	577066	q21.31	100	60.6557377
chr17:45,308,008-45,881,002	LOH	572995	q21.32	13.6415041	1.785714286
chr17:51,111,948-51,378,181	LOH	266234	q22	0	2.666666667
chr17:51,651,565-51,988,798	LOH	337234	q22	3.44242705	2.127659574
chr17:53,313,908-53,467,472	LOH	153565	q22	1.0380037	0
chr17:58,326,281-59,304,556	LOH	978276	q23.2	1.8472311	5.945945946
chr17:60,508,077-60,626,147	llelic Imbalanc	118071	q23.2	0	25
chr17:60,508,077-60,626,147	CN Gain	118071	q23.2	0	25
chr17:62,361,521-63,075,278	LOH	713758	q23.3 - q24.1	13.6956976	5.494505495
chr17:63,128,160-63,411,336	LOH	283177	q24.1	0	0
chr17:63,423,258-64,183,296	LOH	760039	q24.1	5.55235396	3.125
chr17:64,183,296-64,231,577	CN Gain	48282	q24.1 - q24.2	0	57.14285714
chr17:64,231,577-64,427,885	llelic Imbalanc	196309	q24.2	4.33553396	31.81818182
chr17:64,427,885-64,544,951	LOH	117067	q24.2	0.67056191	1.851851852
chr17:65,633,655-66,269,140	LOH	635486	q24.2	2.2593767	4.672897196
chr17:69,945,340-70,177,880	LOH	232541	q24.3	0	4.411764706
chr17:76,654,922-77,077,172	LOH	422251	q25.3	1.99455299	1.298701299
chr17:78,513,424-78,760,919	LOH	247496	q25.3	99.1175579	3.296703297
chr17:79,299,639-80,249,552	LOH	949914	q25.3	42.5826365	4.651162791
chr18:0-656,165	llelic Imbalanc	656166	p11.32	3.73579816	27.73109244
chr18:656,165-963,813	LOH	307649	p11.32	1.74940191	4.504504505
chr18:0-2,013,650	CN Loss	2013651	p11.32	10.2043553	28.83522727
chr18:963,813-2,013,650	llelic Imbalanc	1049838	p11.32	16.7249773	37.18309859
chr18:8,772,908-9,096,954	LOH	324047	p11.22	0	1.550387597
chr18:9,428,748-9,654,223	LOH	225476	p11.22	0	1.492537313
chr18:19,300,675-19,777,232	LOH	476558	q11.2	0.17962175	4.210526316
chr18:20,312,623-20,800,633	LOH	488011	q11.2	0.60244667	0.943396226
chr18:21,149,207-21,655,826	LOH	506620	q11.2	14.3468761	0
chr18:23,442,625-24,024,528	LOH	581904	q11.2	1.09966781	2.34375
chr18:26,001,821-26,764,655	LOH	762835	q12.1	0	1.442307692
chr18:29,088,184-29,365,700	LOH	277517	q12.1	0	2.380952381
chr18:30,261,740-30,981,839	LOH	720100	q12.1	1.08693388	1.764705882
chr18:31,082,252-31,941,189	LOH	858938	q12.1	0	1.5625
chr18:32,073,610-32,456,652	LOH	383043	q12.1	0	4.081632653
chr18:33,105,718-33,650,846	LOH	545129	q12.2	0	1.62601626

chr18:33,916,234-34,243,125	LOH	326892	q12.2	0	3.649635036
chr18:34,902,219-35,526,899	LOH	624681	q12.2	0.09700967	6.41025641
chr18:36,827,656-37,291,137	LOH	463482	q12.2 - q12.3	0	6
chr18:38,769,302-39,037,759	LOH	268458	q12.3	1.27506454	1.470588235
chr18:44,530,619-44,542,951	CN Gain	12333	q21.1	100	
chr18:44,542,951-44,558,565	CN Loss	15615	q21.1	100	
chr18:44,558,565-44,586,469	CN Gain	27905	q21.1	100	64.70588235
chr18:45,300,257-45,614,804	LOH	314548	q21.1	0	3.03030303
chr18:46,497,173-47,041,264	LOH	544092	q21.1	1.39884688	4.316546763
chr18:51,964,554-52,689,234	LOH	724681	q21.2	0.67850638	1.438848921
chr18:54,980,051-55,280,097	LOH	300047	q21.31	0	4.379562044
chr18:57,821,717-58,120,846	LOH	299130	q21.32	4.53984736	2.298850575
chr18:61,514,125-61,797,358	LOH	283234	q21.33 - q22.1	14.8443155	2.298850575
chr18:62,825,423-63,622,659	LOH	797237	q22.1	1.46443462	3.720930233
chr18:64,868,999-65,087,916	LOH	218918	q22.1	5.28739203	4.109589041
chr18:65,639,149-65,852,422	LOH	213274	q22.1	33.9663249	0
chr18:66,606,842-66,891,284	LOH	284443	q22.1 - q22.2	39.4910034	2.298850575
chr18:67,468,268-68,128,120	LOH	659853	q22.2	100	4.117647059
chr18:69,167,573-69,182,407	CN Loss	14835	q22.3	100	0
chr18:69,167,573-69,182,407	LOH	14835	q22.3	100	0
chr18:69,411,772-69,762,253	LOH	350482	q22.3	4.26128663	2
chr18:72,388,125-72,941,088	LOH	552964	q22.3	0	3.431372549
chr18:74,849,984-74,979,214	LOH	129231	q23	14.5090149	2.469135802
chr18:76,148,803-76,966,006	LOH	817204	q23	21.9720192	2.43902439
chr19:5,531,968-6,204,114	LOH	672147	p13.3	0.0743886	9.821428571
chr19:7,302,221-7,365,804	High Copy Gai	63584	p13.2	0	
chr19:11,310,026-11,873,322	LOH	563297	p13.2	0.63004175	5.263157895
chr19:12,089,968-12,619,309	LOH	529342	p13.2	10.5181726	2.631578947
chr19:14,940,554-15,025,569	LOH	85016	p13.12	0	1.587301587
chr19:15,747,622-16,137,393	LOH	389772	p13.12	23.5728158	2.48447205
chr19:20,661,523-23,727,670	LOH	3066148	p12	63.6739856	13.75358166
chr19:20,661,523-24,507,166	CN Loss	3845644	p12 - p11	51.2519233	14.25598335
chr19:23,727,670-24,053,745	llelic Imbalanc	326076	p12	4.33366557	22.52252252
chr19:24,053,745-24,507,166	LOH	453422	p12 - p11	0.9917935	10.52631579
chr19:28,271,107-28,314,208	llelic Imbalanc	43102	q11	0	22.72727273
chr19:28,271,107-28,314,208	CN Loss	43102	q11	0	22.72727273
chr19:31,231,671-31,790,208	LOH	558538	q12	0.93870236	1.818181818
chr19:32,074,457-32,419,687	LOH	345231	q12 - q13.11	1.07174927	1.388888889
chr19:37,338,915-37,688,693	LOH	349779	q13.12	0	1.25
chr19:37,688,693-37,728,646	CN Gain	39954	q13.12	0	0
chr19:37,728,646-38,476,075	LOH	747430	13.12 - q13.1	10.7436292	1.470588235
chr19:48,531,330-48,550,431	CN Gain	19102	q13.33	100	64.70588235
chr19:52,271,285-52,637,259	LOH	365975	q13.41	98.2720084	2.380952381
chr19:56,897,372-57,069,033	LOH	171662	q13.43	0	1.449275362
chr19:58,776,928-59,128,983	LOH	352056	q13.43	63.3423755	2.53164557
chr20:7,008,882-7,252,508	LOH	243627	p12.3	0	0
chr20:7,266,150-7,512,943	LOH	246794	p12.3	2.10459778	1.234567901

chr20:9,290,166-9,489,792	LOH	199627	p12.2	0.73587609	0
chr20:13,331,050-13,705,865	LOH	374816	p12.1	0	2.352941176
chr20:19,261,772-19,720,529	LOH	458758	p11.23	0	0.549450549
chr20:21,735,572-22,288,385	LOH	552814	p11.22	0	0
chr20:24,685,220-25,020,338	LOH	335119	p11.21	0.20470401	2.105263158
chr20:32,506,659-33,904,283	LOH	1397625	q11.22	0	2.232142857
chr20:35,500,311-36,250,024	LOH	749714	q11.23	0.75202111	4.21686747
chr20:36,267,861-36,529,039	LOH	261179	q11.23	0	4.255319149
chr20:37,430,302-37,878,582	LOH	448281	q11.23 - q12	0	0
chr20:40,580,314-40,762,577	LOH	182264	q12	0	1.298701299
chr20:42,290,151-49,473,305	LOH	7183155	13.12 - q13.1	3.66552074	1.463963964
chr20:54,533,249-55,341,544	LOH	808296	q13.2 - q13.31	0.20611287	0.854700855
chr21:15,645,804-15,765,839	LOH	120036	q11.2	0	0
chr21:16,496,183-16,743,689	LOH	247507	q21.1	1.88803504	0
chr21:17,012,513-17,357,311	LOH	344799	q21.1	0	3.75
chr21:21,204,658-21,305,645	LOH	100988	q21.1	16.2664501	0
chr21:21,305,645-21,340,518	llelic Imbalanc	34874	q21.1	97.7403722	20
chr21:21,305,645-21,340,518	CN Loss	34874	q21.1	97.7403722	20
chr21:25,796,375-26,182,615	LOH	386241	q21.2	0	4.225352113
chr21:26,491,966-26,773,763	LOH	281798	q21.2	0	4.494382022
chr21:27,169,498-27,397,715	LOH	228218	q21.3	2.07302699	3.03030303
chr21:29,724,961-30,591,494	LOH	866534	q21.3	0	1
chr21:31,487,808-31,754,207	LOH	266400	q21.3 - q22.11	2.06607382	3.333333333
chr21:33,864,893-34,265,072	LOH	400180	q22.11	0	1.265822785
chr21:38,152,672-38,598,455	LOH	445784	q22.13	1.1339598	4.545454545
chr21:38,717,146-38,935,217	LOH	218072	q22.13	0	2.564102564
chr21:39,215,142-39,449,998	LOH	234857	q22.13	0	2.985074627
chr21:41,536,547-41,662,113	LOH	125567	q22.2	18.1346861	1.408450704
chr21:44,157,139-44,506,860	LOH	349722	q22.3	1.02281533	3.092783505
chr21:45,341,826-45,864,794	LOH	522969	q22.3	1.79800676	1.941747573
chr22:17,912,112-18,425,580	LOH	513469	q11.21	3.62963223	3.921568627
chr22:18,597,414-19,151,954	LOH	554541	q11.21	93.6361669	1.219512195
chr22:20,935,808-21,317,424	LOH	381617	q11.21	38.0903841	2.97029703
chr22:22,836,456-23,240,129	CN Gain	403674	q11.22	100	20.58823529
chr22:23,648,833-23,994,163	LOH	345331	q11.23	20.1711406	4.347826087
chr22:31,383,125-32,338,693	LOH	955569	q12.2 - q12.3	0.09156857	0.609756098
chr22:32,680,277-32,707,675	CN Gain	27399	q12.3	0	75
chr22:34,871,898-34,981,563	LOH	109666	q12.3	1.11430265	2.857142857
chr22:35,257,038-36,679,516	CN Loss	1422479	q12.3	9.52605242	13.54401806
chr22:35,257,038-36,679,516	LOH	1422479	q12.3	9.52605242	13.54401806
chr22:36,679,516-36,686,654	CN Gain	7139	q12.3	0	
chr22:36,686,654-37,722,336	llelic Imbalanc	1035683	q12.3 - q13.1	1.22296226	17.1314741
chr22:36,686,654-46,563,512	CN Loss	9876859	q12.3 - q13.31	7.4469634	14.73684211
chr22:37,742,992-45,762,288	LOH	8019297	q13.1 - q13.31	7.06755306	13.91524352
chr22:45,762,288-46,563,512	llelic Imbalanc	801225	q13.31	19.481693	19.01840491
chr22:46,563,512-46,565,687	ozygous Copy	2176	q13.31	0	100
chr22:46,565,687-47,305,973	LOH	740287	q13.31	1.90007646	10.34482759

chr22:47,305,973-48,221,007	llelic Imbalanc	915035	q13.31	1.45524647	23.40425532
chr22:46,565,687-51,304,566	CN Loss	4738880	13.31 - q13.3	6.14143134	15.7708628
chr22:48,221,007-51,304,566	LOH	3083560	13.31 - q13.3	8.55028232	14.05049396

* Chart has been modified for space
